# Neurotoxic potential of reactive astrocytes in canine distemper demyelinating leukoencephalitis

**DOI:** 10.1038/s41598-019-48146-9

**Published:** 2019-08-12

**Authors:** J. Klemens, M. Ciurkiewicz, E. Chludzinski, M. Iseringhausen, D. Klotz, V. M. Pfankuche, R. Ulrich, V. Herder, C. Puff, W. Baumgärtner, A. Beineke

**Affiliations:** 1 Department of Pathology, University of Veterinary Medicine Hanover, Hannover, Germany; 2 Department of Experimental Animal Facilities and Biorisk Management, Friedrich-Loeffler-Institut, Greifswald, Insel Riems Germany; 3 Center for Systems Neuroscience, Hannover, Germany

**Keywords:** Diseases of the nervous system, Glial biology

## Abstract

Canine distemper virus (CDV) causes a fatal demyelinating leukoencephalitis in young dogs resembling human multiple sclerosis. Astrocytes are the main cellular target of CDV and undergo reactive changes already in pre-demyelinating brain lesions. Based on their broad range of beneficial and detrimental effects in the injured brain reactive astrogliosis is in need of intensive investigation. The aim of the study was to characterize astrocyte plasticity during the course of CDV-induced demyelinating leukoencephalitis by the aid of immunohistochemistry, immunofluorescence and gene expression analysis. Immunohistochemistry revealed the presence of reactive glial fibrillary acidic protein (GFAP)^+^ astrocytes with increased survivin and reduced aquaporin 4, and glutamine synthetase protein levels, indicating disturbed blood brain barrier function, glutamate homeostasis and astrocyte maladaptation, respectively. Gene expression analysis revealed 81 differentially expressed astrocyte-related genes with a dominance of genes associated with neurotoxic A1-polarized astrocytes. Accordingly, acyl-coA synthetase long-chain family member 5^+^/GFAP^+^, and serglycin^+^/GFAP^+^ cells, characteristic of A1-astrocytes, were found in demyelinating lesions by immunofluorescence. In addition, gene expression revealed a dysregulation of astrocytic function including disturbed glutamate homeostasis and altered immune function. Observed findings indicate an astrocyte polarization towards a neurotoxic phenotype likely contributing to lesion initiation and progression in canine distemper leukoencephalitis.

## Introduction

Canine distemper is a fatal disease in dogs, caused by a single-stranded, negative-sense RNA virus of the genus *Morbillivirus*, which is closely related to the human measles virus. The canine distemper virus (CDV) host range includes dogs and other canids, as well as ferrets, raccoons, bears, large felids and as recently described even macaques^1,2^. Infection leads to fatal systemic disease with a variety of clinical signs such as respiratory and gastrointestinal disorders, skin alterations and severe immunosuppression, which favors secondary infections. In the central nervous system (CNS), infection causes demyelinating leukoencephalitis (CDV-DL), which is considered to be a spontaneous animal model for human multiple sclerosis (MS)^3^. CDV-DL appears to be a biphasic process with a direct virus-mediated phase in the beginning and an immune-mediated disease progression in the chronic phase^4–7^ characterized by viral persistence^8^. Previous studies revealed increasing levels of MHC class II molecules within CDV-DL plaques, indicating a fundamental role of immune processes during disease progression^9^.

In demyelinating distemper lesions, the majority (95%) of infected cells have been identified as astrocytes, representing the main target for CDV^10,11^. Moreover, progressive myelin loss in affected dogs is associated with astrocyte hypertrophy, isomorphic gliosis, reactive astrocytes (gemistocytes), and occasionally the formation of astrocytic syncytia^12–14^. However, the functional relevance of astrocyte plasticity in canine distemper remains to be determined. Astrocytic changes are not restricted to morphologic changes, but include alterations in gene expression profiles influencing functional properties of this cell type^15^.

Within the CNS astrocytes are the most abundant cell type. They play fundamental roles in the healthy brain as well as in pathological conditions. For instance, astrocytes give biochemical support to neurons and oligodendrocytes and maintain the blood brain barrier integrity^16^. Furthermore astrocytes regulate water transport through aquaporins^16^, synthesize metabolic substrates such as glycogen, sterols and lipoproteins^17,18^ and support neighboring neurons through the export of glucose and lactate^19^. They are also able to respond to glutamatergic neurotransmission by influencing the tone of arterioles and thus regulate the local blood supply^20^. Another key function is the removal of neurotransmitters, such as glutamate, from the synaptic cleft by specific transporters and subsequent degradation by glutamine synthetase (glutamate glutamine cycle), thus preventing excitotoxic cell death of neurons and myelin producing oligodendrocytes^17,21^. Under disease conditions elevated glutamate levels can occur due to increased release by neurons and/or glial cells or impaired reuptake by astrocytes. Previous studies showed that CDV-infected rat hippocampal neuronal cells produce increased amounts of glutamate leading to neurodegeneration^22^. Inhibition of AMPA/kainate receptors results in reduction of neuronal death^22,23^ following CDV-infection *in vitro* and in experimental autoimmune encephalomyelitis (EAE), a rodent model for demyelinating diseases^23^. Similarly, in MS axonal and oligodendrocyte damage is referred to glutamate excess^24^, demonstrating the importance of glutamate toxicity in neuroinflammatory diseases. Through secretion of growth factors and neurotrophins astrocytes enable remyelination and promote neuronal survival^25^. However, glial scar formation in response to CNS injuries might hinder neuroregeneration. Additionally, astrocytes facilitate CNS recruitment of immune cells by releasing chemoattractant cytokines and activate T cells, thus representing important immune modulators in the CNS^26^. Recent publications describe the polarization of reactive astrocytes based on their gene expression profile into beneficial and detrimental phenotypes. Astrocytes activated by inflammatory stimuli exhibited a gene expression pattern indicating neurotoxic properties (A1-astrocytes), whereas ischemia induces astrocytes with neuroprotective functions (A2-astrocytes)^27,28^.

Whether detrimental or beneficial effects of reactive astrogliosis predominate in the brain of CDV-infected dogs is discussed controversially. Deeper insights into molecular and functional properties are necessary to understand better the specific role of astrocytes in CDV-DL. Therefore, aims of this study were to (i) determine phenotypical changes of astrocytes in demyelinating lesions, (ii) to characterize astrocytic expression pattern by the aid of gene expression analyses in CDV-infected dogs and (iii) to investigate astrocyte polarization regarding the A1/A2-phenotype in canine distemper.

## Materials and Methods

### Ethics statement

The present study was conducted in accordance with the German Animal Welfare Act. The authors confirm that for the purpose of this retrospective pathological study no animals were infected or sacrificed. This study is not an animal experiment since all animals were dead at the time of submission for necropsy in order to investigate the causes of death and disease. All tissues used in this study were collected by one of the authors (WB) during his work at the diagnostic pathology services of the Department of Pathology, University of Veterinary Medicine Hannover, and the Institute of Veterinary Pathology, Justus-Liebig-University Giessen, and all animals were used in previous publications^29–32^. All dog owners provided written consent for the dogs’ tissues to be collected and used for research purposes.

### Animals, histology and neuropathological classification

For histology, histochemistry and immunohistochemistry, cerebellar tissue of five healthy, CDV-negative control dogs (animal no. 1–5) and 29 spontaneously CDV-infected dogs (animal no. 6–34) was investigated. Anamnestic details of dogs used in this study are listed in Supplemental Table S1. Animals died spontaneously or were euthanized due to poor prognosis. Control dogs were obtained from an animal experiment, which was approved and authorized by the local authorities (Niedersächsisches Landesamt für Verbraucherschutz und Lebensmittelsicherheit (LAVES), Oldenburg, Germany, permission number 08A580). After necropsy, CNS tissue was fixed in 10% neutral-buffered formalin, embedded in paraffin and serial sections of 2 µm thickness were prepared for histology and immunohistochemistry. Neuropathological diagnosis was based on hematoxylin and eosin (HE) staining and luxol fast blue-cresyl violet (LFB/KEV) staining for detection of myelin loss. Accordingly, white matter areas were classified into four groups: group 1 included unaffected brains of healthy control dogs; group 2 comprised acute lesions with vacuolization and gliosis; group 3 contained subacute demyelinating lesions without perivascular inflammation; and in group 4 subacute to chronic demyelinating lesions with perivascular inflammation were included^31^.

### Immunohistochemistry

CDV-infection was confirmed by immunohistochemistry (IHC) using a monoclonal CDV-nucleoprotein (CDV-NP) antibody. For confirmation of demyelination, antibodies directed against MBP (myelin sheaths) and Nogo-A (mature oligodendrocytes) were used. In addition, an antibody directed against S100 (astrocytes and oligodendroglial cells) to characterize white matter lesions was included. For characterization of astrocytic alterations, the astrocyte markers glial fibrillary acidic protein (GFAP), aquaporin 4 (AQP4), aldehyde dehydrogenase 1 family member L1 (ALDH1L1), and glutamine synthetase (GS) as well as a marker for the astrocyte-related anti-apoptotic protein survivin were used. For confirmation of gene expression data, antibodies directed against indoleamine 2,3-dioxygenase (IDO), serglycin (SRGN) and acyl-coA synthetase long-chain family member 5 (ACSL5) were selected. Antibody details are summarized in Supplemental Table S2. Briefly, the sections were deparaffinized by Roticlear (Roth) and hydrated through graded alcohols. Afterwards the endogenous peroxidase activity was inhibited by 85% ethanol with H_2_O_2_ (0.5%). Sections were washed in phosphate-buffered saline (PBS) and pretreated with citrate buffer (pH 6.0) for 20 minutes in the microwave (800 W). MBP, GFAP and AQP4 did not receive any pretreatment. Unspecific bindings were blocked with goat normal serum. Subsequently tissue was incubated over night at 4 °C with the primary antibody. For negative controls monoclonal and polyclonal antibodies were substituted with ascites fluid from non-immunized BALB/cJ mice and rabbit normal serum, respectively. Incubation of primary antibodies was followed by incubation with biotinylated secondary antibodies (Vector Laboratories) for 45 minutes at room temperature. Subsequently the avidin-biotin-complex (VECTASTAIN Elite ABC Kit; Vector Laboratories) was incubated for 30 minutes also at room temperature. The positive antigen-antibody reactions were visualized by incubation with 3.3′-diaminobenzidine tetrahydrochloride (DAB) with H_2_O_2_ (0.03%, pH 7.2) for 5 minutes followed by slight counterstaining with Mayer’s hematoxylin (Merck). CDV-NP, Nogo-A, GFAP, GS, ALDH1L1, S100, survivin, SRGN, and ACSL5 expressing cells were counted in cerebellar white matter lesions using a morphometric grid (number of positive cells/0.0625 mm^2^). Astrocytic AQP4 and IDO protein levels and oligodendrocytic MBP positivity were quantified morphometrically. Digital photographs of the lesions were taken in 100x magnification and the region of interest (ROI) was selected manually using the analySIS^®^ 3.2 Software (Soft Imaging Solutions GmbH). Within these ROIs the area of immunopositive structures was measured in relation to the total area (% area).

### Immunohistochemistry double labeling

Double labeling was performed to quantify GFAP and S100 protein levels in ALDH1L1^+^ astrocytes. Mouse monoclonal ALDH1L1-, rabbit polyclonal GFAP- and rabbit polyclonal S100-antibodies were used (Supplemental Table S2). In brief, following chromogenic reaction with DAB for localizing ALDH1L1, sections were washed for 5 minutes in PBS buffer, followed by incubation overnight at 4 °C with anti-GFAP or -S100 antibodies, respectively. After washing, sections were incubated with a biotinylated goat-anti-rabbit antibody (Vector Laboratories, Burlingame, dilution 1:200) for 30 minutes, followed by the avidin-biotin-peroxidase complex (Vector Laboratories) for 30 minutes. Positive reactions were visualized with the Histogreen substrate system (Linaris). Co-localization of ALDH1L1 (brown) and GFAP (green) or S100 (green) was identified either by the presence of both colors in one cell or by green brown mixed color.

### Immunofluorescence double labeling

For verification of astrocytic SRGN and ACSL5 expression, revealed by gene expression analysis and immunohistochemistry, immunofluorescence double staining in combination with GFAP specific antibodies was performed. 2 µm thick, formalin-fixed, paraffin-embedded tissue sections from two control dogs, two dogs with representative, acute (group 2) and two with inflammatory, subacute to chronic (group 4) lesions were used for SRGN and ACSL5 staining. Polyclonal rabbit anti-SRGN (dilution 1:20) and -ACSL5 antibodies (dilution 1:20) together with a polyclonal goat anti-GFAP antibody (dilution 1:200) were used (see Supplemental Table S2). Secondary antibodies were used in a dilution of 1:200. All antibodies were diluted in PBS with 1% bovine serum albumin (BSA, Roth) and 0.1% Triton X (Sigma-Aldrich).

Paraffin sections were dewaxed and rehydrated. For blockade of unspecific binding, the slides were treated with 20% horse normal serum diluted in PBS with 1% BSA and 0.1% of Triton X for 30 minutes. SRGN and ACSL5 antibodies were incubated overnight. After rinsing with PBS the first secondary antibody (Cy2-conjugated donkey anti-rabbit, Abcam) was incubated for 1.5 hours. For double staining, the procedure was simultaneously repeated with 20% horse normal serum for blockade of unspecific bindings, GFAP primary antibody and the appropriate secondary antibody (Cy3-conjugated donkey anti-goat, Jackson ImmunoResearch). Negative controls received serum of non-immunized goats and rabbits, respectively, instead of primary antibodies. For nuclear counterstaining bisbenzimide (H 33258, Sigma-Aldrich) was used in a dilution of 1:100 in double distilled water and incubated for 10 minutes at room temperature. Subsequently sections were mounted with Dako fluorescent mounting medium (Dako Diagnostika). Evaluation was performed qualitatively by detecting co-localization of SRGN and ACSL5 with GFAP antigen in representative CDV lesions.

### Statistical analysis

For statistical analysis of non-normal distributed data obtained by immunohistochemistry the IBM “Statistic Package for Social Sciences” SPSS program for Windows (version 24) was used, employing a Mann-Whitney U-test for two independent samples. *P*-values of less than or equal to 0.05 were considered to show statistically significant differences between CDV groups. Graphs were designed with GraphPad Prism^®^ (GraphPad Software, version 7.04).

### Microarray analysis

For molecular characterization of astrocytic changes, a data set of genes differentially expressed in the cerebellum of CDV-infected dogs obtained in our previous global gene expression analysis was used^32^. Briefly, cerebellar tissues of 14 CDV-infected dogs and 12 control dogs were used and their lesions were characterized and grouped (group 1–4) based on same morphological criteria as used in the present study. Total RNA was isolated from the frozen cerebellar specimens using the RNeasy Lipid Tissue Mini Kit (Qiagen) amplified and labeled employing the 3′IVT express kit (Affymetrix) and hybridized to GeneChip canine genome 2.0 arrays (Affymetrix) as described^32^. Background adjustment, quantile normalization and probe set summarization were performed using the GC-RMA algorithm (Bioconductor *gcrma* for R package, Version 2.3)^33^. MIAME compliant data sets are deposited in the ArrayExpress database (accession number: E-MEXP-3917; http://www.ebi.ac.uk/arrayexpress).

### Characterization of astrocytic gene expression

The present analyses focused on a list of manually selected genes expressed by astrocytes according to peer-reviewed publications^27,34–43^ and genome databases searching for the term ‘astrocyte’ (http://www.networkglia.eu/en/astrocyte; http://amigo.geneontology.org/amigo/search/bioentity?q=astrocyte). Gene identifier (ID) conversion was performed using the Gene ID Conversion Tool (https://david.ncifcrf.gov/conversion.jsp) of *the database for annotation*, *visualization and integrated discovery* (DAVID, version 6.8) with Entrez Gene ID as unified gene identifier and selecting *Canis lupus familiaris* as target species. In order to investigate which of the manually selected astrocyte-related genes (Supplemental Table S3) are differentially expressed during CDV-infection, the data were re-analysed based on the data set obtained in our previous global gene expression analysis^32^ employing independent pair-wise t-tests comparing groups 1–4 followed by adjustment of the *p*-values according to the method described by Benjamini and Hochberg^44^. Significantly differentially expressed genes (DEGs) between CDV-infected and healthy dogs were selected employing a *q*-value ≤ 0.05 cutoff combined with a fold change filter (fold change ≥ 2.0 or ≤−2.0). The fold change was calculated as the ratio of the inverse-transformed arithmetic means of the log2-transformed expression values of CDV-infected versus healthy control dogs. Downregulations are shown as negative reciprocal values. Hierarchical clustering of the astrocyte-associated DEGs (Supplemental Table S4) was performed using TM4 Multi Experiment Viewer with the log2-transformed individual fold change of each dog relative to the mean of the control dogs, employing Euclidean distance and complete linkage to reveal similar expression patterns^45^. DEGs were visualized through a heat map and analyzed for intersections employing a Venn diagram (http://bioinformatics.psb.ugent.be/webtools/Venn/). Gene ontology information was assigned to the hierarchical clusters of DEGs employing the DAVID Functional Annotation Tool. Significantly enriched gene ontology terms were selected from the biological process category of the gene ontology database at a false discovery rate (FDR) of 1.0%^46^.

From a previous publication genes expressed by A1- and A2-astrocytes were extracted^27^ and likewise converted into the respective canine gene identifiers using DAVID Gene ID Conversion Tool (Supplemental Table S5). For characterization of A1-/A2-polarization, only those genes were selected that are uniquely expressed by one subset of reactive astrocytes (A1 or A2). Analyses based on the annotation version number 36 of the canine genome 2.0 array. The relative proportion of A1- compared to A2-related DEGs was compared for each time point employing Fisher’s exact tests (*p*-value ≤ 0.05).

## Results

### Characterization of cerebellar lesions

**Figure 1:**
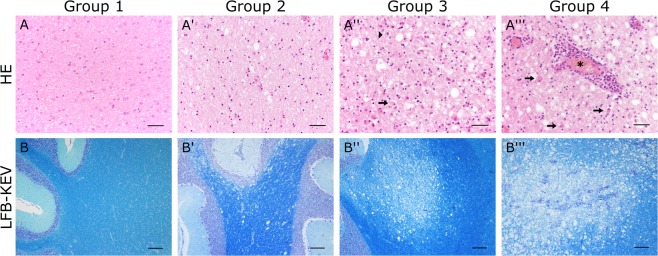
Histologic characterization of the cerebellar white matter of control dogs and canine distemper virus-infected dogs by hematoxylin and eosin (**HE**; **A**–**A′′′**) and luxol fast blue-cresyl violet (**LFB/KEV**) staining (**B**–**B′′′**) **(A**,**B)** Control animal with intact white matter (group 1). (A′,B′) Acute lesion (group 2) with hypercellularity and vacuolization. (**A″**,**B″**) Subacute demyelinated lesion (group 3) with marked hypercellularity, gemistocytic astrocytes (arrow), gitter cells (arrow head) and decreased intralesional LFB/KEV staining. (**A′′′**,**B′′′**) Subacute to chronic lesion (group 4) with marked perivascular cuffs, gemistocytic astrocytes (arrows) and demyelination of the white matter. Asterisk: blood vessel. (**A**–**A′′′**) Scale bar = 50 µm. (**B**–**B′′′**) Scale bar = 200 µm.

**Figure 2:**
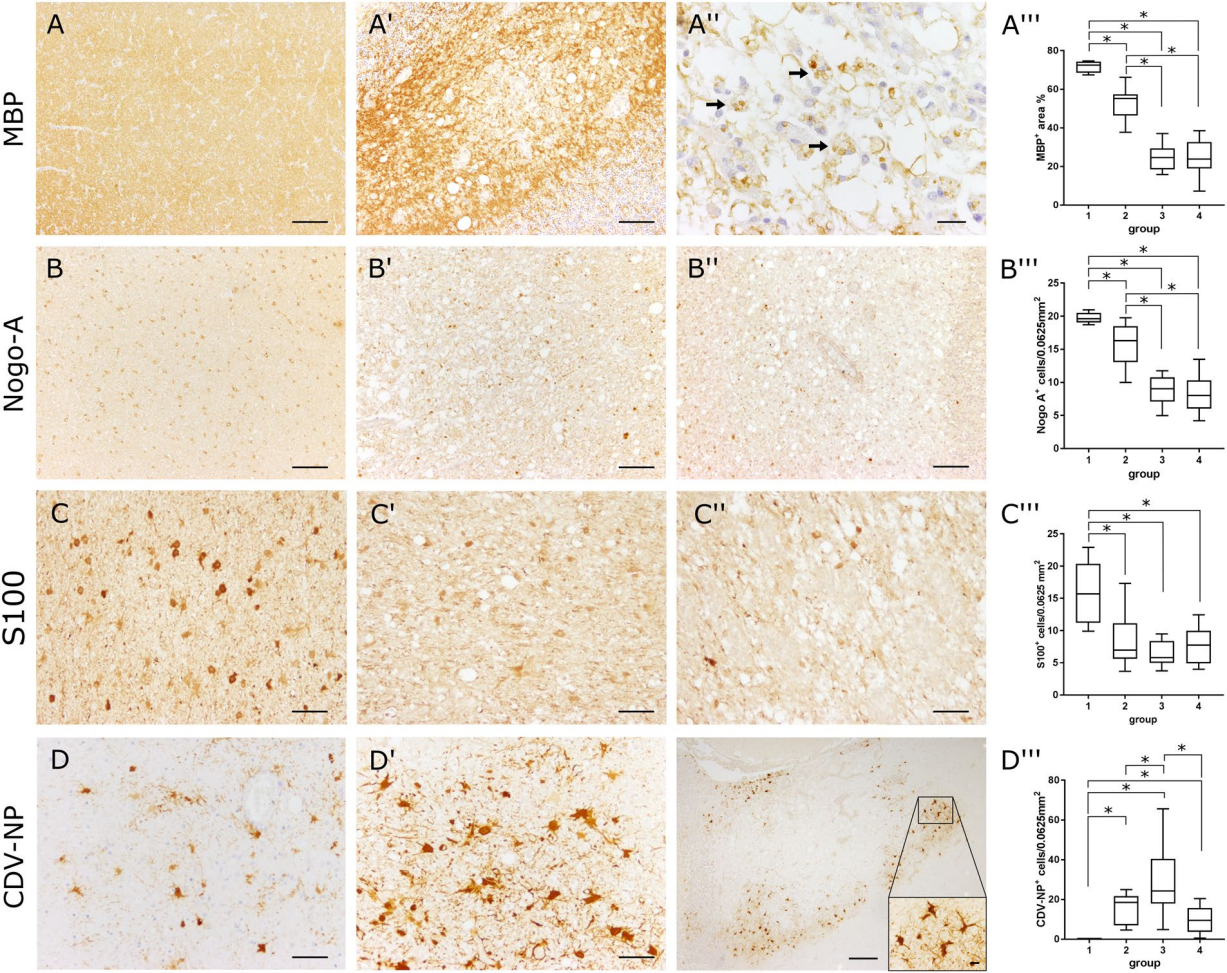
Immunohistochemistry for detecting myelin basic protein (**MBP**; **A**–**A′′′**), neurite outgrowth inhibitor A (**Nogo-A**; **B**,**B′′′**), **S100** (**C**–**C′′′**) and canine distemper virus-nucleoprotein (**CDV-NP**; **D**–**D′′′**). **(A)** Control tissue (group 1). (**A′**) Chronic lesion (group 4) with loss of MBP-positivity. (**A**, **A′**) Scale bar = 100 µm. (**A′′**) Chronic lesion (group 4) with malacia and MBP^+^ material within gitter cells (myelinophages, arrows). Scale bar = 20 µm. (**A”’**) Statistical analysis of MBP-immunohistochemistry shows decreased immunopositive area in demyelinating distemper plaques. (**B**) Control tissue with immunopositive myelinating oligodendrocytes. (**B′**,**B′′**) Subacute lesion (**B′**, group 3) and chronic inflammatory lesion (**B′′**, group 4) with decreased number of Nogo-A^+^ oligodendrocytes. (**B**– **B′′**) Scale bar = 100 µm. (**B′′′**) Statistical analysis of Nogo-A immunohistochemistry shows decreased numbers of myelinating oligodendrocytes in demyelinating distemper lesions. (**C**) Control tissue with immunopositive glial cells. (**C’**,**C”**) Subacute lesion (**C′**, group 3) and chronic inflammatory lesion (**C′′**, group 4) with decreased number of S100^+^ cells (**C**,**C′′**) Scale bar = 20 µm. (**B′′′**) Statistical analysis shows decreased numbers of S100^+^ cells in demyelinating distemper lesions. **(D)** Group 2 lesion: Most CDV-infected cells show astrocyte morphology. (**D’**) Subacute lesion (group 3) with numerous infected astrocytes. (D, D’) Scale bar = 50 µm. (**D′′**) Group 4 lesion with infected astrocytes in the periphery of the inflammatory lesion. Scale bar = 200 µm. Inset: Magnification of CDV-NP^+^ astrocytes. Scale bar = 20 µm. (**D′′′**) Statistical analysis shows most numerous CDV-infected cells in group 3. (**A′′′**,**B′′′**,**C′′′**,**D′′′**) Box and whisker plots display median and quartiles with maximum and minimum values. Significant differences (*p* ≤ 0.05, Mann–Whitney U-test) are labeled by asterisks.

In total, 128 white matter areas were investigated in canine cerebella. Characterization of histopathological alterations was performed using HE and LFB/KEV staining (Fig. 1). Cerebellar tissue of healthy control dogs (group 1) showed no histopathological alterations. Acute lesions (group 2) were characterized by vacuolization (edema of myelin sheaths) and hypercellularity of the white matter due to astro- and microgliosis. In some lesions, intranuclear and/or intracytoplasmic eosinophilic inclusion bodies were found. LFB/KEV-staining revealed no myelin loss or myelinophagia. Group 3 comprised subacute lesions showing demyelination, astrogliosis with gemistocytes, activated macrophages/microglia, gitter cells and single lymphocytes in the neuroparenchyma, but no perivascular inflammation. Demyelination was confirmed by decreased intralesional LFB/KEV-staining and the presence of LFB^+^ structures in the cytoplasm of gitter cells (myelinophagia). In addition to findings observed in group 3 lesions, subacute to chronic lesions (group 4) showed lymphohistiocytic infiltrations in perivascular spaces. Nogo-A, a marker for mature oligodendrocytes, showed progressive loss of reactivity in oligodendroglial processes in CDV lesions (Fig. 2). Myelin changes were also confirmed by MBP-specific immunohistochemistry. Acute lesions (group 2) showed a slight but significant decrease in morphometric MBP density as a consequence of neuropil vacuolization. In subacute and chronic lesions (group 3 and 4) a significant MBP loss compared to early phases (group 2) was observed. Additionally MBP^+^ intracytoplasmic structures were detected in gitter cells of group 3 and 4 lesions, which confirm demyelination and myelinophagia (Fig. 2). S100 protein, which is expressed in cells of the astrocytic and oligodendroglial lineage^47,48^ was found throughout the white matter of control dogs. The number of S100^+^ cells significantly decreased within CDV lesions of all groups (Fig. 2). Control dogs did not show CDV-nucleoprotein in the brain, whereas CDV antigen was found in group 2–4 lesions in varying degrees. Group 2 lesions revealed moderate numbers of CDV-NP^+^ cells intralesionally, which mostly displayed astrocytic morphology. Most CDV-NP^+^ cells were detected in group 3, whereas group 4 revealed a decreased amount of CDV antigen (Fig. 2).

As shown before by experimental infections, CDV-induced leukoencephalitis in dogs develop in a sequential order^49,50^. Acute lesions, characterized by white matter vacuolization and glial infection, can be observed 16–24 days post infection^50–53^. Subacute lesions with demyelination but without perivascular inflammation occur 24–32 days after infection^11,50–53^. Subacute to chronic lesions with demyelination, perivascular lymphohistiocytic cuffs and reduced numbers of CDV^+^ cells can be found after a minimum of 29–63 days post infection in the brain of experimentally infected dogs^11,50–54^.

### Phenotypic changes of astrocytes in demyelinating leukoencephalitis

**Figure 3:**
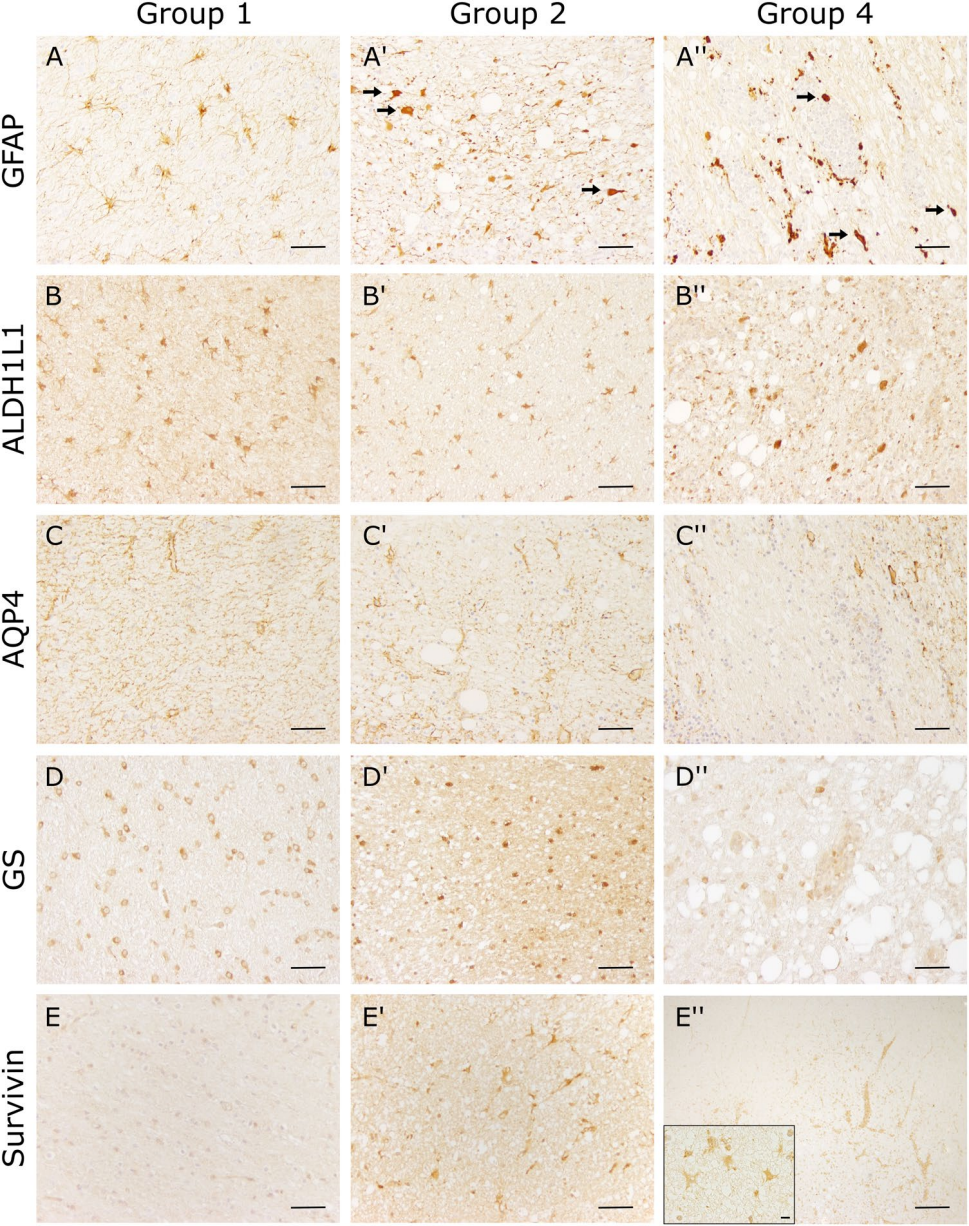
Phenotypical characterization of astrocytes in the cerebellar white matter by immunohistochemistry. Detection of astrocyte markers glial fibrillary acidic protein (**GFAP**; **A**,**A′′**), aldehyde dehydrogenase 1 L1 (**ALDH1L1**; **B**,**B′′**), aquaporin 4 (**AQP4; C**,**C′′**), glutamine synthetase (GS; **D**,**D′′**), and **survivin (E**,**E′′**). (**A**,**E**) Intact white matter of a control animal (group 1). Scale bar = 50 µm. (**A’**,**E’**) Acute distemper lesion (group 2). Arrows = gemistocytic astrocytes. Scale bar = 50 µm. (**A′′**,**D′′**) Subacute to chronic, inflammatory lesion (group 4). Arrows = gemistocytic astrocytes. Scale bar = 50 µm. (**E′′**) Group 4 lesion with perivascular inflammation and increase of intralesional survivin^+^ cells. Scale bar = 200 µm. Inset: Higher magnification. Immunopositive cells show astrocyte morphology. Scale bar = 20 µm.

**Figure 4:**
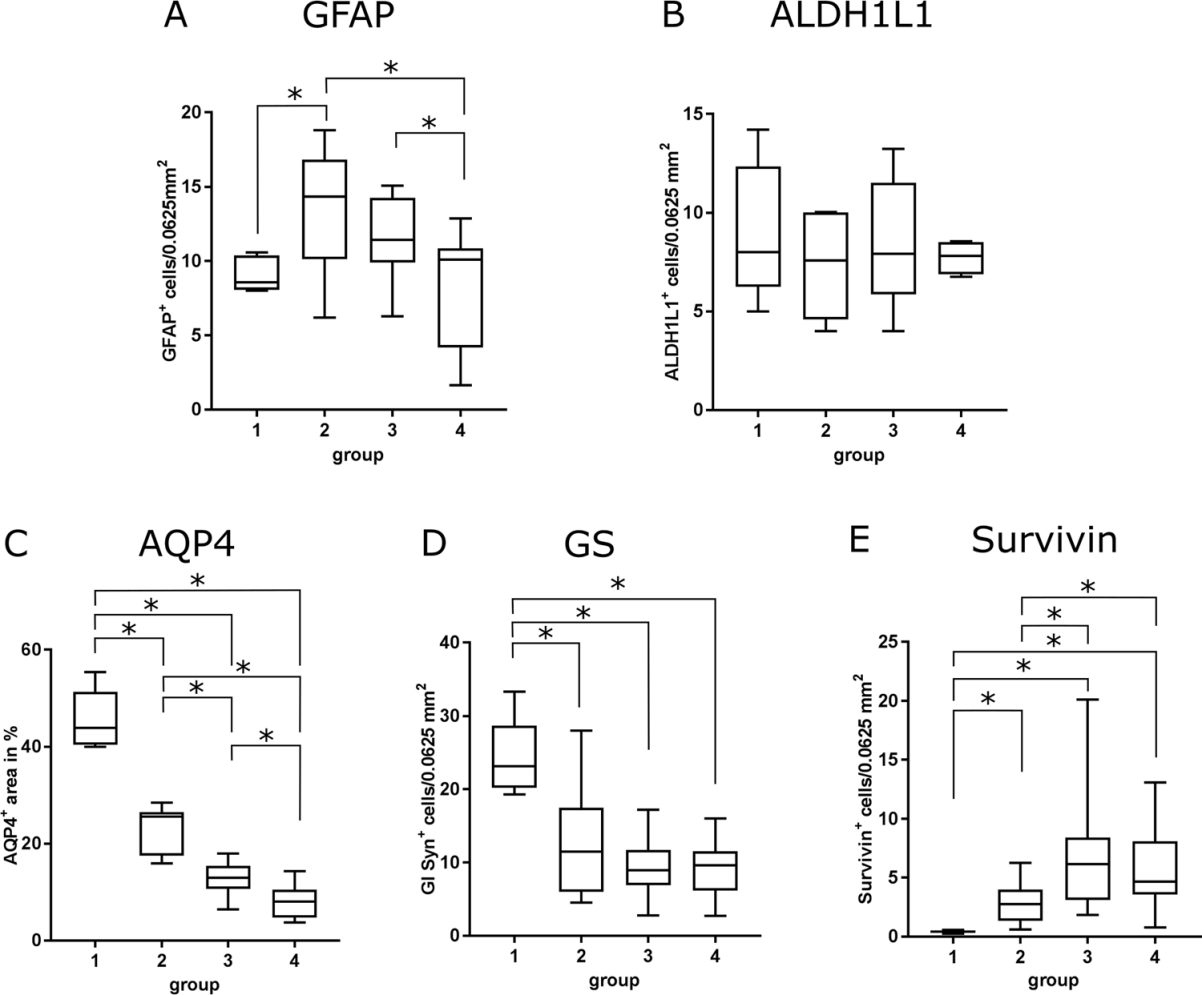
Statistical analysis of immunohistochemical evaluation. (**A**) Increased numbers of glial fibrillary acidic protein (GFAP)^+^ cells in acute distemper lesions. (**B**) Detection of aldehyde dehydrogenase 1 L1 (ALDH1L1) showed no significant differences between control and canine distemper virus-infected dogs. (**C**) Progressive loss of aquaporin 4 (AQP4) in distemper lesions. (**D**) Significant decrease of glutamine synthetase (GS) in all groups of distemper lesions. (**E**) Significant increase in survivin protein levels in all phases of canine distemper virus-induced leukoencephalitis. Box and whisker plots display median and quartiles with maximum and minimum values. Significant differences (*p* ≤ 0.05, Mann–Whitney U-test) are labeled by asterisks.

Astrocytic changes during the disease course were determined by immunohistochemistry using astrocyte markers (GFAP, ALDH1L1, AQP4, GS, survivin). In randomly selected white matter areas of control animals (group 1) regularly distributed GFAP^+^ fibrous astrocytes were detected (Fig. 3). Within acute lesions (group 2), besides fibrous astrocytes also enlarged, plump GFAP^+^ somata (gemistocytes) with a homogenous to finely granulated, light brown, cytoplasmic signal were observed. In group 3 and 4 the number of GFAP^+^ gemistocytes continuously increased, whereas the total number of astrocytes and their processes decreased compared to group 2. Subacute to chronic lesions (group 4) showed a moderate to severe loss of GFAP^+^ astrocytes in the lesion center, while at the periphery an increased number of astrocytes built a demarcation line around demyelinating lesions. Statistically, a significant increase of GFAP^+^ cells was confirmed in acute lesions (group 2), whereas group 3 and 4 lesions did not show significant differences compared to control animals (Fig. 4).

**Figure 5:**
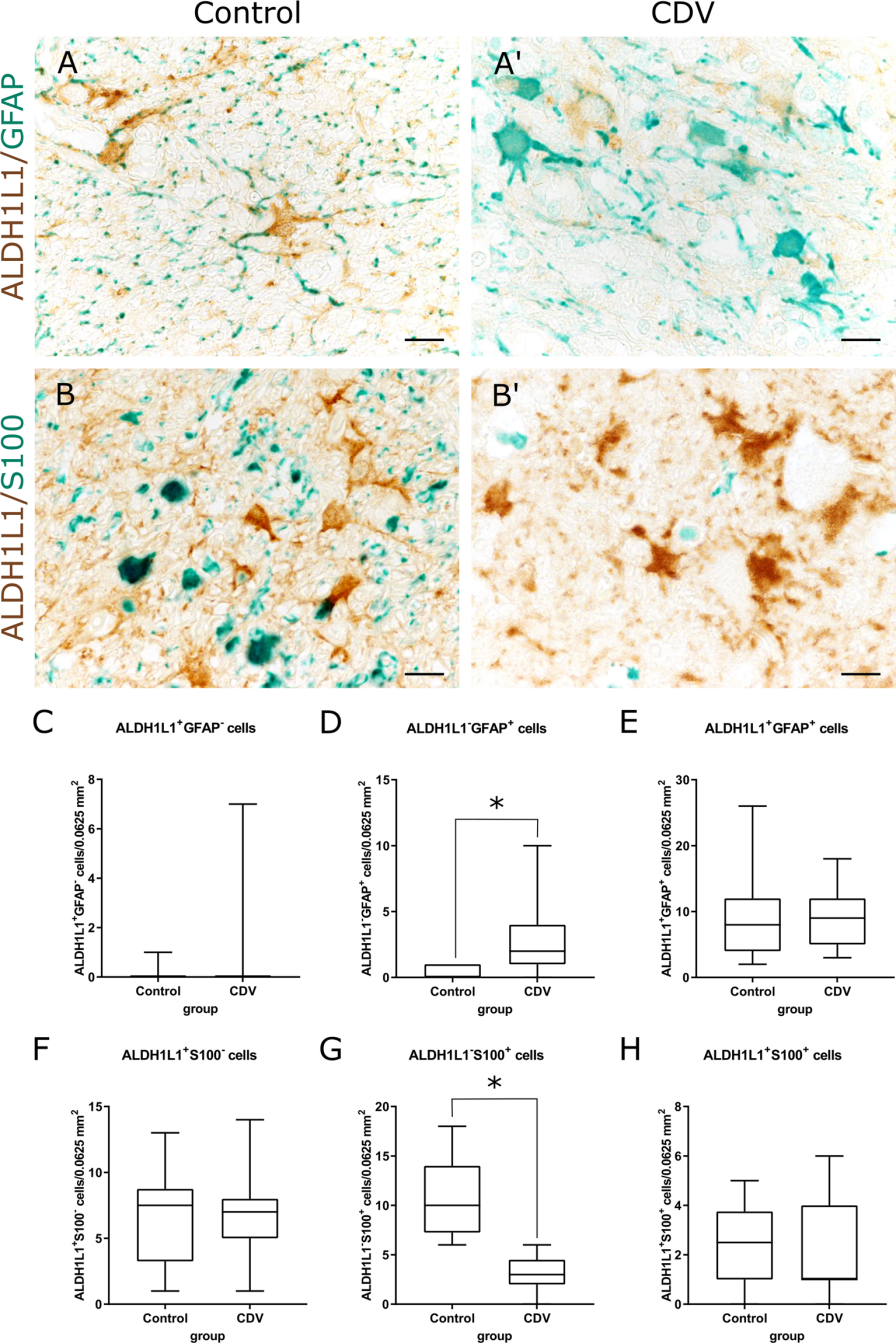
Immunohistochemistry double labeling of astrocytic markers in the cerebellar white matter. (**A**,**A′**) Detection of ALDH1L1 (brown) and GFAP (green) in tissue of a control animal (**A**) and a CDV-infected dog (**A′**). Infection results in increased GFAP protein positivity. Scale bars = 20 µm. (**B**,**B**’) Detection of ALDH1L1 (brown) and S100 (green) in tissue of a control animal (**B**) and a CDV-infected dog (**B’**). Infected animals show loss of S100 signal. Scale bars = 20 µm. (**C**–**E**) Quantification of ALDH1L1^+^/GFAP^−^ cells (**C**), ALDH1L1^−^/GFAP^+^ cells (**D**) and ALDH1L1^+^/GFAP^+^ double-positive cells (**E**). (**F**–**H**) Quantification of ALDH1L1^+^/S100^−^ cells (**F**), ALDH1L1^−^/S100^+^ cells (**G**) and ALDH1L1^+^/S100^+^ cells double-positive cells (**H**). (**C**–**H**) Box and whisker plots display median and quartiles with maximum and minimum values. Significant differences (*p* ≤ 0.05, Mann–Whitney U-test) are labeled by asterisks.

Control animals showed an ALDH1L1^+^ signal in somata and processes of white matter astrocytes (Fig. 3). Within CDV lesions, astrocytes displayed gemistocytic morphology, but no significant changes in the number of ALDH1L1^+^ cells was found in CDV lesions (group 2, 3, 4) compared to control dogs (Fig. 4). Increased cytoplasmic GFAP protein levels were found in ALDH1L1^+^ cells of CDV-infected dogs by double labeling. Moreover, GFAP staining was found in astrocytic cells without detectable ALDH1L1 protein positivity (GFAP^+^/ALDH1L1^−^ cells) in demyelinating lesions, indicating a dominating GFAP elevation in reactive astrocytes^55^ (Fig. 5). Conclusively, unchanged numbers of intralesional ALDH1L1^+^ cells show that the increased density of GFAP^+^ cells is due to higher protein amounts and primarily not a consequence of astrocyte proliferation in CDV-induced white matter lesions.

Immunohistochemistry revealed a significant reduction of S100^+^ cells in white matter lesions of CDV-infected dogs (Fig. 2). Since S100 is not restricted to the astrocytic lineage but also expressed in oligodendrocytes and glial precursor cells^47,48^, double labeling was performed to analyse S100 protein product in ALDH1L1^+^ astrocytes during infection. While the number of ALDH1L1^−^/S100^+^ cells declined, the amount of S100^+^/ALDH1L1^+^ double labeled cells remained unchanged. Data indicate that the S100 decline in infected dogs is due to depletion of resident glial cells other than astrocytes within demyelinating lesions (Fig. 5).

AQP4 was found in the white matter neuropil of all control animals. Astrocytic foot processes presented as continuous signal around capillaries and depicted clearly the astrocytic component of the blood brain barrier (Fig. 3). In acute lesions of CDV-infection (group 2), a decrease in AQP4 protein levels was apparent. In subacute and chronic lesions (groups 3 and 4), a significant decrease of morphometric density was observed, characterized by a reduction of signal intensity in the neuropil or total loss in perivascular astrocytic foot processes, respectively (Fig. 4).

Immunohistochemistry for GS showed an intracytoplasmic signal within numerous astrocytic cells of control animals (Fig. 3). In CDV lesions of all groups (groups 2, 3, 4) the number of GS^+^ astrocytes significantly decreased compared to healthy control dogs (Fig. 4).

Immunohistochemistry for detecting the astrocyte-related anti-apoptotic protein survivin showed single immunopositive cells within control animals (group 1). A significant upregulation of survivin protein product was observed in all groups of distemper encephalitis in cells with astrocytic morphology (Figs 3 and 4). In addition to astrocytes, survivin positivity was also found in inflammatory cells, including gitter cells in advanced demyelinating lesions (groups 3 and 4).

### Astrocyte-related gene expression in demyelinating leukoencephalitis

**Figure 6:**
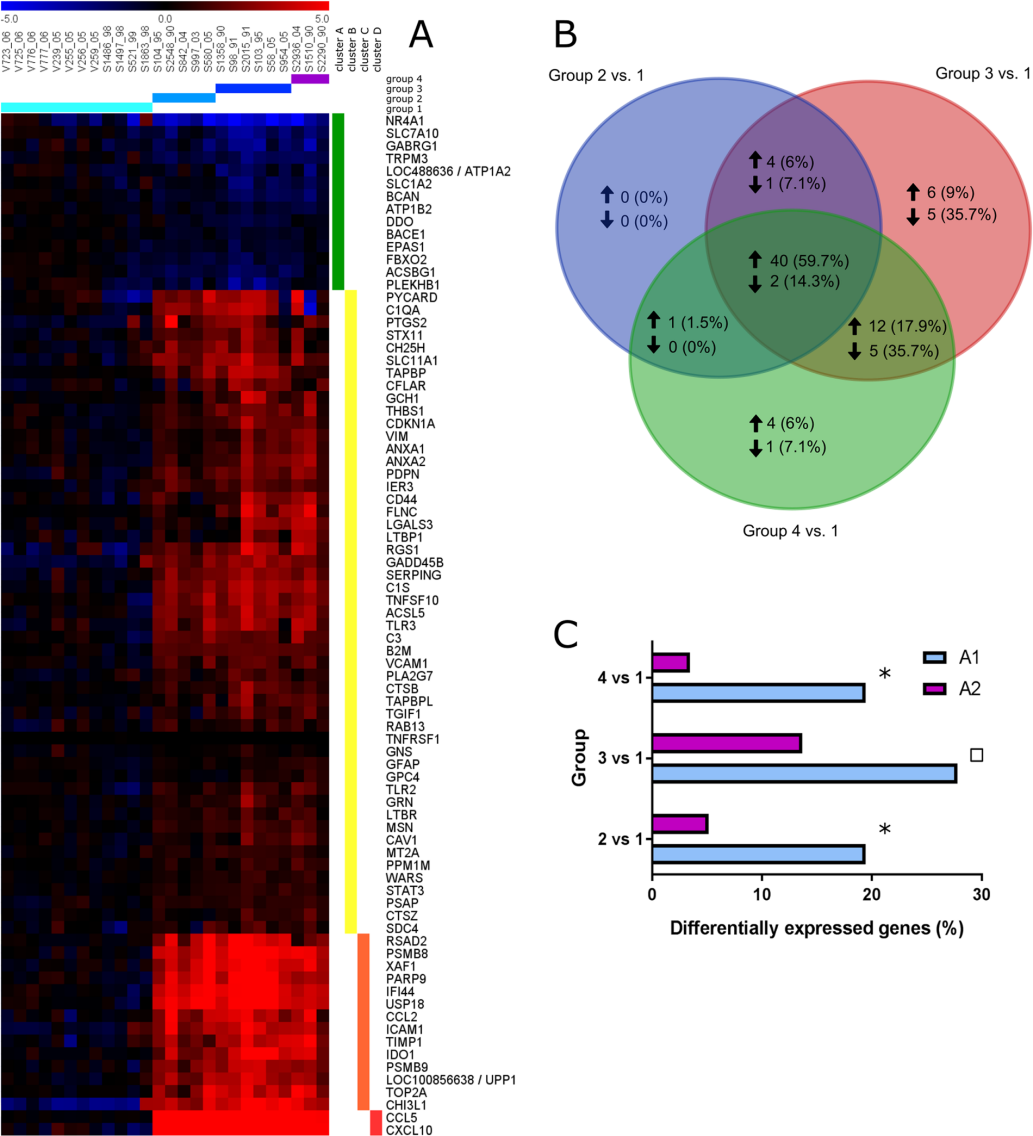
Astrocytic gene expression analyses. (**A**) Expression profile of 81 differentially expressed astrocyte-associated genes in the white matter during the course of CDV-induced demyelinating leukoencephalitis. The heat map displays log2-transformed individual fold changes relative to the mean expression of control animals indicated by a color scale ranging from −5 (relative low expression) in blue to 5 (relative high expression) in red. Each row represents one of 81 differentially expressed genes (DEGs) and each column one of the 26 biological replicates (cerebellar specimens of individual dogs) sorted according to the histologically defined groups of CDV-induced leukoencephalitis. Fold changes were grouped by hierarchical cluster analysis to reveal similar expression patterns. DEGs were subdivided into four clusters with distinct expression profiles: cluster A (green bar) contains downregulated genes, whereas cluster B (yellow bar) shows mildly upregulated genes, cluster C (orange bar) moderately upregulated and cluster D (red bar) severely upregulated genes. (**B**) Venn diagram comparing up- and downregulated astrocyte-related DEGs within the defined groups of CDV leukoencephalitis. Group 2–4 are compared for shared (intersection) and unique DEGs, depicted by total numbers and proportion (percentage). Upregulated genes are marked by an upward directed arrow, downregulated by a downward directed one. **(C)** Comparison of the relative proportion of A1- and A2-related differentially expressed genes employing the Fisher’s exact test revealed significant dominance of A1-related genes in group 2 and group 4. Significant differences (*p* ≤ 0.05) are labeled by asterisks. Statistical tendency is labeled by a square (*p* = 0.073).

In order to get insights into alterations of astrocytic gene expression during CDV-DL microarray analyses of cerebellar tissue have been performed. A total of 2184 astrocyte-related genes were extracted from peer-reviewed publications and genome databases (Supplemental Table S3). Comparison with the entire data set^32^ revealed 81 astrocyte-related genes (Supplemental Table S4) that were differentially expressed in CDV-induced leukoencephalitis. 67 genes were upregulated and 14 were downregulated. More than half (59.7%) of upregulated genes were upregulated in all three groups (groups 2–4) of CDV-DL. Among downregulated DEGs, 78.5% were either solely downregulated in subacute lesions of group 3 (35.7%) and subacute to chronic lesions of group 4 (7.1%) or in both (35.7%). Thus, downregulation was predominantly observed in late phases of CDV-induced leukoencephalitis. Comparison of DEGs within defined CDV groups is depicted in Fig. 6B. In order to detect similarities in the expression pattern of the 81 astrocyte-related DEGs, the log2 transformed fold changes of control dogs and CDV-infected dogs for each group were analysed through hierarchical cluster analysis employing Euclidean distance and visualized by a heat map (Fig. 6A). The resulting four hierarchical clusters (cluster A – D) grouped genes with similar expression. In order to assign a biological meaning to these genes, the functional annotation tool from DAVID was applied to all hierarchical clusters (https://david.ncifcrf.gov/summary.jsp). Significantly enriched gene ontology terms (GO terms, FDR < 1.0%) are listed in Table 1. From each cluster GO terms subjectively giving the best description of the whole cluster were manually chosen. Cluster A contains 14 downregulated genes. A specific GO term could not be assigned to this cluster, as the highest ranked ontology term did not meet the cut-off criteria for significant enrichment (FDR = 8.6%). Interestingly, multiple downregulated genes are involved in glutamate detoxification, such as GLT-1, SLC7A10, DDO and ATP1A2. In cluster B, mildly upregulated genes were grouped (n = 51), which were associated to gene ontology terms such as *immune system process*, *positive regulation of immune system process*, *apoptotic process* and *positive regulation of signal transduction* (FDR < 0.3). Cluster C included moderately upregulated genes (n = 14) and was enriched in the ontology term *response to cytokine*. Hierarchical cluster D grouped 2 genes, which were severely upregulated in CDV-DL. Functional annotation revealed an association of the genes with the term *regulation of T cell chemotaxis*. However, the term was slightly above the cut-off criteria (FDR = 1.1%).

**Table 1:** Significantly enriched gene ontology terms related to differentially expressed astrocyte-associated genes.

Hierarchical cluster*	Gene ontology terms	Number of genes	*FDR* [%]
A	Not assigned		
B	immune system process	22	<0.1
	positive regulation of immune system process	15	<0.1
	apoptotic process	14	0.1
	positive regulation of signal transduction	13	0.3
C	response to cytokine	5	0.3
D	Not assigned		

*Hierarchical clusters refer to the respective cluster of genes with a similar expression pattern obtained from hierarchical cluster analysis as displayed in Fig. 5A. Functional annotation was performed for each hierarchical cluster and specific gene ontology terms that subjectively gave the best description of the cluster and met the cut-off criteria of FDR ≤ 1.0% were selected.

For characterization of astrocytic A1/A2-polarization associated genes were extracted from a peer-reviewed publication (Supplemental Table S5)^27^. 36 genes were assigned to the A1-phenotype and 117 genes were expressed by the A2-phenotype. Fisher’s exact test was employed for comparison of the relative proportion of A1- and A2-genes within the CDV-DL groups (Fig. 6C). The test revealed a significantly higher percentage of differentially expressed A1-marker genes for group 2 (*p* = 0.013) and group 4 (*p* = 0.004). In addition a statistical tendency towards the A1-phenotype was observed for group 3 (*p* = 0.073). Compared to controls 19.44%, 27.78% and 19.44% of A1-related genes were differentially expressed in group 2, 3 and 4, respectively. In contrast, the percentage of A2-related DEGs accounts for 5.13%, 13.68%, and 3.42% for respective group comparison. The dominance of A1-related DEGs indicates a shift towards a neurotoxic astrocyte phenotype during CDV-induced leukoencephalitis.

**Figure 7:**
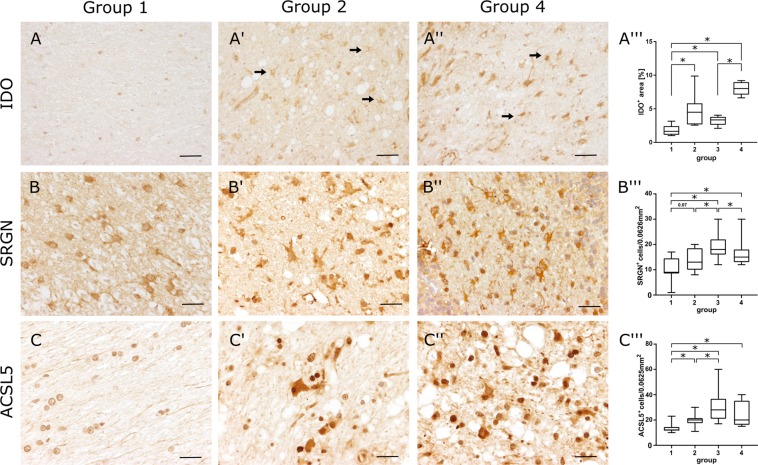
Confirmation of gene expression analysis by immunohistochemical detection of indoleamine 2,3-dioxygenase **(IDO**, **A**,**A′′′**), serglycin **(SRGN**, **B**,**B′′′)** and acyl-CoA synthetase long-chain family member 5 **(ACSL5**, **C**,**C′′′)** protein in the cerebellar white matter. **(A)** Control tissue with few small immunopositive cells in the healthy white matter. (**A′**) Numerous IDO^+^ cells showing astrocyte morphology (arrows) within an acute lesion (group 2). (**A′′**) Inflammatory lesion (group 4) with even higher density of IDO^+^ cells with astrocyte morphology (arrows). **(B)** Control tissue with SRGN^+^ astrocytes. (**B′**) Acute (group 2) and (**B′′**) subacute to chronic lesions (group 4) with increased numbers of SRGN^+^ cells. **(C)** Control tissue with few ACSL^+^ cells. **(C′)** Increased numbers of ACSL^+^ cells with gemistocytic morphology in acute (group 2) and (**C′′**) subacute to chronic lesions (group 4). Scale bars = 50 µm (**A′′′**–**C′′′**) Statistical analysis shows significant increase of IDO^+^ area (**A′′′**) and significantly increased numbers of SRGN^+^ cells (**B′′′**) and ACSL5^+^ cells (**C′′′**) in infected dogs. Box and whisker plots display median and quartiles with maximum and minimum values. Significant differences (*p* ≤ 0.05, Mann–Whitney U-test) are labeled by asterisks.

The immune modulating enzyme IDO was upregulated in all CDV groups in microarray analysis. Immunohistochemistry confirmed upregulation of IDO mainly in cells displaying astrocyte morphology throughout the cerebellar white matter. Some of these cells exhibited a reactive phenotype (gemistocytes). Densitometry revealed an increased IDO^+^ area in all groups of CDV lesions with highest values in chronic lesions of group 4 (Fig. 7).

**Figure 8:**
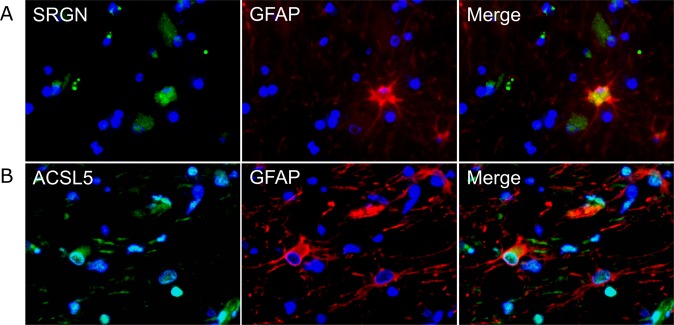
Immunofluorescence double labeling for detecting SRGN (**A**) and ACSL5 (**B**) protein in GFAP^+^ astrocytes in demyelinating lesions. SRGN^+^ and ACSL5^+^ cells (green) and GFAP^+^ cells (red) show co-localization (yellow). Blue: nuclear counterstaining.

SRGN and ACSL5, representing markers for the neurotoxic A1-phenotype of astrocytes^28^, were upregulated in CDV-infected animals in microarray analysis and in immunohistochemistry (Fig. 7). SRGN was increased in infected lesions of all groups. ACSL5^+^ cells was significantly increased in group 3 and 4 lesions. In group 2 lesions, a statistical trend (p = 0.07) of an ACSL5 increase was found (Fig. 7). Most of SRGN^+^ and ACSL5^+^ cells exhibited astrocyte morphology. In addition also some infiltrating leukocytic cells and microglia displayed SRGN and ACSL5 positivity, respectively. Immunofluorescence double labeling with GFAP revealed co-localization with SRGN (GFAP^+^/SRGN^+^ cells) and ACSL5 (GFAP^+^/ACSL5^+^ cells) in CDV lesions characteristic of A1-astrocytes (Fig. 8).

## Discussion

Astrocytes play a central role in maintaining normal CNS physiology and critically control the response to brain injury and neurological diseases. Phenotypical and molecular analyses of the present study revealed an altered metabolism and neurotoxic properties of reactive astrocytes in CDV lesions, which have the potential to disturb neurotransmitter uptake and blood brain barrier homeostasis.

Reactive astrocyte responses, characterized by increased GFAP protein levels and mRNA expression, were found already before the onset of overt demyelination in acute brain lesion of CDV-infected dogs. Similarly, reactive astrocytes are present in the normal-appearing white matter of MS patients and in pre-demyelinating lesions in the EAE model, suggesting an early contribution of glial cells to lesion development by chemokine release and initiation of inflammatory responses^56–59^. As observed in astrocytes within developing MS lesions, gene expression analyses revealed an upregulation of pro-inflammatory cytokines such as CCL5 and CXCL10 in the early phase of CDV-DL^57^. Moreover, several other genes associated with activation of immune response and complement cascade, such as TLR2, TLR3, CCL2, CD44, VCAM1, C1QA, C1S, C3 and STAT3, were upregulated^32^. The activation of the STAT3 pathway by interleukin-6 results in astrogliosis in demyelinating lesions of Theiler’s murine encephalomyelitis (TME)^60^. The presented findings indicate that reactive astrocytes contribute to the inflammatory process in CDV-induced leukoencephalitis by pro-inflammatory stimuli.

Immunohistochemistry showed a significant loss of AQP4 together with a GFAP increase in acute lesions and GFAP retention in chronic lesions, respectively. AQP4, a water channel exclusively expressed on astrocytes in the brain, is located at astrocytic end feet surrounding blood vessels and thus regulates water homeostasis at the blood brain barrier. It enables fast water influx or efflux and facilitates reabsorption of excess fluid in vasogenic brain edema^61,62^. Indicating an early blood brain barrier dysfunction, AQP4 reduction was observed before the onset of overt demyelination in CDV-infected dogs. Dysfunction and loss of AQP4 plays a crucial role in development of brain edema and was shown to cause prolonged seizure duration through impaired K^+^ buffering in AQP4^−/−^ mice^63^. Moreover, loss of AQP4 is a hallmark of active demyelinating lesions in *neuromyelitis optica* (NMO) in human patients^64^.

Immunophenotyping revealed decreased protein levels of the astrocyte-specific enzyme GS in acute lesions and foci of progressive myelin loss in CDV-DL. GS catalyzes the rapid degradation of glutamate to non-neurotoxic amino acid glutamine, thereby preventing neurodegenerative processes^65,66^. In several pathological conditions, reduction of GS activity has been detected, for example schizophrenia^67^, Alzheimer’s disease^68^, epilepsy^69,70^, hypoxia^71^, diabetes^72^ and MS^24^ as well as in the EAE model^73^. Therefore, GS decline indicates disturbed homeostasis of the glutamatergic system in CDV-DL and highlights the importance of glutamate toxicity in demyelination following morbillivirus infection. In accordance with this, transcriptional analysis of the present study of astrocyte-related DEGs revealed significant downregulation of genes involved in glutamate detoxification, such as GLT-1 (=SLC1A2), SLC7A10, D-aspartate oxidase (DDO) and ATP1A2. The excitatory amino acid transporter GLT-1 accounts for the majority (90%) of glutamate uptake in the CNS. Decreased transport activity contributes to impaired glutamate uptake and raised extracellular glutamate concentration^74^. Dysfunction of GS and reduced astrocytic GLT-1 protein leading to glutamate excitotoxicity can be observed in EAE^74,75^ and in active MS lesions^76^. Interestingly, internalization and redistribution of AQP4 in NMO is also accompanied by downregulation of its physically associated glutamate transporter GLT-1^77^. Subsequent reduced glutamate uptake is supposed to induce glutamate toxicity to myelin-producing oligodendrocytes^78^. Gene expression analysis revealed an upregulation of the neuronal glutamate transporter SLC1A1 in the chronic phase of CDV-DL^32^. In agreement with this finding, previous studies revealed an upregulation of SLC1A1 in the hippocampus of CDV-infected dogs showing seizures compared to those without epileptic seizures, which is supposed to be a compensatory neuronal mechanism following elevated extracellular glutamate levels to prevent excitotoxicity^79^.

Astrocytic SLC7A10 is an amino acid transporter mediating transport of the NMDA receptor co-agonists glycine and D-serine in the CNS and thus functions as regulator of NMDA receptor activity at glutamatergic synapses^80^. Binding of co-agonists to this glutamate receptor increases the affinity to glutamate. Thus, decreased clearance of glycine and D-serine by SLC7A10 causes overstimulation of NMDA receptors and thereby contributes to glutamate excitotoxicity^81–83^. Mice lacking this transporter develop tremors, ataxia and seizures in consequence to neuronal hyperexcitability^84,85^.

Similarly to GS, DDO selectively degrades D-aspartate, which is also known to be an agonist of the NMDA receptor. *DDO*-*knockout* mice show substantially increased extracellular glutamate levels in the CNS^86^. Downregulation of the sodium-potassium-ATPase ATP1A2, as observed by gene expression analysis of the present study, leads to breakdown of the electrochemical gradient, which is a prerequisite for neuronal excitability and activity of glutamate transporters, such as GLAST and GLT-1. Thus, decreased expression of ATP1A2 leads secondarily to increased extracellular amounts of glutamate and likewise contributes to glutamate excitotoxicity^87^. Consequently, findings indicate disturbed astrocytic glutamate homeostasis, which potentially causes excitotoxic effects. Besides neurons, oligodendrocytes are particularly vulnerable to glutamate toxicity^23,88–90^. Thus, damage of myelinating oligodendrocytes by glutamate excess might contribute to the initiation and progression in CDV-induced leukoencephalitis, as described in the EAE model and human MS^91,92^.

Regarding A1/A2-polarization of astrocytes, gene expression analysis was performed and confirmed by ACSL5- and SRGN-specific immunohistochemistry and immunofluorescence. Findings strongly indicate astrocytic polarization towards a neurotoxic A1-phenotype. A1-astrocytes are present in different neurodegenerative diseases, such as MS and Alzheimer’s disease, and show decreased phagocytic capacity, which leads to disturbed clearance of myelin debris. In contrast to neuroprotective A2-astrocytes, A1-astrocytes lose their neurotrophic function, promote pro-inflammatory responses, trigger neurotoxicity, and thereby contribute to neuronal and oligodendroglial death^28^. The observation of an imbalanced astrocyte polarization towards the neurotoxic A1-phenotype further supports the notion of maladaptive astrogliosis in CDV-DL. GO-annotation of astrocyte-related DEGs showed upregulation of several genes assigned to the apoptotic process. The present study showed over-expression of the astrocyte-related, anti-apoptotic protein survivin, that is a feature of active MS lesions^93^. In TME, survivin prevents apoptosis of infected astrocytes, favoring viral persistence^94,95^. Moreover, astrocyte apoptosis resistance was demonstrated in TME associated with glial scarring and chronic demyelination^94,96^. Lack of apoptosis has been shown also in primary astrocyte cultures infected with CDV, which is supposed to support cell-to-cell transmission of the virus in the brain of infected dogs^8,97^. Similarly, non-cytolytic spread is a putative prerequisite for persistent measles virus infection and subacute sclerosing panencephalitis in human patients^97^.

Within gene expression analyses, IDO was upregulated in all phases of CDV-DL. Immunohistochemistry confirmed an intralesional increase of IDO protein within astrocytic cells. The enzyme catalyzes the first step in the degradation of tryptophan through the kynurenine pathway^98^. In the brain, IDO is expressed by astrocytes, neurons and microglia^99,100^. It exerts immune modulating functions and suppresses replication and spread of infectious agents by deprivation of tryptophan, as demonstrated in measles virus infection^101–107^. However, tryptophan degradation through the kynurenine pathway leads to metabolites such as 3-hydroxykynurenine and quinolinic acid, which show neurotoxic effects in the CNS through production of reactive radical species and activation of glutamate receptors^108–110^. Thus, similar to human measles, ambivalent functions of IDO with beneficial effects by supporting antiviral immunity and reducing immunopathology and detrimental neurotoxic effects contributing to neuronal and oligodendroglial damage in CDV-DL have to be considered^106,111^.

The present study provides a comprehensive database of astrocyte-related gene expression during the initiation and progression of CDV-DL. Reactive astrocytes in canine distemper show neurotoxic properties, which have the potential to cause neurodegeneration, demyelination, and impaired remyelination. There is cumulative evidence that astrocytopathies with disturbed astrocyte function and maladaptive astrogliosis are crucial factors in the pathogenesis of different inflammatory neurological diseases^112^. Thus, understanding the complex nature of astrocyte plasticity in CDV-DL represents a prerequisite for targeted therapeutic strategies in canine CNS disorders.

## Supplementary information

Supplemental tables

## Data Availability

MIAME compliant data sets are deposited in the ArrayExpress database (accession number: E-MEXP-3917; http://www.ebi.ac.uk/arrayexpress). All other datasets generated and analysed during the current study are available from the corresponding author on reasonable request.
