# Supplementary material for: Neurotoxic potential of reactive astrocytes in canine distemper demyelinating leukoencephalitis
Source: Sci Rep. 2019 Aug 12;9:11689. doi: 10.1038/s41598-019-48146-9 (PMC6690900; doi:10.1038/s41598-019-48146-9)
Supplement: Supplementary file 1 — Supplemental tables [file 41598_2019_48146_MOESM1_ESM.docx]

**Neurotoxic potential of reactive astrocytes in canine distemper demyelinating leukoencephalitis**

J. Klemens^1^, M. Ciurkiewicz^1,3^, E. Chludzinski^1,3^, M. Iseringhausen^1^, D. Klotz^1^, V.M. Pfankuche^1,3^, R. Ulrich^2,3^, V. Herder^1,3^, C. Puff^1^, W. Baumgärtner^1,3^, A. Beineke^1,3,*^

^1^Department of Pathology, University of Veterinary Medicine Hanover, Hannover, Germany;

^2^Department of Experimental Animal Facilities and Biorisk Management, Friedrich-Loeffler-Institut, Greifswald, Insel Riems, Germany;

^3^Center for Systems Neuroscience, Hannover, Germany

^*^Corresponding author

# Supplemental tables

# Supplemental table S1: Anamnestic details of control dogs and canine distemper virus-infected dogs

|  | | | | |
| --- | --- | --- | --- | --- |
|  | **Animal no.** | **Age**  **(month)** | **Sex** | **Breed** |
| Control dogs | 1 | 4.5 | n.d. | Beagle |
|  | 2 | 4.5 | n.d. | Beagle |
|  | 3 | 4.5 | n.d. | Beagle |
|  | 4 | 4.5 | n.d. | Beagle |
|  | 5 | 4.5 | n.d. | Beagle |
| Canine distemper virus- infected dogs | 6 | 36 | m | Mixed breed |
|  | 7 | 4 | f | Dachshund |
|  | 8 | 24 | f | Mixed breed |
|  | 9 | 5 | f | German Spaniel |
|  | 10 | n.d. | m | German Shepherd |
|  | 11 | 10 | m | German Shepherd |
|  | 12 | 8 | f | Retriever |
|  | 13 | n.d. | n.d. | n.d. |
|  | 14 | 3 | f | Dachshund |
|  | 15 | 6 | sf | Mixed breed |
|  | 16 | 72 | m | Mixed breed |
|  | 17 | n.d. | f | n.d. |
|  | 18 | n.d. | n.d. | n.d. |
|  | 19 | 2 | f | Labrador Retriever |
|  | 20 | 2 | m | Mixed breed |
|  | 21 | 84 | m | Miniature pinscher |
|  | 22 | 7 | f | Husky |
|  | 23 | 6 | m | Mixed breed |
|  | 24 | 3 | m | Shih Tzu |
|  | 25 | 3,5 | f | Jack Russel Terrier |
|  | 26 | 5 | f | Chihuahua |
|  | 27 | 12 | f | Mixed breed |
|  | 28 | 108 | f | Boxer |
|  | 29 | n.d. | n.d. | n.d. |
|  | 30 | n.d. | n.d. | n.d. |
|  | 31 | n.d. | n.d. | n.d. |
|  | 32 | 4 | f | Mixed breed |
|  | 33 | 3 | m | Mixed breed |
|  | 34 | 48 | m | Mixed breed |
| n.d. = no data; m = male; f = female; sf = spayed female | | | | |

# Supplemental table S2: Details for primary antibodies used for immunohistochemistry and immunofluorescence

|  | | | | | |
| --- | --- | --- | --- | --- | --- |
| **Antigen** | Supplier | **Catalogue or clone no.** | **Clonality** | **Pretreatment** | **Dilution** |
| **CDV-NP** | C. Örvell,  Stockholm, Sweden | Clone 3991 | Monoclonal mouse | Microwave treatment/ CB | 1:6000* |
| **MBP** | Merck Millipore, Billerica, USA | AB 980 | Polyclonal rabbit | none | 1:800* |
| **Nogo-A** | Merck Millipore, Billerica, USA | AB 5664 P | Polyclonal rabbit | Microwave treatment/ CB | 1:500* |
| **ACSL5** | Aviva Systems Biology,  USA | ARP47232_P050 | Polyclonal rabbit | Microwave treatment/ CB | 1:50*  1:20** |
| **ALDH1L1** | Abcam,  Cambridge, UK | AB 177463 | Monoclonal rabbit | Microwave treatment/ CB | 1:50* |
| **ALDH1L1** | Novus Biologicals,  USA | OTI3D2 | Monoclonal mouse | Microwave treatment/ CB | 1:100* |
| **AQP4** | Merck Millipore, Billerica, USA | AB 3594 | Polyclonal rabbit | none | 1:500* |
| **GFAP** | DakoCytomation GmbH, Hamburg, Germany | A0334 | Polyclonal rabbit | none | 1:2000* |
| **GFAP** | Abcam,  Cambridge, UK | AB 53554 | Polyclonal goat | none | 1:200** |
| **GS** | Santa Cruz Biotechnology, Inc., Heidelberg, Germany | SC-9067 | Polyclonal rabbit | Microwave treatment/ CB | 1:50* |
| **IDO** | LifeSpan BioSciences, Seattle, USA | OTI2G4 | Monoclonal mouse | Microwave treatment/ CB | 1:200* |
| **SRGN** | Elabscience,  USA | E-AB-15409 | Polyclonal rabbit | Microwave treatment/ CB | 1:50*  1:20** |
| **Survivin** | Novus Biologicals, Cambridge, UK | NB 500-201 | Polyclonal rabbit | Microwave treatment/ CB | 1:1000* |
| **S100** | DakoCytomation GmbH, Hamburg, Germany | Z0311 | Polyclonal rabbit | Microwave treatment/ CB | 1:800* |
| ACSL5 = acyl-coA synthetase long-chain family member 5; ALDH1L1 = aldehyde dehydrogenase 1L1; AQP4 = Aquaporin 4; CB = citrate buffer; CDV-NP = canine distemper virus- nucleoprotein; GFAP = glial fibrillary acidic protein; GS = glutamine synthetase; IDO = indoleamine 2,3-dioxygenase; MBP = myelin basic protein; no. = number; SRGN = serglycin: * = dilution used for immunohistochemistry; ** = dilution used for immunofluorescence | | | | | |

# Supplemental table S3: Astrocyte-related genes

| **Gene symbol** | **Gene title** | **Entrez gene ID** |
| --- | --- | --- |
| 1110020G09RIK | NAD kinase 2, mitochondrial | 612569 |
| 1200009O22RIK | TLR4 interactor with leucine rich repeats | 482378 |
| A2M | alpha-2-macroglobulin | 477699 |
| AAK1 | AP2 associated kinase 1 | 474625 |
| AASS | aminoadipate-semialdehyde synthase | 482429 |
| ABAT | 4-aminobutyrate aminotransferase | 479856 |
| ABCA1 | ATP-binding cassette, sub-family A (ABC1), member 1 | 481651 |
| ABCA5 | ATP-binding cassette, sub-family A (ABC1), member 5 | 480455 |
| ABCB9 | ATP-binding cassette, sub-family B (MDR/TAP), member 9 | 477456 |
| ABCC4 | ATP-binding cassette, sub-family C (CFTR/MRP), member 4 | 485523 |
| ABCD2 | ATP-binding cassette, sub-family D (ALD), member 2 | 477643 |
| ABCD3 | ATP-binding cassette, sub-family D (ALD), member 3 | 479939 |
| ABCD4 | ATP-binding cassette, sub-family D (ALD), member 4 | 490781 |
| ABCF1 | ATP binding cassette subfamily F member 1 | 474826 |
| ABHD10 | abhydrolase domain containing 10 | 478561 |
| ABHD11 | abhydrolase domain containing 11 | 489803 |
| ABHD12 | abhydrolase domain containing 12 | 477004 |
| ABHD14B | abhydrolase domain containing 14B | 484744 |
| ABHD3 | abhydrolase domain containing 3 | 480177 |
| ABHD4 | abhydrolase domain containing 4 | 607421 |
| ABHD6 | abhydrolase domain containing 6 | 484712 |
| ABHD8 | abhydrolase domain containing 8 | 484840 |
| ABI1 | abl-interactor 1 | 607247 |
| ABI2 | abl interactor 2 | 488485 |
| ABR | active BCR-related gene | 480638 |
| ABTB2 | ankyrin repeat and BTB domain containing 2 | 483430 |
| ACAD11 | acyl-CoA dehydrogenase family member 11 | 100856488 |
| ACAD8 | acyl-Coenzyme A dehydrogenase family, member 8 | 479386 |
| ACADL | acyl-Coenzyme A dehydrogenase, long chain | 478895 |
| ACADM | acyl-Coenzyme A dehydrogenase, C-4 to C-12 straight chain | 490207 |
| ACADSB | acyl-Coenzyme A dehydrogenase, short/branched chain | 477856 |
| ACADVL | acyl-Coenzyme A dehydrogenase, very long chain | 489463 |
| ACBD5 | acyl-Coenzyme A binding domain containing 5 | 477987 |
| ACO1 | aconitase 1, soluble | 481576 |
| ACOT11 | acyl-CoA thioesterase 11 | 489576 |
| ACOT2 | acyl-CoA thioesterase 2 | 490770 |
| ACOX1 | acyl-Coenzyme A oxidase 1, palmitoyl | 483322 |
| ACP2 | acid phosphatase 2, lysosomal | 475983 |
| ACP6 | acid phosphatase 6, lysophosphatidic | 475822 |
| ACSBG1 | acyl-CoA synthetase bubblegum family member 1 | 479067 |
| ACSL3 | acyl-CoA synthetase long-chain family member 3 | 478927 |
| ACSL5 | acyl-CoA synthetase long-chain family member 5 | 477820 |
| ACSL6 | acyl-CoA synthetase long-chain family member 6 | 474670 |
| ACSS1 | acyl-CoA synthetase short-chain family member 1 | 477002 |
| ACSS2 | acyl-CoA synthetase short-chain family member 2 | 477205 |
| ACTA2 | gamma 2, smooth muscle, enteric | 478250 |
| ACTB | actin beta | 403580 |
| ACTN1 | actinin alpha 1 | 480369 |
| ACTR3B | ARP3 actin related protein 3 homolog B | 475549 |
| ACYP2 | acylphosphatase 2, muscle type | 474595 |
| ADAM12 | ADAM metallopeptidase domain 12 | 100686018 |
| ADAM17 | ADAM metallopeptidase domain 17 | 475662 |
| ADAMTS4 | ADAM metallopeptidase with thrombospondin type 1 motif 4 | 488651 |
| ADAMTS5 | ADAM metallopeptidase with thrombospondin type 1 motif, 5 | 487713 |
| ADAMTS6 | ADAM metallopeptidase with thrombospondin type 1 motif, 6 | 478077 |
| ADAMTS9 | ADAM metallopeptidase with thrombospondin type 1 motif, 9 | 606847 |
| ADCK4 | aarF domain containing kinase 4 | 484498 |
| ADCY1 | adenylate cyclase 1 | 607357 |
| ADCY8 | adenylate cyclase 8 (brain) | 482045 |
| ADCYAP1R1 | adenylate cyclase activating polypeptide 1 (pituitary) receptor type I | 482386 |
| ADD3 | adducin 3 (gamma) | 486881 |
| ADGRG1 | adhesion G protein-coupled receptor G1 | 487261 |
| ADHFE1 | alcohol dehydrogenase, iron containing, 1 | 477899 |
| ADK | adenosine kinase | 479253 |
| ADORA2B | adenosine A2B receptor | 403410 |
| ADRA1A | adrenergic, alpha-1A-, receptor | 403866 |
| ADRA2A | adrenergic, alpha-2A-, receptor | 486888 |
| ADRB1 | adrenergic, beta-1-, receptor | 493972 |
| ADRBK2 | adrenergic, beta, receptor kinase 2 | 486327 |
| AGA | aspartylglucosaminidase | 475638 |
| AGL | amylo-1, 6-glucosidase, 4-alpha-glucanotransferase | 479931 |
| AGPAT5 | 1-acylglycerol-3-phosphate O-acyltransferase 5 | 100684122 |
| AGT | angiotensinogen | 403783 |
| AGTRAP | angiotensin II receptor-associated protein | 608333 |
| AGXT2L1 | ethanolamine-phosphate phospho-lyase | 478511 |
| AHCYL1 | adenosylhomocysteinase-like 1 | 611790 |
| AHNAK | AHNAK nucleoprotein | 476059 |
| AHNAK2 | AHNAK nucleoprotein 2 | 612700 |
| AHR | aryl hydrocarbon receptor | 475251 |
| AIFM1 | apoptosis-inducing factor, mitochondrion-associated, 1 | 481048 |
| AIFM2 | apoptosis-inducing factor, mitochondrion-associated, 2 | 479236 |
| AK2 | adenylate kinase 2 | 478145 |
| AK3 | adenylate kinase 3 | 476342 |
| AKAP12 | A-kinase anchoring protein 12 | 476246 |
| AKT2 | v-akt murine thymoma viral oncogene homolog 2 | 449021 |
| ALAS1 | aminolevulinate, delta-, synthase 1 | 476600 |
| ALCAM | activated leukocyte cell adhesion molecule | 478550 |
| ALDH16A1 | aldehyde dehydrogenase 16 family, member A1 | 610267 |
| ALDH1A1 | aldehyde dehydrogenase 1 family, member A1 | 476323 |
| ALDH1L1 | Aldehyde dehydrogenase 1, family member L1 | 100855730  476506 |
| ALDH2 | aldehyde dehydrogenase 2 family member | 610941 |
| ALDH4A1 | aldehyde dehydrogenase 4 family, member A1 | 612452 |
| ALDH5A1 | aldehyde dehydrogenase 5 family, member A1 | 488246 |
| ALDH6A1 | aldehyde dehydrogenase 6 family, member A1 | 490779 |
| ALDH7A1 | aldehyde dehydrogenase 7 family, member A1 | 481486 |
| ALDOC | aldolase C, fructose-bisphosphate | 480622 |
| ALG3 | alpha-1,3- mannosyltransferase | 478653 |
| ALKBH7 | alkB, alkylation repair homolog 7 (E. coli) | 611351 |
| ALS2CL | ALS2 C-terminal like | 484786 |
| AMHR2 | anti-Mullerian hormone receptor, type II | 486506 |
| AMIGO1 | adhesion molecule with Ig-like domain 1 | 490120 |
| AMIGO2 | adhesion molecule with Ig-like domain 2 | 486594 |
| AMMECR1L | AMME chromosomal region gene 1-like | 483862 |
| AMOT | similar to angiomotin | 481022 |
| AMOTL1 | angiomotin like 1 | 485122 |
| AMPD2 | adenosine monophosphate deaminase 2 (isoform L) | 479913 |
| AMPD3 | adenosine monophosphate deaminase (isoform E) | 476851 |
| ANAPC10 | anaphase promoting complex subunit 10 | 475453 |
| ANGPT1 | angiopoietin 1 | 403656 |
| ANGPTL4 | angiopoietin-like 4 | 476724 |
| ANK2 | similar to ankyrin 2 isoform 2 | 487908 |
| ANKRD29 | ankyrin repeat domain 29 | 606927 |
| ANKRD40 | ankyrin repeat domain 40 | 491085 |
| ANKRD57 | ankyrin repeat domain 57 | 481302 |
| ANKRD6 | ankyrin repeat domain 6 | 481919 |
| ANKS3 | ankyrin repeat and sterile alpha motif domain containing 3 | 490024 |
| ANP32E | acidic (leucine-rich) nuclear phosphoprotein 32 family, member E | 475834 |
| ANTXR1 | anthrax toxin receptor 1 | 612601 |
| ANTXR2 | anthrax toxin receptor 2 | 487821 |
| ANXA1 | annexin A1 | 476322 |
| ANXA2 | annexin A2 | 403435 |
| ANXA3 | annexin A3 | 478447 |
| ANXA5 | annexin A5 | 476094 |
| ANXA6 | annexin A6 | 479325 |
| ANXA7 | annexin A7 | 479246 |
| AOX1 | aldehyde oxidase 1 pseudogene | 608820 |
| AP1S2 | adaptor related protein complex 1 subunit sigma 2 | 611468 |
| APBA2 | amyloid beta precursor protein binding family A member 2 | 488700 |
| APCDD1 | adenomatosis polyposis coli down-regulated 1 | 480209 |
| APH1A | anterior pharynx defective 1 homolog A (C. elegans) | 483181 |
| APH1B | anterior pharynx defective 1 homolog B (C. elegans) | 487589 |
| APLN | apelin | 611497 |
| APOC3 | apolipoprotein C-III | 442970 |
| APOE | apolipoprotein E | 476438 |
| APPL2 | adaptor protein, phosphotyrosine interacting with PH domain and leucine zipper 2 | 481297 |
| AQP11 | aquaporin 11 | 476798 |
| AQP4 | Aquaporin 4 | 612628 |
| AQP9 | Aquaporin 9 | 487576 |
| AQR | aquarius intron-binding spliceosomal factor | 478251 |
| ARHGAP11A | Rho GTPase activating protein 11A | 487476 |
| ARHGAP12 | Rho GTPase activating protein 12 | 487088 |
| ARHGAP21 | Rho GTPase activating protein 21 | 100687590 |
| ARHGAP26 | Rho GTPase activating protein 26 | 478046 |
| ARHGAP5 | Rho GTPase activating protein 5 | 490642 |
| ARHGEF12 | Rho guanine nucleotide exchange factor (GEF) 12 | 479409 |
| ARHGEF17 | Rho guanine nucleotide exchange factor (GEF) 17 | 485201 |
| ARHGEF19 | Rho guanine nucleotide exchange factor (GEF) 19 | 487420 |
| ARHGEF4 | Rho guanine nucleotide exchange factor (GEF) 4 | 483856 |
| ARID5A | AT rich interactive domain 5A (MRF1-like) | 483050 |
| ARL6IP6 | ADP-ribosylation-like factor 6 interacting protein 6 | 609760 |
| ARMC8 | armadillo repeat containing 8 | 485684 |
| ARMC9 | armadillo repeat containing 9 | 477407 |
| ARNTL | aryl hydrocarbon receptor nuclear translocator-like | 476860 |
| ARRB1 | arrestin, beta 1 | 485189 |
| ARRDC3 | arrestin domain containing 3 | 488912 |
| ARSA | arylsulfatase A | 474457 |
| ARSK | arylsulfatase family, member K | 488903 |
| ART3 | ADP-ribosyltransferase 3 | 478434 |
| ARX | aristaless related homeobox | 608251 |
| AS3MT | arsenic (+3 oxidation state) methyltransferase | 486865 |
| ASAH1 | N-acylsphingosine amidohydrolase (acid ceramidase) 1 | 482897 |
| ASB13 | ankyrin repeat and SOCS box-containing 13 | 608587 |
| ASB6 | ankyrin repeat and SOCS box-containing 6 | 491299 |
| ASB8 | ankyrin repeat and SOCS box-containing 8 | 486587 |
| ASF1A | anti-silencing function 1A histone chaperone | 476273 |
| ASH1L | ASH1 like histone lysine methyltransferase | 480128 |
| ASNS | asparagine synthetase (glutamine-hydrolyzing) | 475240 |
| ASPG | asparaginase | 612664 |
| ASPH | aspartate beta-hydroxylase | 403846 |
| ASPM | abnormal spindle microtubule assembly | 480009 |
| ASRGL1 | asparaginase like 1 | 483789 |
| ASTN1 | astrotactin 1 | 490331 |
| ATAD2 | ATPase family, AAA domain containing 2 | 475090 |
| ATE1 | arginyltransferase 1 | 486919 |
| ATF1 | activating transcription factor 1 | 486545 |
| ATF3 | activating transcription factor 3 | 612911 |
| ATG7 | autophagy related 7 | 476533 |
| ATHL1 | acid trehalase-like protein 1 | 483395 |
| ATP13A4 | ATPase 13A4 | 100855915 |
| ATP1A2 | ATPase, Na+/K+ transporting, alpha 2 (+) polypeptide | 488636 |
| ATP1B2 | ATPase, Na+/K+ transporting, beta 2 polypeptide | 489479 |
| ATP2A2 | ATPase sarcoplasmic/endoplasmic reticulum Ca2+ transporting 2 | 403878 |
| ATP5A1 | ATP synthase F1 subunit alpha | 480149 |
| ATP5C1 | ATP synthase, H+ transporting, mitochondrial F1 complex, gamma polypeptide 1 | 478009 |
| ATP6V0A1 | ATPase H+ transporting V0 subunit a1 | 607705 |
| AURKB | aurora kinase B | 479492 |
| AXIN2 | axin 2 | 490903 |
| AXL | AXL receptor tyrosine kinase | 484490 |
| AZIN1 | antizyme inhibitor 1 | 475058 |
| B2M | beta-2-microglobulin | 100855741 |
| B3GALT1 | UDP-Gal:betaGlcNAc beta 1,3-galactosyltransferase, polypeptide 1 | 488386 |
| B3GNT5 | UDP-GlcNAc:betaGal beta-1,3-N-acetylglucosaminyltransferase 5 | 102154559 |
| B4GALT1 | UDP-Gal:betaGlcNAc beta 1,4- galactosyltransferase, polypeptide 1 | 481579 |
| B4GALT4 | UDP-Gal:betaGlcNAc beta 1,4- galactosyltransferase, polypeptide 4 | 608143 |
| B4GALT7 | beta-1,4-galactosyltransferase 7 | 481445 |
| BAALC | brain and acute leukemia, cytoplasmic | 612780 |
| BACE1 | beta-site APP-cleaving enzyme 1 | 489390 |
| BAG3 | BCL2-associated athanogene 3 | 486916 |
| BAI2 | brain-specific angiogenesis inhibitor 2 | 487317 |
| BAI3 | brain-specific angiogenesis inhibitor 3 | 481870 |
| BAMBI | BMP and activin membrane bound inhibitor | 487081 |
| BBOX1 | gamma-butyrobetaine hydroxylase 1 | 476894 |
| BBS2 | Bardet-Biedl syndrome 2 | 478121 |
| BCAN | brevican | 612102 |
| BCAP29 | B-cell receptor-associated protein 29 | 475884 |
| BCAR3 | breast cancer anti-estrogen resistance 3 | 479941 |
| BCAS1 | breast carcinoma amplified sequence 1 | 611661 |
| BCAS3 | BCAS3, microtubule associated cell migration factor | 480587 |
| BCAT1 | branched chain amino acid transaminase 1 | 486633 |
| BCKDHA | branched chain keto acid dehydrogenase E1, alpha polypeptide | 484488 |
| BCKDHB | branched chain keto acid dehydrogenase E1, beta polypeptide | 474978 |
| BCL10 | B-cell CLL/lymphoma 10 | 490183 |
| BCL2 | B-cell CLL/lymphoma 2 | 403416 |
| BCL2L13 | BCL2-like 13 | 477742 |
| BCL3 | B-cell CLL/lymphoma 3 | 612349 |
| BCL6 | B-cell CLL/lymphoma 6 | 488124 |
| BCL9L | B-cell CLL/lymphoma 9-like | 489377 |
| BCR | breakpoint cluster region | 607482 |
| BDKRB2 | bradykinin receptor B2 | 403658 |
| BDNF | brain derived neurotrophic factor | 403461 |
| BFAR | bifunctional apoptosis regulator | 489998 |
| BICC1 | BicC family RNA binding protein 1 | 608626 |
| BICD1 | BICD cargo adaptor 1 | 610028 |
| BIN1 | bridging integrator 1 | 483870 |
| BIRC5 | baculoviral IAP repeat containing 5 | 442936 |
| BLOC1S1 | biogenesis of lysosomal organelles complex-1, subunit 1 | 481098 |
| BLVRB | biliverdin reductase B (flavin reductase (NADPH)) | 476456 |
| BMP2 | bone morphogenetic protein 2 | 477162 |
| BMP2K | BMP2 inducible kinase | 487819 |
| BMP4 | bone morphogenetic protein 4 | 490695 |
| BMP7 | bone morphogenetic protein 7 | 477270 |
| BMPER | BMP binding endothelial regulator | 475280 |
| BMPR1A | bone morphogenetic protein receptor, type IA | 489077 |
| BMPR1B | bone morphogenetic protein receptor, type IB | 478484 |
| BOC | BOC cell adhesion associated, oncogene regulated | 487979 |
| BPHL | biphenyl hydrolase-like (serine hydrolase) | 478700 |
| BRD8 | bromodomain containing 8 | 474692 |
| BTBD7 | BTB (POZ) domain containing 7 | 490834 |
| BTD | biotinidase | 477059 |
| BTG2 | BTG family, member 2 | 488559 |
| BUB1 | BUB1 mitotic checkpoint serine/threonine kinase | 100682599 |
| C1QA | complement C1q A chain | 478194 |
| C1S | complement component 1, s subcomponent | 486714 |
| C3 | complement C3 | 476728 |
| CACHD1 | cache domain containing 1 | 479541 |
| CACNB1 | calcium channel, voltage-dependent, beta 1 subunit | 491030 |
| CACNB4 | calcium voltage-gated channel auxiliary subunit beta 4 | 609361 |
| CACNG5 | calcium voltage-gated channel auxiliary subunit gamma 5 | 490905 |
| CACNG8 | calcium channel, voltage-dependent, gamma subunit 8 | 611498 |
| CAD | carbamoyl-phosphate synthetase 2, aspartate transcarbamylase, and dihydroorotase | 483009 |
| CADM1 | cell adhesion molecule 1 | 479432 |
| CADM2 | cell adhesion molecule 2 | 487680 |
| CADM3 | cell adhesion molecule 3 | 610435 |
| CADPS | calcium dependent secretion activator | 484705 |
| CAMK2D | calcium/calmodulin-dependent protein kinase II delta | 610764 |
| CAMK2G | calcium/calmodulin-dependent protein kinase II gamma | 489050 |
| CAP2 | cyclase associated actin cytoskeleton regulatory protein 2 | 478731 |
| CAPN2 | calpain 2, (m/II) large subunit | 480118 |
| CAPN3 | calpain 3, (p94) | 487518 |
| CAPN7 | calpain 7 | 477062 |
| CASKIN1 | CASK interacting protein 1 | 610943 |
| CASP6 | caspase 6, apoptosis-related cysteine peptidase | 487899 |
| CASQ1 | calsequestrin 1 | 608401 |
| CAT | catalase | 403474 |
| CAV1 | caveolin 1 | 403980 |
| CAV2 | caveolin 2 | 475294 |
| CBFB | core-binding factor, beta subunit | 479690 |
| CBR3 | carbonyl reductase 3 | 487748 |
| CBR4 | carbonyl reductase 4 | 477352 |
| CBS | cystathionine-beta-synthase | 611071 |
| CBX5 | chromobox 5 | 477593 |
| CCBL1 | cysteine conjugate-beta lyase, cytoplasmic | 491310 |
| CCBL2 | cysteine conjugate-beta lyase 2 | 479959 |
| CCDC102A | coiled-coil domain containing 102A | 487263 |
| CCDC122 | coiled-coil domain containing 122 | 485460 |
| CCDC18 | coiled-coil domain containing 18 | 479946 |
| CCDC25 | coiled-coil domain containing 25 | 612822 |
| CCDC3 | coiled-coil domain containing 3 | 607593 |
| CCDC34 | coiled-coil domain containing 34 | 476895 |
| CCDC50 | coiled-coil domain containing 50 | 478675 |
| CCDC51 | coiled-coil domain containing 51 | 609083 |
| CCDC6 | coiled-coil domain containing 6 | 488993 |
| CCDC8 | coiled-coil domain containing 8 | 484430 |
| CCDC90A | coiled-coil domain containing 90A | 610095 |
| CCDC90B | coiled-coil domain containing 90B | 476785 |
| CCL2 | chemokine (C-C motif) ligand 2 | 403981 |
| CCL3 | chemokine (C-C motif) ligand 3 | 448787 |
| CCL5 | C-C motif chemokine ligand 5 | 403522 |
| CCND1 | cyclin D1 | 449028 |
| CCND2 | cyclin D2 | 611782 |
| CCND3 | cyclin D3 | 608847 |
| CCPG1 | cell cycle progression 1 | 487566 |
| CCR2 | chemokine (C-C motif) receptor 2 | 484790 |
| CD109 | CD109 molecule | 474970 |
| CD14 | CD14 molecule | 607076 |
| CD151 | CD151 molecule | 475992 |
| CD164 | CD164 molecule | 475020 |
| CD302 | CD302 molecule | 100126287 |
| CD320 | CD320 molecule | 476721 |
| CD34 | CD34 molecule | 415130 |
| CD38 | CD38 molecule | 403756 |
| CD44 | CD44 molecule | 403939 |
| CD52 | CD52 molecule | 403918 |
| CD63 | CD63 molecule | 474391 |
| CD72 | CD72 molecule | 481595 |
| CDC14A | cell division cycle 14A | 479926 |
| CDC20 | cell division cycle 20 | 100855774 |
| CDC25B | cell division cycle 25B | 485790 |
| CDC42BPG | CDC42 binding protein kinase gamma (DMPK-like) | 483757 |
| CDC42EP4 | CDC42 effector protein (Rho GTPase binding) 4 | 475907 |
| CDC42SE1 | CDC42 small effector 1 | 608900 |
| CDC73 | cell division cycle 73 | 478955 |
| CDH10 | cadherin 10 | 489248 |
| CDH19 | cadherin 19 | 483948 |
| CDH2 | cadherin 2 | 480169 |
| CDK5RAP2 | CDK5 regulatory subunit associated protein 2 | 612705 |
| CDK6 | cyclin dependent kinase 6 | 609920 |
| CDKN1A | cyclin dependent kinase inhibitor 1A | 474890 |
| CDKN1B | cyclin-dependent kinase inhibitor 1B | 403429 |
| CDSN | corneodesmosin | 607062 |
| CDT1 | chromatin licensing and DNA replication factor 1 | 479616 |
| CEBPD | CCAAT enhancer binding protein delta | 606783 |
| CELA1 | chymotrypsin like elastase family member 1 | 403515 |
| CELSR1 | cadherin EGF LAG seven-pass G-type receptor 1 | 481203 |
| CEP192 | centrosomal protein 192kDa | 480216 |
| CEP350 | centrosomal protein 350kDa | 480028 |
| CEP63 | centrosomal protein 63kDa | 477075 |
| CFLAR | CASP8 and FADD-like apoptosis regulator | 488471 |
| CFP | complement factor properdin | 491859 |
| CGRRF1 | cell growth regulator with ring finger domain 1 | 480328 |
| CH25H | cholesterol 25-hydroxylase | 100856263 |
| CHAC1 | ChaC glutathione specific gamma-glutamylcyclotransferase 1 | 487504 |
| CHCHD2 | coiled-coil-helix-coiled-coil-helix domain containing 2 | 479702 |
| CHCHD3 | coiled-coil-helix-coiled-coil-helix domain containing 3 | 607574 |
| CHD1 | chromodomain helicase DNA binding protein 1 | 488891 |
| CHD1L | chromodomain helicase DNA binding protein 1-like | 475820 |
| CHD9 | chromodomain helicase DNA binding protein 9 | 478128 |
| CHI3L1 | chitinase 3-like 1 (cartilage glycoprotein-39) | 490222 |
| CHIC2 | cysteine-rich hydrophobic domain 2 | 611250 |
| CHPT1 | choline phosphotransferase 1 | 610214 |
| CHRAC1 | chromatin accessibility complex 1(CHRAC1) | 607681 |
| CHRDL1 | chordin-like 1 | 492066 |
| CHST1 | carbohydrate (keratan sulfate Gal-6) sulfotransferase 1 | 483642 |
| CHST11 | carbohydrate (chondroitin 4) sulfotransferase 11 | 481299 |
| CHST2 | carbohydrate (N-acetylglucosamine-6-O) sulfotransferase 2 | 485701 |
| CHST7 | carbohydrate (N-acetylglucosamine 6-O) sulfotransferase 7 | 491852 |
| CHSY1 | chondroitin sulfate synthase 1 | 488704 |
| CIB1 | calcium and integrin binding 1 (calmyrin) | 479044 |
| CIDEA | cell death-inducing DFFA-like effector a | 490559 |
| CIDEB | cell death-inducing DFFA-like effector b | 608437 |
| CKAP4 | cytoskeleton-associated protein 4 | 481295 |
| CKAP5 | cytoskeleton associated protein 5 | 475986 |
| CKB | creatine kinase B | 100855552 |
| CKLF | chemokine-like factor | 611371 |
| CLASP2 | cytoplasmic linker associated protein 2 | 477012 |
| CLCC1 | chloride channel CLIC-like 1 | 479918 |
| CLCF1 | cardiotrophin-like cytokine factor 1 | 483697 |
| CLCN2 | chloride channel 2 | 488106 |
| CLDN10 | claudin 10 | 476963 |
| CLDN12 | claudin 12 | 608397 |
| CLDN19 | claudin 19 | 607005 |
| CLEC5A | C-type lectin domain family 5 member A | 609161 |
| CLIP4 | CAP-GLY domain containing linker protein family, member 4 | 483020 |
| CLMN | calmin (calponin-like, transmembrane) | 480427 |
| CLN6 | ceroid-lipofuscinosis, neuronal 6, late infantile, variant | 497068 |
| CLPB | ClpB homolog, mitochondrial AAA ATPase chaperonin | 476815 |
| CLPX | caseinolytic mitochondrial matrix peptidase chaperone subunit | 609344 |
| CLSTN2 | calsyntenin 2 | 477093 |
| CLTB | clathrin light chain B | 489104 |
| CLTC | clathrin, heavy chain (Hc) | 480578 |
| CLU | clusterin | 442971 |
| CLYBL | citrate lyase beta like | 476974 |
| CMTM5 | CKLF like MARVEL transmembrane domain containing 5 | 608193 |
| CMTM6 | CKLF-like MARVEL transmembrane domain containing 6 | 609718 |
| CMYA5 | cardiomyopathy associated 5 | 479170 |
| CNIH4 | cornichon family AMPA receptor auxiliary protein 4 | 480116 |
| CNN3 | calponin 3, acidic | 479937 |
| CNNM2 | cyclin M2 | 486866 |
| CNNM3 | cyclin M3 | 481345 |
| CNNM4 | cyclin and CBS domain divalent metal cation transport mediator 4 | 481346 |
| CNOT10 | CCR4-NOT transcription complex, subunit 10 | 612778 |
| CNPY1 | canopy FGF signaling regulator 1 | 100856196 |
| CNTD1 | cyclin N-terminal domain containing 1 | 490958 |
| CNTF | ciliary neurotrophic factor | 483464 |
| CNTFR | ciliary neurotrophic factor receptor | 442941 |
| CNTN1 | contactin 1 | 477641 |
| CNTN2 | contactin 2 | 100685268 |
| CNTNAP2 | contactin associated protein-like 2 | 100687859 |
| COG2 | component of oligomeric golgi complex 2 | 488978 |
| COL12A1 | collagen type XII alpha 1 chain | 481881 |
| COL16A1 | collagen, type XVI, alpha 1 | 487318 |
| COL23A1 | collagen, type XXIII, alpha 1 | 607556 |
| COL4A3 | collagen type IV alpha 3 chain | 403842 |
| COL4A4 | collagen, type IV, alpha 4 | 403841 |
| COL4A5 | collagen, type IV, alpha 5 | 403466 |
| COL5A2 | collagen, type V, alpha 2 | 478836 |
| COL6A1 | collagen type VI alpha 1 chain | 403668 |
| COL6A2 | collagen, type VI, alpha 2 | 100856491 |
| COMT | catechol-O-methyltransferase | 445450 |
| COPZ2 | coatomer protein complex, subunit zeta 2 | 491050 |
| COQ10B | coenzyme Q10B | 478853 |
| COQ9 | coenzyme Q9 | 478110 |
| CORO1A | coronin 1A | 489949 |
| CORO1C | coronin 1C | 486318 |
| COX4NB | COX4 neighbor | 479624 |
| CP | ceruloplasmin | 442963 |
| CPE | carboxypeptidase E | 475492 |
| CPEB3 | cytoplasmic polyadenylation element binding protein 3 | 486801 |
| CPEB4 | cytoplasmic polyadenylation element binding protein 4 | 479287 |
| CPNE1 | copine I | 477213 |
| CPNE2 | copine II | 478115 |
| CPNE3 | copine III | 487034 |
| CPNE8 | copine 8(CPNE8) | 477646 |
| CPT1A | carnitine palmitoyltransferase 1A | 403583 |
| CPT2 | carnitine palmitoyltransferase 2 | 489585 |
| CPXM1 | carboxypeptidase X (M14 family), member 1 | 485802 |
| CRAT | carnitine acetyltransferase | 491304 |
| CRIP2 | cysteine-rich protein 2 | 612710 |
| CRISPLD2 | cysteine-rich secretory protein LCCL domain containing 2 | 489677 |
| CRLF3 | cytokine receptor-like factor 3 | 491157 |
| CROT | carnitine O-octanoyltransferase | 482283 |
| CRTAP | cartilage associated protein | 485577 |
| CRTC3 | CREB regulated transcription coactivator 3 | 488747 |
| CRY1 | cryptochrome circadian regulator 1 | 474528 |
| CRY2 | cryptochrome circadian regulator 2 | 483641 |
| CS | citrate synthase | 474403 |
| CSDC2 | cold shock domain containing C2, RNA binding | 481231 |
| CSF1R | colony stimulating factor 1 receptor | 489188 |
| CSPG5 | chondroitin sulfate proteoglycan 5 | 609188 |
| CSPP1 | centrosome and spindle pole associated protein 1 | 477902 |
| CSRP1 | cysteine and glycine-rich protein 1 | 607054 |
| CTBS | chitobiase | 490188 |
| CTDSP1 | CTD small phosphatase 1 | 100856365 |
| CTDSP2 | CTD small phosphatase 2 | 481135 |
| CTDSPL | CTD small phosphatase like | 608986 |
| CTGF | connective tissue growth factor | 476202 |
| CTH | cystathionine gamma-lyase | 479991 |
| CTNNA2 | catenin alpha 2 | 483088 |
| CTNNBIP1 | catenin, beta interacting protein 1 | 608589 |
| CTNND1 | catenin delta 1 | 483489 |
| CTNND2 | catenin delta 2 | 488048 |
| CTNS | cystinosis, nephropathic | 491220 |
| CTSB | cathepsin B | 486077 |
| CTSD | cathepsin D | 483662 |
| CTSE | cathepsin E | 488577 |
| CTSF | cathepsin F | 476010 |
| CTSO | cathepsin O | 482665 |
| CTSZ | cathepsin Z | 611983 |
| CTTN | cortactin | 610283 |
| CUTC | cutC copper transporter | 477793 |
| CWF19L2 | CWF19 like cell cycle control factor 2 | 479454 |
| CXCL10 | chemokine (C-X-C motif) ligand 10 | 478432 |
| CXCL14 | chemokine (C-X-C motif) ligand 14 | 610078 |
| CXCL8 | C-X-C motif chemokine ligand 8 | 403850 |
| CYB5R3 | cytochrome b5 reductase 3 | 474479 |
| CYBA | cytochrome b-245, alpha polypeptide | 489664 |
| CYFIP1 | cytoplasmic FMR1 interacting protein 1 | 479001 |
| CYP1B1 | cytochrome P450, family 1, subfamily B, polypeptide 1 | 483038 |
| CYP46A1 | cytochrome P450, family 46, subfamily A, polypeptide 1 | 480432 |
| CYP7B1 | cytochrome P450, family 7, subfamily B, polypeptide 1 | 486973 |
| CYR61 | cysteine-rich, angiogenic inducer, 61 | 479967 |
| D2HGDH | D-2-hydroxyglutarate dehydrogenase | 609641 |
| DAAM2 | dishevelled associated activator of morphogenesis 2 | 481783 |
| DAB1 | DAB1, reelin adaptor protein | 610344 |
| DAB2 | DAB2, clathrin adaptor protein | 479353 |
| DACH1 | dachshund family transcription factor 1 | 485489 |
| DAG1 | dystroglycan 1 | 476623 |
| DARC | Duffy blood group, chemokine receptor | 478969 |
| DAZAP2 | DAZ associated protein 2 | 477605 |
| DBI | diazepam binding inhibitor, acyl-CoA binding protein | 476115 |
| DBT | dihydrolipoamide branched chain transacylase E2 | 479929 |
| DBX2 | developing brain homeobox 2 | 486598 |
| DCHS1 | dachsous cadherin-related 1 | 476837 |
| DCI | dodecenoyl-Coenzyme A delta isomerase | 490059 |
| DCN | decorin | 403904 |
| DCTD | dCMP deaminase | 607328 |
| DCXR | dicarbonyl and L-xylulose reductase | 475926 |
| DDAH1 | dimethylarginine dimethylaminohydrolase 1 | 490182 |
| DDAH2 | dimethylarginine dimethylaminohydrolase 2 | 474846 |
| DDB2 | damage-specific DNA binding protein 2, 48kDa | 483626 |
| DDHD1 | DDHD domain containing 1 | 480326 |
| DDIT3 | DNA-damage-inducible transcript 3 | 607439 |
| DDIT4 | DNA-damage-inducible transcript 4 | 489038 |
| DDIT4L | DNA-damage-inducible transcript 4-like | 487877 |
| DDO | D-aspartate oxidase | 475026 |
| DDR2 | discoidin domain receptor tyrosine kinase 2 | 478987 |
| DDT | D-dopachrome tautomerase | 607589 |
| DECR1 | 2,4-dienoyl CoA reductase 1, mitochondrial | 477938 |
| DEFB1 | defensin beta 1 | 611241 |
| DENND1A | DENN/MADD domain containing 1A | 609706 |
| DENND2A | DENN/MADD domain containing 2A | 482766 |
| DENND4C | DENN/MADD domain containing 4C | 474721 |
| DERL1 | Der1-like domain family, member 1 | 475086 |
| DERL2 | Der1-like domain family, member 2 | 606991 |
| DET1 | DET1, COP1 ubiquitin ligase partner | 479036 |
| DEXI | similar to MYLE protein (Dexamethasone-induced protein) | 609975 |
| DGAT1 | diacylglycerol O-acyltransferase 1 | 482093 |
| DGKB | diacylglycerol kinase, beta 90kDa | 482328 |
| DHCR7 | 7-dehydrocholesterol reductase | 483675 |
| DHDH | dihydrodiol dehydrogenase (dimeric) | 403786 |
| DHRS1 | dehydrogenase/reductase (SDR family) member 1 | 480270 |
| DHRS4 | dehydrogenase/reductase (SDR family) member 4 | 480254 |
| DHRS7 | dehydrogenase/reductase (SDR family) member 7 | 480342 |
| DHTKD1 | dehydrogenase E1 and transketolase domain containing 1 | 478008 |
| DHX33 | DEAH (Asp-Glu-Ala-His) box polypeptide 33 | 607226 |
| DHX35 | DEAH (Asp-Glu-Ala-His) box polypeptide 35 | 485872 |
| DHX40 | DEAH (Asp-Glu-Ala-His) box polypeptide 40 | 480577 |
| DIO2 | deiodinase, iodothyronine, type II | 490813 |
| DIRC2 | disrupted in renal carcinoma 2 | 478588 |
| DISP1 | dispatched RND transporter family member 1 | 488604 |
| DKK3 | dickkopf WNT signaling pathway inhibitor 3 | 476857 |
| DLD | dihydrolipoamide dehydrogenase | 403978 |
| DLEU7 | deleted in lymphocytic leukemia, 7 | 106557548 |
| DLG5 | discs large MAGUK scaffold protein 5 | 489061 |
| DLGAP1 | DLG associated protein 1 | 480194 |
| DLL1 | delta like canonical Notch ligand 1 | 100688936 |
| DMD | dystrophin | 606758 |
| DMP1 | dentin matrix acidic phosphoprotein 1 | 487849 |
| DMRT3 | doublesex and mab-3 related transcription factor 3 | 609208 |
| DMRTA2 | DMRT like family A2 | 100856321 |
| DNAJB9 | DnaJ (Hsp40) homolog, subfamily B, member 9 | 475286 |
| DNAJC19 | DnaJ (Hsp40) homolog, subfamily C, member 19 | 488090 |
| DNAJC3 | DnaJ (Hsp40) homolog, subfamily C, member 3 | 476966 |
| DNER | delta/notch-like EGF repeat containing | 609904 |
| DNMBP | dynamin binding protein | 477794 |
| DOCK1 | dedicator of cytokinesis 1 | 486934 |
| DOCK11 | dedicator of cytokinesis 11 | 492089 |
| DOCK7 | dedicator of cytokinesis 7 | 479548 |
| DOLK | dolichol kinase | 480701 |
| DPF3 | D4, zinc and double PHD fingers, family 3 | 611798 |
| DPH5 | diphthamide biosynthesis 5 | 612092 |
| DPP7 | dipeptidyl-peptidase 7 | 607003 |
| DPP8 | dipeptidyl-peptidase 8 | 487605 |
| DPY19L3 | dpy-19 like C-mannosyltransferase 3 | 611998 |
| DPYSL3 | dihydropyrimidinase like 3 | 487204 |
| DRP2 | dystrophin related protein 2 | 480984 |
| DSN1 | DSN1 homolog, MIS12 kinetochore complex component | 610279 |
| DST | dystonin | 474948 |
| DTNA | dystrobrevin, alpha | 490488 |
| DULLARD | CTD nuclear envelope phosphatase 1 | 607484 |
| DUSP1 | dual specificity phosphatase 1 | 489117 |
| DUSP11 | dual specificity phosphatase 11 | 483110 |
| DUSP6 | dual specificity phosphatase 6 | 482594 |
| DYRK1A | dual-specificity tyrosine-(Y)-phosphorylation regulated kinase 1A | 487755 |
| E130304F04RIK | NIM1 serine/threonine protein kinase | 489218 |
| E2F5 | E2F transcription factor 5, p130-binding | 611103 |
| E2F6 | E2F transcription factor 6 | 609996 |
| E2F7 | E2F transcription factor 7 | 482575 |
| ECE2 | endothelin converting enzyme 2 | 488105 |
| ECHDC1 | enoyl Coenzyme A hydratase domain containing 1 | 609850 |
| ECHS1 | enoyl Coenzyme A hydratase, short chain, 1, mitochondrial | 480828 |
| ECM1 | extracellular matrix protein 1 | 608791 |
| EDN1 | endothelin 1 | 403424 |
| EDNRA | endothelin receptor type A | 450187 |
| EDNRB | endothelin receptor type B | 403862 |
| EEA1 | early endosome antigen 1 | 475424 |
| EEF2K | eukaryotic elongation factor-2 kinase | 479812 |
| EFCAB2 | EF-hand calcium binding domain 2 | 611783 |
| EFEMP1 | EGF-containing fibulin-like extracellular matrix protein 1 | 474604 |
| EFHD1 | EF-hand domain family member D1 | 486164 |
| EFHD2 | EF-hand domain family member D2 | 607254 |
| EFS | embryonal Fyn-associated substrate | 480251 |
| EGFL6 | EGF-like-domain, multiple 6 | 491745 |
| EGFR | epidermal growth factor receptor | 404306 |
| EGLN3 | egl-9 family hypoxia inducible factor 3 | 480286 |
| EGR1 | early growth response 1 | 481528 |
| EGR3 | early growth response 3 | 486121 |
| EHD2 | EH-domain containing 2 | 484417 |
| EHMT2 | euchromatic histone lysine methyltransferase 2 | 474851 |
| EIF2B5 | eukaryotic translation initiation factor 2B, subunit 5 epsilon | 100856343 |
| EIF4EBP1 | eukaryotic translation initiation factor 4E binding protein 1 | 475590 |
| EIF4EBP2 | eukaryotic translation initiation factor 4E binding protein 2 | 489027 |
| EIF4G3 | eukaryotic translation initiation factor 4 gamma, 3 | 478202 |
| EIF5 | eukaryotic translation initiation factor 5 | 480442 |
| ELAVL1 | ELAV like RNA binding protein 1 | 611201 |
| ELF5 | E74-like factor 5 (ets domain transcription factor) | 608908 |
| ELK3 | ELK3, ETS transcription factor | 482613 |
| ELMO2 | engulfment and cell motility 2 | 477251 |
| ELOVL5 | ELOVL fatty acid elongase 5 | 610377 |
| ELP4 | elongator acetyltransferase complex subunit 4 | 483442 |
| EML3 | echinoderm microtubule associated protein like 3 | 483787 |
| EMP1 | epithelial membrane protein 1 | 486676 |
| EMP2 | epithelial membrane protein 2 | 490010 |
| EMX2 | empty spiracles homeobox 2 | 610697 |
| EN2 | engrailed homeobox 2 | 611369 |
| ENDOG | endonuclease G | 608916 |
| ENDOU | endonuclease, polyU-specific | 609443 |
| ENO1 | enolase 1, (alpha) | 479597 |
| ENPP4 | ectonucleotide pyrophosphatase/phosphodiesterase 4 (putative) | 481823 |
| ENTPD1 | ectonucleoside triphosphate diphosphohydrolase 1 | 486810 |
| ENTPD2 | ectonucleoside triphosphate diphosphohydrolase 2 | 491241 |
| EPAS1 | endothelial PAS domain protein 1 | 474578 |
| EPC1 | enhancer of polycomb homolog 1 | 477970 |
| EPDR1 | ependymin related 1 | 609403 |
| EPHA4 | EPH receptor A4 | 478925 |
| EPHB3 | EPH receptor B3 | 488108 |
| EPHX1 | epoxide hydrolase 1, microsomal (xenobiotic) | 480113 |
| EPHX2 | epoxide hydrolase 2, cytoplasmic | 477373 |
| EPS15 | epidermal growth factor receptor pathway substrate 15 | 475356 |
| EPS8 | epidermal growth factor receptor pathway substrate 8 | 477686 |
| ERBB2 | erb-b2 receptor tyrosine kinase 2 | 403883 |
| ERN1 | endoplasmic reticulum to nucleus signaling 1 | 610766 |
| ESCO1 | establishment of sister chromatid cohesion N-acetyltransferase 1 | 490523 |
| ESD | esterase D | 607116 |
| ESPL1 | extra spindle pole bodies like 1, separase | 607879 |
| ESR1 | estrogen receptor 1 | 403640 |
| ESRRA | estrogen-related receptor alpha | 403169 |
| ETAA1 | Ewing tumor-associated antigen 1 | 474621 |
| ETFA | electron-transfer-flavoprotein, alpha polypeptide | 610134 |
| ETFB | electron-transfer-flavoprotein, beta polypeptide | 476400 |
| ETFDH | electron-transferring-flavoprotein dehydrogenase | 475480 |
| ETV4 | ets variant 4 | 403641 |
| ETV5 | ets variant 5 | 607474 |
| EVC | Ellis van Creveld syndrome | 611166 |
| EVC2 | Ellis van Creveld syndrome 2 | 611156 |
| EXOSC3 | exosome component 3 | 481616 |
| EXTL3 | exostoses (multiple)-like 3 | 486091 |
| EYA1 | EYA transcriptional coactivator and phosphatase 1 | 477910 |
| EYA4 | EYA transcriptional coactivator and phosphatase 4 | 483991 |
| EZR | ezrin | 484056 |
| F2R | coagulation factor II (thrombin) receptor | 488942 |
| F3 | coagulation factor III (thromboplastin, tissue factor) | 490153 |
| FABP5 | fatty acid binding protein 5 | 477923 |
| FABP7 | fatty acid binding protein 7, brain | 476278 |
| FADS2 | fatty acid desaturase 2 | 483792 |
| FADS6 | fatty acid desaturase domain family, member 6 | 609709 |
| FAHD2A | fumarylacetoacetate hydrolase domain containing 2A | 475745 |
| FAM129B | family with sequence similarity 129 member B | 609251 |
| FARP1 | FERM, RhoGEF (ARHGEF) and pleckstrin domain protein 1 | 476970 |
| FARP2 | FERM, RhoGEF and pleckstrin domain protein 2 | 486205 |
| FARS2 | phenylalanyl-tRNA synthetase 2, mitochondrial | 488204 |
| FASLG | Fas ligand | 442968 |
| FAT1 | FAT atypical cadherin 1 | 475621 |
| FBF1 | Fas (TNFRSF6) binding factor 1 | 483321 |
| FBLN1 | fibulin 1 | 474468 |
| FBLN2 | fibulin 2 | 484634 |
| FBLN5 | fibulin 5 | 480227 |
| FBXL3 | F-box and leucine-rich repeat protein 3 | 485499 |
| FBXL4 | F-box and leucine-rich repeat protein 4 | 481932 |
| FBXO2 | F-box protein 2 | 478231 |
| FBXO3 | F-box protein 3 | 475944 |
| FBXO30 | F-box protein 30 | 484023 |
| FBXO4 | F-box protein 4 | 489220 |
| FCHO2 | FCH domain only 2 | 478094 |
| FDFT1 | farnesyl-diphosphate farnesyltransferase 1 | 477362 |
| FDPS | farnesyl diphosphate synthase(FDPS) | 480129 |
| FDXR | ferredoxin reductase | 475910 |
| FEZF2 | FEZ family zinc finger 2 | 476568 |
| FGD4 | FYVE, RhoGEF and PH domain containing 4 | 486615 |
| FGD6 | FYVE, RhoGEF and PH domain containing 6 | 482608 |
| FGF1 | fibroblast growth factor 1 | 607724 |
| FGF12 | fibroblast growth factor 12 | 478676 |
| FGF14 | fibroblast growth factor 14 | 485537 |
| FGF2 | fibroblast growth factor 2 | 403857 |
| FGFBP3 | fibroblast growth factor binding protein 3 | 608068 |
| FGFR1 | fibroblast growth factor receptor 1 | 100856477 |
| FGFR2 | fibroblast growth factor receptor 2 | 415125 |
| FGFR3 | fibroblast growth factor receptor 3 | 488808 |
| FGFRL1 | fibroblast growth factor receptor-like 1 | 488865 |
| FGL2 | fibrinogen like 2 | 475902 |
| FHL1 | four and a half LIM domains 1 | 492162 |
| FIGNL1 | fidgetin like 1 | 483232 |
| FIP1L1 | factor interacting with PAPOLA and CPSF1 | 612980 |
| FJX1 | four jointed box 1 | 483425 |
| FKBP10 | FK506 binding protein 10 | 490975 |
| FKBP14 | FK506 binding protein 14 | 611546 |
| FKBP5 | FK506 binding protein 5 | 481759 |
| FKBP7 | FK506 binding protein 7 | 488424 |
| FLNA | filamin A | 481084 |
| FLNC | filamin C | 482266 |
| FMN2 | formin 2 | 490366 |
| FMO1 | flavin containing monooxygenase 1 | 403604 |
| FMO2 | flavin containing monooxygenase 2 | 480076 |
| FMO5 | flavin containing monooxygenase 5 | 475819 |
| FNBP1 | formin binding protein 1 | 480695 |
| FNDC3B | fibronectin type III domain containing 3B | 488169 |
| FNDC4 | fibronectin type III domain containing 4 | 483014 |
| FNDC5 | fibronectin type III domain containing 5 | 487302 |
| FOLH1 | folate hydrolase (prostate-specific membrane antigen) 1 | 476775 |
| FOS | v-fos FBJ murine osteosarcoma viral oncogene homolog | 490792 |
| FOSB | FBJ murine osteosarcoma viral oncogene homolog B | 484445 |
| FOSL1 | FOS like 1, AP-1 transcription factor subunit | 483724 |
| FOSL2 | FOS like 2, AP-1 transcription factor subunit | 608412 |
| FOXG1 | forkhead box G1 | 490636 |
| FOXO1 | forkhead box O1 | 477295 |
| FOXRED2 | FAD-dependent oxidoreductase domain containing 2 | 474518 |
| FREM2 | FRAS1 related extracellular matrix protein 2 | 486002 |
| FRMD4B | FERM domain containing 4B | 484693 |
| FRMD5 | FERM domain containing 5 | 478282 |
| FRMPD1 | FERM and PDZ domain containing 1 | 481614 |
| FRRS1 | ferric-chelate reductase 1 | 490144 |
| FRY | FRY microtubule binding protein | 477318 |
| FSCN1 | fascin actin-bundling protein 1 | 489880 |
| FSTL1 | follistatin-like 1 | 608384 |
| FUCA1 | fucosidase, alpha-L- 1, tissue | 403929 |
| FUCA2 | fucosidase, alpha-L- 2, plasma | 484016 |
| FUK | fucokinase | 489715 |
| FUNDC2 | FUN14 domain containing 2 | 613014 |
| FUT9 | fucosyltransferase 9 (alpha (1,3) fucosyltransferase) | 449027 |
| FXN | frataxin | 609051 |
| FXYD1 | FXYD domain containing ion transport regulator 1 | 476487 |
| FZD1 | frizzled class receptor 1 | 482294 |
| FZD2 | frizzled class receptor 2 | 490937 |
| FZD4 | frizzled class receptor 4 | 485149 |
| FZD5 | frizzled class receptor 5 | 100855544 / 488493 |
| FZD7 | frizzled class receptor 7 | 488478 |
| FZD9 | frizzled class receptor 9 | 489809 |
| G0S2 | G0/G1 switch 2 | 609704 |
| GABBR1 | gamma-aminobutyric acid (GABA) B receptor, 1 | 488340 |
| GABPA | GA binding protein transcription factor subunit alpha | 478394 |
| GABRA2 | gamma-aminobutyric acid (GABA) A receptor, alpha 2 | 482131 |
| GABRA4 | gamma-aminobutyric acid (GABA) A receptor, alpha 4 | 482132 |
| GABRG1 | gamma-aminobutyric acid (GABA) A receptor, gamma 1 | 100856619 |
| GADD45A | growth arrest and DNA damage inducible alpha | 100855728 |
| GADD45B | growth arrest and DNA damage inducible beta | 485069 |
| GADD45G | growth arrest and DNA-damage-inducible, gamma | 484198 |
| GALC | galactosylceramidase | 403916 |
| GALM | galactose mutarotase (aldose 1-epimerase) | 483039 |
| GALNT1 | polypeptide N-acetylgalactosaminyltransferase 1 | 480161 |
| GALNTL2 | polypeptide N-acetylgalactosaminyltransferase 15 | 477056 |
| GALR2 | galanin receptor 2 | 483325 |
| GAP43 | growth associated protein 43 | 478572 |
| GAS1 | growth arrest-specific 1 | 607496 |
| GAS2L1 | growth arrest-specific 2 like 1 | 486342 |
| GATAD1 | GATA zinc finger domain containing 1 | 482301 |
| GBAS | glioblastoma amplified sequence | 479700 |
| GBE1 | glucan (1,4-alpha-), branching enzyme 1 | 478380 |
| GCAT | glycine C-acetyltransferase | 481262 |
| GCDH | glutaryl-Coenzyme A dehydrogenase | 476696 |
| GCH1 | GTP cyclohydrolase 1 | 609393 |
| GCSH | glycine cleavage system protein H | 479633 |
| GDA | guanine deaminase(GDA) | 484169 |
| GDF10 | growth differentiation factor 10 | 611168 |
| GDF15 | growth differentiation factor 15 | 484822 |
| GDNF | glial cell derived neurotrophic factor | 489224 |
| GEMIN8 | gem (nuclear organelle) associated protein 8 | 480843 |
| GFAP | Glial fibrillary acidic protein | 480495 |
| GFRA1 | GDNF family receptor alpha 1 | 609114 |
| GGH | gamma-glutamyl hydrolase | 100856436 |
| GHDC | GH3 domain containing | 607630 |
| GHITM | growth hormone inducible transmembrane protein | 479266 |
| GHR | growth hormone receptor | 403721 |
| GJA1 | gap junction protein, alpha 1 | 403418 |
| GJB2 | gap junction protein, beta 2 | 403570 |
| GJB6 | gap junction protein beta 6 | 100688824 |
| GLB1L | galactosidase, beta 1-like | 478917 |
| GLDC | glycine decarboxylase | 481534 |
| GLI2 | GLI family zinc finger 2 | 100682984 |
| GLI3 | GLI family zinc finger 3 | 483244 |
| GLIS2 | GLIS family zinc finger 2 | 490028 |
| GLIS3 | GLIS family zinc finger 3 | 484179 |
| GLO1 | glyoxalase I | 474894 |
| GLRB | glycine receptor beta | 475477 |
| GLS | glutaminase | 488448 |
| GLT25D2 | glycosyltransferase 25 domain containing 2 | 608413 |
| GLUD1 | glutamate dehydrogenase 1 | 100684847 |
| GLUL | glutamate-ammonia ligase (glutamine synthetase) | 403443 |
| GM2A | GM2 ganglioside activator | 479324 |
| GMFB | glia maturation factor beta | 100856272 |
| GNA13 | guanine nucleotide binding protein (G protein), alpha 13 | 490901 |
| GNA14 | G protein subunit alpha 14 | 484156 |
| GNAI2 | G protein subunit alpha i2 | 442957 |
| GNAO1 | G protein subunit alpha o1 | 609157 |
| GNG3 | G protein subunit gamma 3(GNG3) | 483783 |
| GNPDA2 | glucosamine-6-phosphate deaminase 2 | 608049 |
| GNS | glucosamine (N-acetyl)-6-sulfatase | 474429 |
| GOLGA1 | golgi autoantigen, golgin subfamily a, 1 | 480728 |
| GOLPH3 | golgi phosphoprotein 3 | 489241 |
| GP2 | glycoprotein 2 | 442972 |
| GPAM | glycerol-3-phosphate acyltransferase, mitochondrial | 486890 |
| GPC4 | glypican 4 | 492144 |
| GPC5 | glypican 5 | 485518 |
| GPC6 | glypican 6 | 476962 |
| GPD1 | glycerol-3-phosphate dehydrogenase 1 | 607942 |
| GPD2 | glycerol-3-phosphate dehydrogenase 2 | 478755 |
| GPLD1 | glycosylphosphatidylinositol specific phospholipase D1 | 478735 |
| GPM6A | glycoprotein M6A | 475641 |
| GPM6B | glycoprotein M6B | 480842 |
| GPR125 | G protein-coupled receptor 125 | 488859 |
| GPR126 | G protein-coupled receptor 126 | 484013 |
| GPR137B | G protein-coupled receptor 137B | 479194 |
| GPR146 | G protein-coupled receptor 146 | 491603 |
| GPR173 | G protein-coupled receptor 173 | 491892 |
| GPR37L1 | G protein-coupled receptor 37 like 1 | 607285 |
| GPR84 | G protein-coupled receptor 84 | 100687941 |
| GPRC5B | G protein-coupled receptor class C group 5 member B | 100856475 |
| GPSM2 | G protein signaling modulator 2 | 490127 |
| GPX1 | glutathione peroxidase 1 | 442961 |
| GPX3 | glutathione peroxidase 3 | 489179 |
| GPX7 | glutathione peroxidase 7 | 475348 |
| GRAMD1C | GRAM domain containing 1C | 607747 |
| GRAMD3 | GRAM domain containing 3 | 474662 |
| GRB10 | growth factor receptor bound protein 10 | 607755 |
| GRB14 | growth factor receptor bound protein 14 | 478770 |
| GREB1 | similar to GREB1 protein isoform a | 610007 |
| GRHL1 | grainyhead like transcription factor 1 | 475664 |
| GRIA1 | glutamate ionotropic receptor AMPA type subunit 1 | 489168 |
| GRIA2 | glutamate ionotropic receptor AMPA type subunit 2 | 482667 |
| GRID2 | glutamate receptor, ionotropic, delta 2 | 487863 |
| GRIN2C | glutamate receptor, ionotropic, N-methyl D-aspartate 2C | 483302 |
| GRIN3A | glutamate receptor, ionotropic, N-methyl-D-aspartate 3A | 474789 |
| GRINA | glutamate receptor, ionotropic, N-methyl D-aspartate-associated protein 1 | 475118 |
| GRN | granulin | 480501 |
| GSR | glutathione reductase | 475596 |
| GSTK1 | glutathione S-transferase kappa 1 | 475518 |
| GSTM1 | glutathione S-transferase Mu 1 | 479912 |
| GSTO2 | glutathione S-transferase omega 2 | 477814 |
| GSTT1 | glutathione S-transferase theta 1 | 477556 |
| GSTZ1 | glutathione transferase zeta 1 | 490806 |
| GTF3C2 | general transcription factor IIIC subunit 2 | 483010 |
| GULP1 | GULP, engulfment adaptor PTB domain containing 1 | 478834 |
| GUSB | glucuronidase, beta | 403831 |
| H19 | H19, imprinted maternally expressed transcript (non-protein coding) | 100271858 |
| HACL1 | 2-hydroxyacyl-CoA lyase 1 | 477060 |
| HADH | hydroxyacyl-Coenzyme A dehydrogenase | 478506 |
| HADHB | hydroxyacyl-CoA dehydrogenase trifunctional multienzyme complex subunit beta | 607926 |
| HAPLN1 | hyaluronan and proteoglycan link protein 1 | 488921 |
| HAPLN3 | hyaluronan and proteoglycan link protein 3 | 609165 |
| HAVCR2 | hepatitis A virus cellular receptor 2 | 479318 |
| HBEGF | heparin-binding EGF-like growth factor | 607007 |
| HBP1 | HMG-box transcription factor 1 | 475886 |
| HCFC2 | host cell factor C2 | 475449 |
| HDDC2 | HD domain containing 2 | 476281 |
| HDDC3 | HD domain containing 3 | 488744 |
| HDHD2 | haloacid dehalogenase-like hydrolase domain containing 2 | 480146 |
| HECTD2 | HECT domain containing 2 | 486795 |
| HELB | helicase (DNA) B | 608595 |
| HEPACAM | hepatocyte cell adhesion molecule | 489305 |
| HEPH | hephaestin | 491926 |
| HES5 | hes family bHLH transcription factor 5 | 489614 |
| HEXA | hexosaminidase A (alpha polypeptide) | 487633 |
| HEXB | hexosaminidase B (beta polypeptide) | 478100 |
| HEXIM1 | hexamethylene bis-acetamide inducible 1 | 606811 |
| HEY1 | hairy/enhancer-of-split related with YRPW motif 1 | 403420 |
| HEYL | hairy/enhancer-of-split related with YRPW motif-like | 607804 |
| HGF | hepatocyte growth factor | 403441 |
| HHATL | hedgehog acyltransferase-like | 111089971 |
| HIBADH | 3-hydroxyisobutyrate dehydrogenase | 479610 |
| HIBCH | 3-hydroxyisobutyryl-Coenzyme A hydrolase | 607040 |
| HIF3A | hypoxia inducible factor 3, alpha subunit | 476429 |
| HIPK1 | homeodomain interacting protein kinase 1 | 475803 |
| HMCN1 | hemicentin 1 | 490318 |
| HMG20B | high-mobility group 20B | 612026 |
| HMGA1 | high mobility group AT-hook 1 | 442946 |
| HMGA2 | high mobility group AT-hook 2 | 100271859 |
| HMGB1 | high-mobility group box 1 | 403170 |
| HMGB2 | high-mobility group box 2 | 486068 |
| HMGCL | 3-hydroxymethyl-3-methylglutaryl-Coenzyme A lyase | 478187 |
| HMGCS1 | 3-hydroxy-3-methylglutaryl-Coenzyme A synthase 1 | 479344 |
| HMGCS2 | 3-hydroxy-3-methylglutaryl-Coenzyme A synthase 2 | 607923 |
| HMOX1 | heme oxygenase 1 | 442987 |
| HOPX | HOP homeobox | 100855799 |
| HOXA5 | homeobox A5 | 482370 |
| HOXB3 | homeobox B3 | 491054 |
| HOXB4 | homeobox B4 | 608915 |
| HOXB5 | homeobox B5 | 100686000 |
| HOXB6 | homeobox B6 | 491056 |
| HOXB8 | homeobox B8 | 491059 |
| HPGD | hydroxyprostaglandin dehydrogenase 15-(NAD) | 486073 |
| HPS1 | Hermansky-Pudlak syndrome 1 | 477791 |
| HRC | histidine rich calcium binding protein | 484386 |
| HRH1 | histamine receptor H1 | 403813 |
| HRSP12 | heat-responsive protein 12 | 475043 |
| HS2ST1 | heparan sulfate 2-O-sulfotransferase 1 | 479963 |
| HS3ST3B1 | heparan sulfate (glucosamine) 3-O-sulfotransferase 3B1 | 489516 |
| HSD11B1 | hydroxysteroid 11-beta dehydrogenase 1 | 449023 |
| HSD17B10 | hydroxysteroid (17-beta) dehydrogenase 10 | 480930 |
| HSD17B11 | hydroxysteroid (17-beta) dehydrogenase 11 | 487848 |
| HSD17B4 | hydroxysteroid (17-beta) dehydrogenase 4 | 474630 |
| HSD3B7 | hydroxy-delta-5-steroid dehydrogenase, 3 beta- and steroid delta-isomerase 7 | 489917 |
| HSDL2 | hydroxysteroid dehydrogenase like 2 | 474804 |
| HSPA2 | heat shock 70kDa protein 2 | 480355 |
| HSPA4L | heat shock 70kDa protein 4-like | 476089 |
| HSPA8 | heat shock protein family A (Hsp70) member 8 | 479406 |
| HSPB1 | heat shock protein family B (small) member 1 | 403979 |
| HSPB6 | heat shock protein family B (small) member 6 | 484574 |
| HSPB8 | heat shock 22kDa protein 8 | 403553 |
| HTRA1 | HtrA serine peptidase 1 | 477852 |
| HUS1 | HUS1 checkpoint clamp component | 606789 |
| HYAL1 | hyaluronoglucosaminidase 1 | 608602 |
| IARS2 | isoleucyl-tRNA synthetase 2, mitochondrial | 478963 |
| ICAM1 | intercellular adhesion molecule 1 | 403975 |
| ID1 | inhibitor of DNA binding 1, dominant negative helix-loop-helix protein | 609779 |
| ID3 | inhibitor of DNA binding 3, dominant negative helix-loop-helix protein | 403547 |
| ID4 | inhibitor of DNA binding 4, HLH protein | 610666 |
| IDE | insulin-degrading enzyme | 477768 |
| IDH2 | isocitrate dehydrogenase 2 (NADP+), mitochondrial | 479043 |
| IDH3B | isocitrate dehydrogenase 3 (NAD+) beta | 477177 |
| IDH3G | isocitrate dehydrogenase 3 (NAD+) gamma | 481081 |
| IDO1 | indoleamine 2,3-dioxygenase 1 | 475574 |
| IDS | iduronate 2-sulfatase | 492194 |
| IER2 | immediate early response 2 | 484917 |
| IER3 | immediate early response 3 | 481708 |
| IFI44 | interferon induced protein 44 | 490198 |
| IFITM2 | interferon-induced transmembrane protein 1 | 483397 |
| IFNAR1 | interferon (alpha, beta and omega) receptor 1 | 609830 |
| IFNGR1 | interferon gamma receptor 1 | 476216 |
| IFRD1 | interferon-related developmental regulator 1 | 482408 |
| IFT57 | intraflagellar transport 57 | 478553 |
| IGF1 | insulin like growth factor 1 | 610255 |
| IGFBP2 | insulin-like growth factor binding protein 2 | 488516 |
| IGFBP3 | insulin-like growth factor binding protein 3 | 100855619 |
| IGFBP5 | insulin-like growth factor binding protein 5 | 610316 |
| IGSF1 | immunoglobulin superfamily, member 1 | 481051 |
| IGSF11 | immunoglobulin superfamily, member 11 | 487989 |
| IL13RA1 | interleukin 13 receptor, alpha 1 | 403623 |
| IL17RD | interleukin 17 receptor D | 484719 |
| IL18 | interleukin 18 | 403796 |
| IL1A | interleukin 1 alpha | 403782 |
| IL1B | interleukin 1 beta | 403974 |
| IL1R1 | interleukin 1 receptor type 1 | 481328 |
| IL33 | interleukin 33 | 403810 |
| IL6 | interleukin 6 | 403985 |
| IL6ST | interleukin 6 signal transducer | 403545 |
| IMPA2 | inositol(myo)-1(or 4)-monophosphatase 2 | 608801 |
| IMPACT | impact RWD domain protein | 490513 |
| ING3 | inhibitor of growth family, member 3 | 475299 |
| INSL6 | insulin like 6 | 476343 |
| INSR | insulin receptor | 484990 |
| INTS8 | integrator complex subunit 8 | 487056 |
| INTU | inturned planar cell polarity protein | 607233 |
| INVS | inversin | 442950 |
| IPO8 | importin 8 | 612863 |
| IQCE | IQ motif containing E | 489889 |
| IQGAP1 | IQ motif containing GTPase activating protein 1 | 479050 |
| IQGAP3 | IQ motif containing GTPase activating protein 3 | 490412 |
| IRAK2 | interleukin-1 receptor-associated kinase 2 | 484657 |
| IRF1 | interferon regulatory factor 1 | 481500 |
| IRF2BP2 | interferon regulatory factor 2 binding protein 2 | 488961 |
| IRF3 | interferon regulatory factor 3 | 476412 |
| IRF8 | interferon regulatory factor 8 | 489673 |
| ISCU | iron-sulfur cluster assembly enzyme | 477527 |
| ISLR | immunoglobulin superfamily containing leucine-rich repeat | 487643 |
| ITGA6 | integrin, alpha 6 | 478800 |
| ITGA7 | integrin, alpha 7 | 481097 |
| ITGAV | integrin, alpha V | 488437 |
| ITGB1BP1 | integrin beta 1 binding protein 1 | 475660 |
| ITGB2 | integrin subunit beta 2 | 403770 |
| ITGB5 | integrin, beta 5 | 608977 |
| ITGB8 | integrin, beta 8 | 475253 |
| ITIH3 | inter-alpha-trypsin inhibitor heavy chain H3 | 491368 |
| ITIH5 | inter-alpha-trypsin inhibitor heavy chain family member 5 | 100856779 |
| ITM2B | integral membrane protein 2B | 476916 |
| ITM2C | integral membrane protein 2C | 486156 |
| ITPKB | inositol 1,4,5-trisphosphate 3-kinase B | 490383 |
| ITPKC | inositol 1,4,5-trisphosphate 3-kinase C | 612489 |
| ITSN1 | intersectin 1 (SH3 domain protein) | 478409 |
| IVD | isovaleryl Coenzyme A dehydrogenase | 478259 |
| JAK1 | Janus kinase 1 | 442952 |
| JAM2 | junctional adhesion molecule 2 | 478392 |
| JUB | ajuba LIM protein | 480244 |
| JUN | jun oncogene | 609429 |
| JUNB | jun B proto-oncogene | 484927 |
| KALRN | kalirin, RhoGEF kinase | 478592 |
| KBTBD11 | kelch repeat and BTB (POZ) domain containing 11 | 491445 |
| KCMF1 | potassium channel modulatory factor 1 | 475773 |
| KCND1 | potassium voltage-gated channel, Shal-related subfamily, member 1 | 612548 |
| KCND3 | potassium voltage-gated channel subfamily D member 3 | 403758 |
| KCNE1L | KCNE1-like | 610432 |
| KCNG4 | potassium voltage-gated channel, subfamily G, member 4 | 489683 |
| KCNIP1 | Kv channel interacting protein 1 | 489125 |
| KCNIP3 | Kv channel interacting protein 3, calsenilin | 609135 |
| KCNJ10 | potassium inwardly-rectifying channel, subfamily J, member 10 | 488635 |
| KCNJ16 | potassium voltage-gated channel subfamily J member 16 | 611045 |
| KCNK1 | potassium two pore domain channel subfamily K member 1 | 488965 |
| KCNK2 | potassium channel, subfamily K, member 2 | 490295 |
| KCNMB1 | potassium calcium-activated channel subfamily M regulatory beta subunit 1 | 403983 |
| KCNN2 | potassium calcium-activated channel subfamily N member 2 | 474640 |
| KCNN4 | potassium calcium-activated channel subfamily N member 4 | 484464 |
| KCNU1 | potassium channel, subfamily U, member 1 | 482856 |
| KCTD1 | potassium channel tetramerisation domain containing 1 | 480170 |
| KCTD14 | potassium channel tetramerisation domain containing 14 | 485170 |
| KCTD15 | potassium channel tetramerisation domain containing 15 | 484595 |
| KCTD5 | potassium channel tetramerisation domain containing 5 | 490058 |
| KDELC2 | KDEL (Lys-Asp-Glu-Leu) containing 2 | 489419 |
| KDR | kinase insert domain receptor | 482154 |
| KIF11 | kinesin family member 11 | 477769 |
| KIF1A | kinesin family member 1A | 486202 |
| KIF1B | kinesin family member 1B | 479605 |
| KIF1C | kinesin family member 1C | 489453 |
| KIF20A | kinesin family member 20A | 474693 |
| KIF2C | kinesin family member 2C | 475382 |
| KIFC3 | kinesin family member C3 | 487259 |
| KLF12 | Kruppel-like factor 12 | 485493 |
| KLF15 | Kruppel-like factor 15 | 609067 |
| KLF3 | Kruppel-like factor 3 | 488836 |
| KLF5 | Kruppel like factor 5 | 612788 |
| KLF6 | Kruppel like factor 6 | 487151 |
| KLHDC8A | kelch domain containing 8A | 488571 |
| KLHL24 | kelch like family member 24 | 478647 |
| KLHL25 | kelch like family member 25 | 488725 |
| KLHL32 | kelch like family member 32 | 481929 |
| KLHL5 | kelch like family member 5 | 612886 |
| KRAS | KRAS proto-oncogene, GTPase | 403871 |
| KRCC1 | lysine-rich coiled-coil 1 | 475759 |
| KREMEN1 | kringle containing transmembrane protein 1 | 486340 |
| KRT10 | keratin 10 | 491006 |
| L2HGDH | L-2-hydroxyglutarate dehydrogenase | 480316 |
| LACE1 | lactation elevated 1 | 481952 |
| LACTB | lactamase, beta | 487588 |
| LACTB2 | lactamase, beta 2 | 486990 |
| LAIR1 | leukocyte-associated immunoglobulin-like receptor 1 | 484308 |
| LAMA2 | laminin, alpha 2 | 484121 |
| LAMB2 | laminin, beta 2 (laminin S) | 476626 |
| LAMC3 | laminin, gamma 3 | 491290 |
| LAMP2 | lysosomal-associated membrane protein 2 | 481037 |
| LAPTM4A | lysosomal protein transmembrane 4 alpha | 475678 |
| LAPTM5 | lysosomal protein transmembrane 5 | 487324 |
| LASP1 | LIM and SH3 protein 1 | 608624 |
| LASS1 | LAG1 homolog, ceramide synthase 1 | 609789 |
| LCAT | lecithin-cholesterol acyltransferase | 479680 |
| LCN2 | lipocalin 2(LCN2) | 491320 |
| LCP2 | lymphocyte cytosolic protein 2(LCP2) | 489126 |
| LDHB | lactate dehydrogenase B | 477675 |
| LECT1 | leukocyte cell derived chemotaxin 1 | 609613 |
| LEPREL2 | prolyl 3-hydroxylase 3 | 100686626 |
| LGALS1 | galectin 1 | 610276 |
| LGALS3 | galectin 3 | 404021 |
| LGI1 | leucine rich glioma inactivated 1 | 477777 |
| LGI4 | leucine rich repeat LGI family member 4 | 484582 |
| LGR4 | leucine-rich repeat-containing G protein-coupled receptor 4 | 476896 |
| LGR6 | leucine-rich repeat-containing G protein-coupled receptor 6 | 490231 |
| LHFP | lipoma HMGIC fusion partner | 485998 |
| LHX2 | LIM homeobox 2 | 491340 |
| LIF | leukemia inhibitory factor | 403449 |
| LIMD1 | LIM domains containing 1 | 484796 |
| LITAF | lipopolysaccharide induced TNF factor | 490004 |
| LIX1 | limb and CNS expressed 1 | 488894 |
| LIX1L | limb and CNS expressed 1 like | 608332 |
| LMAN2 | lectin, mannose-binding 2 | 403938 |
| LMBRD1 | LMBR1 domain containing 1 | 474955 |
| LMCD1 | LIM and cysteine-rich domains 1 | 476545 |
| LMNA | lamin A/C | 480124 |
| LNPEP | leucyl/cystinyl aminopeptidase | 488896 |
| LNX1 | ligand of numb-protein X 1 | 475143 |
| LONP2 | lon peptidase 2, peroxisomal | 478137 |
| LONRF1 | LON peptidase N-terminal domain and ring finger 1 | 482886 |
| LOXL3 | lysyl oxidase-like 3 | 483101 |
| LPHN3 | latrophilin 3 | 482166 |
| LPIN1 | lipin 1 | 475670 |
| LPP | LIM domain containing preferred translocation partner in lipoma | 478670 |
| LRBA | LPS-responsive vesicle trafficking, beach and anchor containing | 475463 |
| LRG1 | leucine rich alpha-2-glycoprotein 1 | 611876 |
| LRIG1 | leucine-rich repeats and immunoglobulin-like domains 1 | 484698 |
| LRP1 | low density lipoprotein-related protein 1 | 481124 |
| LRP10 | low density lipoprotein receptor-related protein 10 | 608002 |
| LRP4 | low density lipoprotein receptor-related protein 4 | 483628 |
| LRP8 | low density lipoprotein receptor-related protein 8 | 489584 |
| LRPAP1 | low density lipoprotein receptor-related protein associated protein 1 | 479072 |
| LRRC2 | leucine rich repeat containing 2 | 476647 |
| LRRC23 | leucine rich repeat containing 23 | 611613 |
| LRRC33 | leucine rich repeat containing 33 | 488032 |
| LRRC59 | leucine rich repeat containing 59 | 491080 |
| LRRC8A | leucine rich repeat containing 8 family, member A | 491309 |
| LRRFIP1 | LRR binding FLII interacting protein 1 | 477419 |
| LRRK2 | leucine-rich repeat kinase 2 | 486608 |
| LRRN3 | leucine rich repeat neuronal 3 | 482406 |
| LSAMP | limbic system-associated membrane protein | 100683255 |
| LSM6 | LSM6 homolog, U6 small nuclear RNA and mRNA degradation associated | 612329 |
| LTBP1 | latent transforming growth factor beta binding protein 1 | 475720 |
| LTBR | lymphotoxin beta receptor | 486728 |
| LUC7L2 | LUC7-like 2 (S. cerevisiae) | 475529 |
| LXN | latexin | 610062 |
| LY6E | lymphocyte antigen 6 complex, locus E | 100683006 |
| LYN | v-yes-1 Yamaguchi sarcoma viral related oncogene homolog | 477886 |
| LYPD6 | LY6/PLAUR domain containing 6 | 608509 |
| LYPLA1 | lysophospholipase I | 609018 |
| LYRM5 | LYR motif containing 5 | 477669 |
| LYSMD2 | LysM, putative peptidoglycan-binding, domain containing 2 | 478308 |
| LZIC | leucine zipper and CTNNBIP1 domain containing | 491440 |
| M6PR | mannose-6-phosphate receptor | 477700 |
| MACF1 | microtubule-actin crosslinking factor 1 | 475321 |
| MAFF | MAF bZIP transcription factor F | 481255 |
| MAGED2 | melanoma antigen family D, 2 | 480934 |
| MAGEH1 | melanoma antigen family H, 1 | 491904 |
| MAGI2 | membrane associated guanylate kinase, WW and PDZ domain containing 2 | 475904 |
| MAGI3 | membrane associated guanylate kinase, WW and PDZ domain containing 3 | 483214 |
| MAMDC2 | MAM domain containing 2 | 608871 |
| MAN2A2 | mannosidase, alpha, class 2A, member 2 | 488745 |
| MANBA | mannosidase, beta A | 487883 |
| MANEA | mannosidase, endo-alpha | 481925 |
| MANSC1 | MANSC domain containing 1 | 611242 |
| MAOA | monoamine oxidase A | 403450 |
| MAOB | monoamine oxidase B | 403451 |
| MAP2K6 | mitogen-activated protein kinase kinase 6 | 480454 |
| MAP3K1 | mitogen-activated protein kinase kinase kinase 1 | 478061 |
| MAP3K12 | mitogen-activated protein kinase kinase kinase 12 | 607170 |
| MAP3K6 | mitogen-activated protein kinase kinase kinase 6 | 487345 |
| MAPK12 | mitogen-activated protein kinase 12 | 607023 |
| MAPK4 | mitogen-activated protein kinase 4 | 491690 |
| MAPK8IP1 | mitogen-activated protein kinase 8 interacting protein 1 | 483640 |
| MAPK8IP3 | mitogen-activated protein kinase 8 interacting protein 3 | 100684766 |
| MAPKAP1 | mitogen-activated protein kinase associated protein 1 | 480724 |
| MAPKAPK3 | mitogen-activated protein kinase-activated protein kinase 3 | 484756 |
| MAPRE2 | microtubule-associated protein, RP/EB family, member 2 | 490487 |
| MASP1 | mannan binding lectin serine peptidase 1 | 488121 |
| MAT2A | methionine adenosyltransferase II, alpha | 475770 |
| MBOAT1 | membrane bound O-acyltransferase domain containing 1 | 488238 |
| MBOAT2 | membrane bound O-acyltransferase domain containing 2 | 475658 |
| MCC | mutated in colorectal cancers | 611208 |
| MCCC1 | methylcrotonoyl-Coenzyme A carboxylase 1 (alpha) | 478645 |
| MCCC2 | methylcrotonoyl-Coenzyme A carboxylase 2 (beta) | 478091 |
| MCEE | methylmalonyl CoA epimerase | 479018 |
| MCL1 | BCL2 family apoptosis regulator | 403537 |
| MCM3 | minichromosome maintenance complex component 3 | 481839 |
| MCM4 | minichromosome maintenance complex component 4 | 477871 |
| MCM5 | minichromosome maintenance complex component 5 | 610519 |
| MDFIC | MyoD family inhibitor domain containing | 612042 |
| MED12 | mediator complex subunit 12 | 480952 |
| MED25 | mediator complex subunit 25 | 484374 |
| MEGF10 | multiple EGF-like-domains 10 | 474665 |
| MEIS1 | similar to Homeobox protein Meis1 | 474619 |
| MERTK | c-mer proto-oncogene tyrosine kinase | 483060 |
| MEST | mesoderm specific transcript | 607717 |
| MET | MET proto-oncogene, receptor tyrosine kinase | 403438 |
| METRNL | meteorin, glial cell differentiation regulator-like | 608424 |
| METT5D1 | methyltransferase 5 domain containing 1 | 476897 |
| METTL5 | methyltransferase like 5 | 478788 |
| METTL9 | methyltransferase like 9 | 479809 |
| MFAP3 | microfibrillar-associated protein 3 | 489166 |
| MFAP3L | microfibrillar-associated protein 3-like | 477356 |
| MFGE8 | milk fat globule-EGF factor 8 protein | 488730 |
| MFHAS1 | malignant fibrous histiocytoma amplified sequence 1 | 475604 |
| MFN1 | mitofusin 1 | 488086 |
| MFNG | MFNG O-fucosylpeptide 3-beta-N-acetylglucosaminyltransferase | 610303 |
| MFSD1 | major facilitator superfamily domain containing 1 | 488129 |
| MFSD11 | major facilitator superfamily domain containing 11 | 483336 |
| MGAT5 | alpha-1,6-mannosylglycoprotein 6-beta-N-acetylglucosaminyltransferase | 483895 |
| MGLL | monoglyceride lipase | 476511 |
| MGST1 | microsomal glutathione S-transferase 1 | 477683 |
| MGST2 | microsomal glutathione S-transferase 2 | 476078 |
| MIB1 | mindbomb E3 ubiquitin protein ligase 1 | 490521 |
| MICAL1 | microtubule associated monoxygenase, calponin and LIM domain containing 1 | 481958 |
| MICALL2 | MICAL-like 2 | 489898 |
| MID1 | midline 1 | 491737 |
| MID1IP1 | MID1 interacting protein 1 | 491830 |
| MID2 | midline 2 | 481012 |
| MIER1 | MIER1 transcriptional regulator | 479536 |
| MKLN1 | muskelin 1 | 607643 |
| MKS1 | Meckel syndrome, type 1 | 491110 |
| MLC1 | megalencephalic leukoencephalopathy with subcortical cysts 1 | 607105 |
| MLL3 | myeloid/lymphoid or mixed-lineage leukemia 3 | 482810 |
| MMACHC | methylmalonic aciduria (cobalamin deficiency) cblC type, with homocystinuria | 482514 |
| MMD | monocyte to macrophage differentiation-associated | 480562 |
| MMD2 | monocyte to macrophage differentiation-associated 2 | 609327 |
| MMP14 | matrix metallopeptidase 14 | 403823 |
| MOBKL1A | MOB kinase activator 1B | 482187 |
| MOSPD3 | motile sperm domain containing 3 | 608423 |
| MPP6 | membrane palmitoylated protein 6 | 482362 |
| MPST | mercaptopyruvate sulfurtransferase | 474515 |
| MPV17 | MpV17 mitochondrial inner membrane protein | 611056 |
| MR1 | major histocompatibility complex, class I-related | 100686784 |
| MRAS | muscle RAS oncogene homolog | 477082 |
| MRO | maestro | 483979 |
| MRPL13 | mitochondrial ribosomal protein L13 | 482029 |
| MRPL22 | mitochondrial ribosomal protein L22 | 479320 |
| MRPL28 | mitochondrial ribosomal protein L28 | 479897 |
| MRPL35 | mitochondrial ribosomal protein L35 | 612007 |
| MRPS14 | mitochondrial ribosomal protein S14 | 480061 |
| MRPS35 | mitochondrial ribosomal protein S35 | 477660 |
| MRPS6 | mitochondrial ribosomal protein S6 | 106558089 |
| MSI1 | musashi RNA binding protein 1 | 611488 |
| MSI2 | musashi RNA binding protein 2 | 475148 |
| MSN | moesin | 491924 |
| MSRB2 | methionine sulfoxide reductase B2 | 608357 |
| MSX1 | msh homeobox 1 | 488828 |
| MT1 | metallothionein 1F | 403800 |
| MT2 | metallothionein 1H | 403768 |
| MT3 | metallothionein 3 | 611278 |
| MTFR1 | mitochondrial fission regulator 1 | 477898 |
| MTHFD1 | methylenetetrahydrofolate dehydrogenase, cyclohydrolase and formyltetrahydrofolate synthetase 1 | 480352 |
| MTHFD2 | methylenetetrahydrofolate dehydrogenase (NADP+ dependent) 2, methenyltetrahydrofolate cyclohydrolase | 483107 |
| MTM1 | myotubularin 1 | 612385 |
| MTMR11 | myotubularin related protein 11 | 608700 |
| MTX3 | metaxin 3 | 488931 |
| MUM1L1 | melanoma associated antigen (mutated) 1-like 1 | 610246 |
| MUT | methylmalonyl Coenzyme A mutase | 474930 |
| MUTED | biogenesis of lysosomal organelles complex 1 subunit 5 | 609473 |
| MXD4 | MAX dimerization protein 4 | 488803 |
| MXI1 | MAX interactor 1 | 609946 |
| MXRA8 | matrix-remodelling associated 8 | 489592 |
| MYBL1 | MYB proto-oncogene like 1 | 486979 |
| MYC | MYC proto-oncogene, bHLH transcription factor | 403924 |
| MYH11 | myosin, heavy chain 11 | 479836 |
| MYH14 | myosin heavy chain 14 | 100688336 |
| MYH8 | myosin, heavy chain 8 | 403808 |
| MYH9 | myosin heavy chain 9 | 481280 |
| MYL9 | myosin light chain 9 | 485856 |
| MYO10 | myosin X | 489261 |
| MYO6 | myosin VI | 481884 |
| MYOC | myocilin, trabecular meshwork inducible glucocorticoid response | 490344 |
| MYOM3 | myomesin family, member 3 | 487372 |
| MYRIP | myosin VIIA and Rab interacting protein | 485603 |
| NADK | NAD kinase | 489589 |
| NAGA | N-acetylgalactosaminidase, alpha- | 481226 |
| NAPRT1 | nicotinate phosphoribosyltransferase domain containing 1 | 609412 |
| NAT6 | N-acetyltransferase 6 | 608612 |
| NAT8L | N-acetyltransferase 8-like | 612955 |
| NAV2 | neuron navigator 2 | 485411 |
| NAV3 | neuron navigator 3 | 482577 |
| NBEAL1 | similar to neurobeachin-like 1 | 488482 |
| NBN | nibrin | 611315 |
| NCAM1 | neural cell adhesion molecule 1 | 479435 |
| NCAN | neurocan | 484808 |
| NCAPD2 | non-SMC condensin I complex, subunit D2 | 477715 |
| NCF1 | neutrophil cytosolic factor 1 | 607700 |
| NCOA1 | nuclear receptor coactivator 1 | 475684 |
| NCOR1 | nuclear receptor co-repressor 1 | 479515 |
| NDE1 | nudE neurodevelopment protein 1 | 100684378 |
| NDRG2 | NDRG family member 2 | 609390 |
| NDST1 | N-deacetylase and N-sulfotransferase 1 | 489185 |
| NDUFA12 | NADH dehydrogenase (ubiquinone) 1 alpha subcomplex, 12 | 475428 |
| NDUFS4 | NADH:ubiquinone oxidoreductase subunit S4 | 479335 |
| NEBL | nebulette | 477974 |
| NEDD9 | neural precursor cell expressed, developmentally down-regulated 9 | 488220 |
| NEK1 | NIMA (never in mitosis gene a)-related kinase 1 | 486058 |
| NEK7 | NIMA (never in mitosis gene a)-related kinase 7 | 480008 |
| NEK8 | NIMA (never in mitosis gene a)- related kinase 8 | 491171 |
| NEK9 | NIMA (never in mitosis gene a)- related kinase 9 | 490790 |
| NEO1 | neogenin 1 | 487635 |
| NES | nestin | 490410 |
| NETO2 | neuropilin and tolloid like 2 | 478142 |
| NFASC | neurofascin | 488565 |
| NFATC1 | nuclear factor of activated T-cells, cytoplasmic, calcineurin-dependent 1 | 483925 |
| NFE2L2 | nuclear factor (erythroid-derived 2)-like 2 | 478813 |
| NFIA | nuclear factor I/A | 479552 |
| NFIC | nuclear factor I/C (CCAAT-binding transcription factor) | 485061 |
| NFIX | nuclear factor I/X (CCAAT-binding transcription factor) | 484920 |
| NFKBIZ | nuclear factor of kappa light polypeptide gene enhancer in B-cells inhibitor, zeta | 478549 |
| NFS1 | NFS1, cysteine desulfurase | 477214 |
| NGEF | neuronal guanine nucleotide exchange factor | 486165 |
| NGF | nerve growth factor | 403402 |
| NHP2 | NHP2 ribonucleoprotein | 474644 |
| NHSL1 | NHS-like 1 | 476219 |
| NIP7 | NIP7, nucleolar pre-rRNA processing protein | 479673 |
| NIPA2 | non imprinted in Prader-Willi/Angelman syndrome 2 | 479002 |
| NKD1 | naked cuticle homolog 1 | 487288 |
| NKX2-2 | NK2 homeobox 2 | 485744 |
| NLN | neurolysin | 478081 |
| NLRX1 | NLR family member X1 | 489370 |
| NMB | neuromedin B | 479051 |
| NMNAT1 | nicotinamide nucleotide adenylyltransferase 1 | 479601 |
| NNT | nicotinamide nucleotide transhydrogenase | 479342 |
| NOL4 | nucleolar protein 4 | 490491 |
| NOP58 | NOP58 ribonucleoprotein | 100856234 |
| NOTCH1 | Notch 1 | 480676 |
| NOTCH2 | Notch 2 | 483148 |
| NOTCH3 | Notch 3 | 610439 |
| NPAS3 | neuronal PAS domain protein 3 | 100688457 |
| NPC1 | Niemann-Pick disease, type C1 | 403698 |
| NPL | N-acetylneuraminate pyruvate lyase | 490313 |
| NPR2 | natriuretic peptide receptor 2 | 474762 |
| NPTX1 | neuronal pentraxin 1 | 483356 |
| NPVF | neuropeptide VF precursor | 100682920 |
| NPY | neuropeptide Y(NPY) | 475257 |
| NQO1 | NAD(P)H dehydrogenase, quinone 1 | 610935 |
| NQO2 | NAD(P)H dehydrogenase, quinone 2 | 606932 |
| NR1D2 | nuclear receptor subfamily 1, group D, member 2 | 485643 |
| NR2E1 | nuclear receptor subfamily 2, group E, member 1 | 475017 |
| NR2F1 | nuclear receptor subfamily 2, group F, member 1 | 488908 |
| NR3C1 | nuclear receptor subfamily 3, group C, member 1 | 478047 |
| NR3C2 | nuclear receptor subfamily 3, group C, member 2 | 475461 |
| NR4A1 | nuclear receptor subfamily 4, group A, member 1 | 403897 |
| NRARP | NOTCH-regulated ankyrin repeat protein | 100683070 |
| NRG1 | neuregulin 1 | 482861 |
| NRN1 | neuritin 1 | 612757 |
| NRP1 | neuropilin 1 | 477955 |
| NRP2 | neuropilin 2 | 488487 |
| NRXN1 | neurexin 1 | 474589 |
| NRXN2 | neurexin 2 | 483760 |
| NSDHL | NAD(P) dependent steroid dehydrogenase-like | 481079 |
| NSMAF | neutral sphingomyelinase (N-SMase) activation associated factor | 477892 |
| NSUN4 | NOL1/NOP2/Sun domain family, member 4 | 610446 |
| NSUN5 | NOP2/Sun RNA methyltransferase family member 5 | 479720 |
| NT5C2 | 5'-nucleotidase, cytosolic II | 477809 |
| NT5DC1 | 5'-nucleotidase domain containing 1 | 475036 |
| NT5DC3 | 5'-nucleotidase domain containing 3 | 612224 |
| NT5E | 5'-nucleotidase ecto | 474984 |
| NTF3 | neurotrophin 3 | 486731 |
| NTRK2 | neurotrophic receptor tyrosine kinase 2 | 484147 |
| NTRK3 | neurotrophic tyrosine kinase, receptor, type 3 | 609087 |
| NTSR2 | neurotensin receptor 2 | 482972 |
| NUDT12 | nudix (nucleoside diphosphate linked moiety X)-type motif 12 | 488881 |
| NUDT6 | nudix (nucleoside diphosphate linked moiety X)-type motif 6 | 483841 |
| NUDT7 | nudix (nucleoside diphosphate linked moiety X)-type motif 7 | 489703 |
| NUDT9 | nudix (nucleoside diphosphate linked moiety X)-type motif 9 | 478469 |
| NUF2 | NDC80 kinetochore complex component NUF2 | 478988 |
| NUMA1 | nuclear mitotic apparatus protein 1 | 485213 |
| NUMB | NUMB, endocytic adaptor protein | 480381 |
| NUP37 | nucleoporin 37kDa | 475445 |
| NUP93 | nucleoporin 93kDa | 478119 |
| NUP98 | nucleoporin 98kDa | 476822 |
| NUPR1 | nuclear protein, transcriptional regulator, 1 | 479793 |
| NUSAP1 | nucleolar and spindle associated protein 1 | 475154 |
| NWD1 | NACHT and WD repeat domain containing 1 | 476678 |
| NXN | nucleoredoxin | 611162 |
| NXT2 | nuclear transport factor 2-like export factor 2 | 612945 |
| OAF | out at first homolog | 489361 |
| OAT | ornithine aminotransferase | 477858 |
| OCIAD2 | OCIA domain containing 2 | 100688134 |
| OCRL | oculocerebrorenal syndrome of Lowe | 492123 |
| ODC1 | ornithine decarboxylase 1 | 475666 |
| ODZ3 | teneurin transmembrane protein 3 | 475637 |
| OGN | osteoglycin | 610704 |
| OGT | O-linked N-acetylglucosamine (GlcNAc) transferase | 480955 |
| OLFM1 | olfactomedin 1 | 480685 |
| OLFML1 | olfactomedin-like 1 | 476839 |
| OLFML3 | olfactomedin like 3 | 483126 |
| OPLAH | 5-oxoprolinase (ATP-hydrolysing) | 482085 |
| OSBPL9 | oxysterol binding protein-like 9 | 475355 |
| OSGEPL1 | O-sialoglycoprotein endopeptidase-like 1 | 478839 |
| OSGIN2 | oxidative stress induced growth inhibitor family member 2 | 487038 |
| OSMR | oncostatin M receptor | 489223 |
| OSTM1 | osteopetrosis associated transmembrane protein 1 | 612165 |
| OTOS | otospiralin | 477428 |
| OTUD1 | OTU domain containing 1 | 487102 |
| OTX1 | orthodenticle homeobox 1 | 610347 |
| OTX2 | orthodenticle homeobox 2 | 490708 |
| P2RX7 | purinergic receptor P2X, ligand-gated ion channel, 7 | 448778 |
| P4HA1 | prolyl 4-hydroxylase, alpha polypeptide I | 479242 |
| P4HA2 | prolyl 4-hydroxylase, alpha polypeptide II | 474672 |
| P4HA3 | prolyl 4-hydroxylase, alpha polypeptide III | 609381 |
| PABPC1 | poly(A) binding protein, cytoplasmic 1 | 612751 |
| PACRG | PARK2 co-regulated | 484071 |
| PACSIN3 | protein kinase C and casein kinase substrate in neurons 3 | 475984 |
| PADI2 | peptidyl arginine deiminase 2 | 487414 |
| PAFAH2 | platelet-activating factor acetylhydrolase 2 | 478174 |
| PAG1 | phosphoprotein associated with glycosphingolipid microdomains 1 | 610895 |
| PAICS | phosphoribosylaminoimidazole carboxylase and phosphoribosylaminoimidazole succinocarboxamide synthase | 100855769 |
| PAIP1 | poly(A) binding protein interacting protein 1 | 479343 |
| PALLD | palladin, cytoskeletal associated protein | 477351 |
| PANK1 | pantothenate kinase 1 | 486790 |
| PANK2 | pantothenate kinase 2 | 477168 |
| PAPPA | pappalysin 1 | 481692 |
| PAPSS2 | 3'-phosphoadenosine 5'-phosphosulfate synthase 2 | 486463 |
| PAQR3 | progestin and adipoQ receptor family member III | 487820 |
| PAQR4 | progestin and adipoQ receptor family member IV | 490051 |
| PAQR7 | progestin and adipoQ receptor family member VII | 487364 |
| PAQR8 | progestin and adipoQ receptor family member 8 | 100855760 |
| PARP3 | poly (ADP-ribose) polymerase family, member 3 | 484745 |
| PARP9 | poly (ADP-ribose) polymerase family, member 9 | 488010 |
| PARVA | parvin, alpha | 476859 |
| PAX3 | paired box 3 | 488544 |
| PAX6 | paired box 6 | 483441 |
| PBK | PDZ binding kinase | 477371 |
| PBX1 | pre-B-cell leukemia homeobox 1 | 488669 |
| PBX3 | PBX homeobox 3 | 480723 |
| PBXIP1 | pre-B-cell leukemia homeobox interacting protein 1 | 480133 |
| PCBD2 | pterin-4 alpha-carbinolamine dehydratase 2 | 609970 |
| PCBP3 | poly(rC) binding protein 3 | 607066 |
| PCCA | propionyl Coenzyme A carboxylase, alpha polypeptide | 476975 |
| PCCB | propionyl Coenzyme A carboxylase, beta polypeptide | 477076 |
| PCDH17 | protocadherin 17 | 100688591 |
| PCDH20 | protocadherin 20 | 485478 |
| PCDHGC3 | protocadherin gamma-C5 | 478038 |
| PCGF5 | polycomb group ring finger 5 | 486794 |
| PCK2 | phosphoenolpyruvate carboxykinase 2 (mitochondrial) | 480255 |
| PCMTD1 | protein-L-isoaspartate (D-aspartate) O-methyltransferase domain containing 1 | 477874 |
| PCP4 | Purkinje cell protein 4 | 610723 |
| PCP4L1 | Purkinje cell protein 4 like 1 | 100687163 |
| PCSK1N | proprotein convertase subtilisin/kexin type 1 inhibitor | 491870 |
| PDCD4 | programmed cell death 4 | 477818 |
| PDE3B | phosphodiesterase 3B | 611730 |
| PDE4B | phosphodiesterase 4B | 479540 |
| PDE8B | phosphodiesterase 8B | 488939 |
| PDE9A | phosphodiesterase 9A | 611022 |
| PDGFA | platelet derived growth factor subunit A | 491597 |
| PDGFB | platelet derived growth factor subunit B | 442986 |
| PDGFC | platelet derived growth factor C | 482666 |
| PDGFD | platelet derived growth factor D | 479460 |
| PDGFRB | platelet-derived growth factor receptor, beta polypeptide | 442985 |
| PDGFRL | platelet-derived growth factor receptor-like | 607033 |
| PDHA1 | pyruvate dehydrogenase (lipoamide) alpha 1 | 480858 |
| PDK2 | pyruvate dehydrogenase kinase, isozyme 2 | 491075 |
| PDK4 | pyruvate dehydrogenase kinase, isozyme 4 | 482310 |
| PDLIM5 | PDZ and LIM domain 5 | 478482 |
| PDPN | podoplanin | 403886 |
| PDYN | prodynorphin | 485808 |
| PDZD11 | PDZ domain containing 11 | 491940 |
| PDZD2 | PDZ domain containing 2 | 479373 |
| PEA15 | phosphoprotein enriched in astrocytes 15 | 610113 |
| PECI | peroxisomal D3,D2-enoyl-CoA isomerase | 478706 |
| PELI2 | pellino E3 ubiquitin protein ligase family member 2 | 106559103 |
| PER1 | period circadian regulator 1 | 489488 |
| PER2 | period circadian regulator 2 | 486180 |
| PER3 | period circadian regulator 3 | 608256 |
| PEX11A | peroxisomal biogenesis factor 11 alpha | 488736 |
| PEX13 | peroxisomal biogenesis factor 13 | 474607 |
| PEX16 | peroxisomal biogenesis factor 16 | 483639 |
| PEX3 | peroxisomal biogenesis factor 3 | 484015 |
| PEX6 | peroxisomal biogenesis factor 6 | 481805 |
| PEX7 | peroxisomal biogenesis factor 7 | 484000 |
| PFAS | phosphoribosylformylglycinamidine synthase | 489490 |
| PFKFB3 | 6-phosphofructo-2-kinase/fructose-2,6-biphosphatase 3 | 487139 |
| PFKFB4 | 6-phosphofructo-2-kinase/fructose-2,6-biphosphatase 4 | 484777 |
| PFKM | phosphofructokinase, muscle | 403849 |
| PFN4 | profilin family, member 4 | 610829 |
| PGCP | plasma glutamate carboxypeptidase | 477946 |
| PGGT1B | protein geranylgeranyltransferase type I, beta subunit | 481440 |
| PGM1 | phosphoglucomutase 1 | 479545 |
| PGM2 | phosphoglucomutase 2 | 479116 |
| PGM3 | phosphoglucomutase 3 | 474981 |
| PHACTR1 | phosphatase and actin regulator 1 | 478723 |
| PHF21A | PHD finger protein 21A | 483637 |
| PHGDH | phosphoglycerate dehydrogenase | 100856197 |
| PHKA1 | phosphorylase kinase, alpha 1 | 491956 |
| PHKB | phosphorylase kinase, beta | 478139 |
| PHKG1 | phosphorylase kinase catalytic subunit gamma 1 | 489784 |
| PHPT1 | phosphohistidine phosphatase 1 | 491245 |
| PHYHD1 | phytanoyl-CoA dioxygenase domain containing 1 | 608889 |
| PHYHIPL | phytanoyl-CoA 2-hydroxylase interacting protein-like | 479214 |
| PI15 | peptidase inhibitor 15 | 487003 |
| PIAS1 | protein inhibitor of activated STAT, 1 | 478350 |
| PIAS3 | protein inhibitor of activated STAT, 3 | 483160 |
| PIGB | phosphatidylinositol glycan anchor biosynthesis class B | 609998 |
| PIGG | phosphatidylinositol glycan anchor biosynthesis, class G | 479136 |
| PIGH | phosphatidylinositol glycan anchor biosynthesis, class H | 611566 |
| PIGM | phosphatidylinositol glycan anchor biosynthesis, class M | 610166 |
| PIGO | phosphatidylinositol glycan anchor biosynthesis, class O | 474754 |
| PIGS | phosphatidylinositol glycan anchor biosynthesis, class S | 491170 |
| PIGV | phosphatidylinositol glycan anchor biosynthesis, class V | 478172 |
| PIGY | phosphatidylinositol glycan anchor biosynthesis, class Y | 487853 |
| PIK3CA | phosphoinositide-3-kinase, catalytic, alpha polypeptide | 488084 |
| PIPOX | pipecolic acid oxidase | 491177 |
| PIR | pirin | 480846 |
| PITPNC1 | phosphatidylinositol transfer protein, cytoplasmic 1 | 610855 |
| PITRM1 | pitrilysin metallopeptidase 1 | 478018 |
| PKD1 | polycystin 1, transient receptor potential channel interacting | 606755 |
| PKD2 | polycystin 2, transient receptor potential cation channel | 487852 |
| PKN2 | protein kinase N2 | 490174 |
| PLA2G3 | phospholipase A2, group III | 486361 |
| PLA2G4A | phospholipase A2 group IVA | 480048 |
| PLA2G5 | phospholipase A2 group V | 478207 |
| PLA2G7 | phospholipase A2 group VII | 403848 |
| PLAGL1 | pleiomorphic adenoma gene-like 1 | 484018 |
| PLCB3 | phospholipase C, beta 3 | 476034 |
| PLCD4 | phospholipase C, delta 4 | 478910 |
| PLCE1 | phospholipase C, epsilon 1 | 486808 |
| PLCG1 | phospholipase C, gamma 1 | 485874 |
| PLCXD1 | phosphatidylinositol specific phospholipase C X domain containing 1 | 608690 |
| PLD2 | phospholipase D2 | 479473 |
| PLEKHA3 | pleckstrin homology domain containing A3 | 488425 |
| PLEKHB1 | pleckstrin homology domain containing B1 | 608340 |
| PLEKHG2 | pleckstrin homology and RhoGEF domain containing G2 | 484509 |
| PLEKHO1 | pleckstrin homology domain containing, family O member 1 | 607927 |
| PLIN4 | perilipin 4 | 491467 |
| PLN | Phospholamban | 414755 |
| PLOD2 | procollagen-lysine, 2-oxoglutarate 5-dioxygenase 2 | 485702 |
| PLP2 | proteolipid protein 2 | 480914 |
| PLXDC1 | plexin domain containing 1 | 491032 |
| PLXDC2 | plexin domain containing 2 | 608632 |
| PLXNB1 | plexin B1 | 476636 |
| PLXNB2 | plexin B2 | 474459 |
| PLXNC1 | plexin C1 | 100685138 |
| PM20D1 | peptidase M20 domain containing 1 | 106557462 |
| PMM1 | phosphomannomutase 1 | 474486 |
| PMP22 | peripheral myelin protein 22 | 479509 |
| PMPCB | peptidase (mitochondrial processing) beta | 475897 |
| PNP | nucleoside phosphorylase | 475393 |
| PNPLA2 | patatin-like phospholipase domain containing 2 | 611403 |
| PNPLA7 | patatin-like phospholipase domain containing 7 | 480663 |
| PNPLA8 | patatin-like phospholipase domain containing 8 | 475880 |
| POFUT1 | protein O-fucosyltransferase 1 | 609881 |
| POLA1 | polymerase (DNA directed), alpha 1, catalytic subunit | 480869 |
| POLE4 | polymerase (DNA-directed), epsilon 4 (p12 subunit) | 483096 |
| POLG | polymerase (DNA directed), gamma | 488732 |
| POLR3H | polymerase (RNA) III (DNA directed) polypeptide H | 607850 |
| POMT1 | protein-O-mannosyltransferase 1 | 608039 |
| POMT2 | protein-O-mannosyltransferase 2 | 480400 |
| PON2 | paraoxonase 2 | 403855 |
| POU2F1 | POU class 2 homeobox 1 | 490360 |
| POU3F2 | POU class 3 homeobox 2 | 481931 |
| POU3F4 | POU class 3 homeobox 4 | 491988 |
| PPAP2A | phosphatidic acid phosphatase type 2A | 607962 |
| PPAP2B | phospholipid phosphatase 3 | 479557 |
| PPAPDC1A | phosphatidic acid phosphatase type 2 domain containing 1A | 477846 |
| PPARA | peroxisome proliferator-activated receptor alpha | 403654 |
| PPARGC1A | peroxisome proliferator-activated receptor gamma, coactivator 1 alpha | 479127 |
| PPFIA1 | PTPRF interacting protein alpha 1 | 475999 |
| PPIC | peptidylprolyl isomerase C | 481480 |
| PPIG | peptidylprolyl isomerase G | 607519 |
| PPIL6 | peptidylprolyl isomerase like 6 | 612243 |
| PPM1B | protein phosphatase, Mg2+/Mn2+ dependent 1B | 474573 |
| PPM1K | protein phosphatase, Mg2+/Mn2+ dependent 1K | 478473 |
| PPM1M | protein phosphatase, Mg2+/Mn2+ dependent 1M | 484739 |
| PPOX | protoporphyrinogen oxidase | 478980 |
| PPP1R15B | protein phosphatase 1, regulatory (inhibitor) subunit 15B | 488563 |
| PPP1R1A | protein phosphatase 1, regulatory (inhibitor) subunit 1A | 403605 |
| PPP1R3C | protein phosphatase 1 regulatory subunit 3C | 477766 |
| PPP1R3D | protein phosphatase 1, regulatory (inhibitor) subunit 3D | 485953 |
| PPP2R2B | protein phosphatase 2, regulatory subunit B, beta isoform | 478053 |
| PPP2R5A | protein phosphatase 2, regulatory subunit B', alpha isoform | 490280 |
| PPP3R2 | protein phosphatase 3 (formerly 2B), regulatory subunit B, beta isoform | 610453 |
| PPT1 | palmitoyl-protein thioesterase 1 | 475316 |
| PPTC7 | PTC7 protein phosphatase homolog | 100855437 |
| PRC1 | protein regulator of cytokinesis 1 | 488742 |
| PRDM16 | PR domain containing 16 | 479582 |
| PRDM5 | PR domain containing 5 | 483850 |
| PRDX1 | peroxiredoxin 1 | 475375 |
| PRDX4 | peroxiredoxin 4 | 491776 |
| PRDX6 | peroxiredoxin 6 | 480069 |
| PRELP | proline/arginine-rich end leucine-rich repeat protein | 488561 |
| PRKAG1 | protein kinase, AMP-activated, gamma 1 non-catalytic subunit | 486559 |
| PRKCA | protein kinase C alpha | 490904 |
| PRKCD | protein kinase C, delta | 494005 |
| PRKCDBP | protein kinase C, delta binding protein | 476831 |
| PRKD1 | protein kinase D1 | 609091 |
| PRKRA | protein activator of interferon induced protein kinase EIF2AK2 | 488423 |
| PRNP | prion protein | 485783 |
| PROCR | protein C receptor | 485848 |
| PRODH | proline dehydrogenase (oxidase) 1 | 477562 |
| PROS1 | protein S (alpha) | 478529 |
| PRPF19 | pre-mRNA processing factor 19 | 611552 |
| PRPS1 | phosphoribosyl pyrophosphate synthetase 1 | 481011 |
| PRRX1 | paired related homeobox 1 | 609105 |
| PRSS23 | protease, serine 23 | 485150 |
| PSAP | prosaposin | 479240 |
| PSAT1 | phosphoserine aminotransferase 1 | 476318 |
| PSD2 | pleckstrin and Sec7 domain containing 2 | 487157 |
| PSMB8 | proteasome subunit beta 8 | 474865 |
| PSMB9 | proteasome subunit beta 9 | 474867 |
| PSMC3IP | PSMC3 interacting protein | 607659 |
| PSMF1 | proteasome inhibitor subunit 1 | 477184 |
| PSPH | phosphoserine phosphatase | 489783 |
| PTAR1 | protein prenyltransferase alpha subunit repeat containing 1 | 484173 |
| PTCH1 | patched 1 | 484137 |
| PTCH2 | patched 2 | 482519 |
| PTCHD2 | patched domain containing 2 | 608490 |
| PTDSS2 | phosphatidylserine synthase 2 | 483401 |
| PTEN | phosphatase and tensin homolog | 403832 |
| PTER | phosphotriesterase related | 477994 |
| PTGER4 | prostaglandin E receptor 4 | 403589 |
| PTGFRN | prostaglandin F2 receptor negative regulator | 475809 |
| PTGS1 | prostaglandin-endoperoxide synthase 1 | 403544 |
| PTGS2 | prostaglandin-endoperoxide synthase 2 | 442942 |
| PTN | pleiotrophin | 475509 |
| PTPLAD1 | protein tyrosine phosphatase-like A domain containing 1 | 478345 |
| PTPLAD2 | protein tyrosine phosphatase-like A domain containing 2 | 611337 |
| PTPLB | 3-hydroxyacyl-CoA dehydratase 2 | 608764 |
| PTPMT1 | protein tyrosine phosphatase, mitochondrial 1 | 483622 |
| PTPN11 | protein tyrosine phosphatase, non-receptor type 11 | 477488 |
| PTPN12 | protein tyrosine phosphatase, non-receptor type 12 | 483277 |
| PTPN13 | protein tyrosine phosphatase, non-receptor type 13 | 403662 |
| PTPN2 | protein tyrosine phosphatase, non-receptor type 2 | 490563 |
| PTPN21 | protein tyrosine phosphatase, non-receptor type 21 | 490823 |
| PTPN6 | protein tyrosine phosphatase, non-receptor type 6 | 486717 |
| PTPN9 | protein tyrosine phosphatase, non-receptor type 9 | 612991 |
| PTPRA | protein tyrosine phosphatase, receptor type, A | 477176 |
| PTPRF | protein tyrosine phosphatase, receptor type, F | 475389 |
| PTPRG | protein tyrosine phosphatase, receptor type, G | 484706 |
| PTPRT | protein tyrosine phosphatase, receptor type, T | 485878 |
| PTPRZ1 | protein tyrosine phosphatase, receptor type Z1 | 482428 |
| PTS | 6-pyruvoyltetrahydropterin synthase | 611326 |
| PTTG1IP | pituitary tumor-transforming 1 interacting protein | 100856547 |
| PTX3 | pentraxin 3 | 100685178 |
| PURA | purine-rich element binding protein A | 487159 |
| PVR | poliovirus receptor | 476439 |
| PXMP2 | peroxisomal membrane protein 2 | 486222 |
| PXMP4 | peroxisomal membrane protein 4 | 610008 |
| PYCARD | PYD and CARD domain containing | 100856347 |
| PYCRL | pyrroline-5-carboxylate reductase-like | 482079 |
| PYGB | phosphorylase, glycogen; brain | 477003 |
| PYGL | phosphorylase, glycogen, liver | 403738 |
| PYGM | phosphorylase, glycogen, muscle | 611078 |
| QPCTL | glutaminyl-peptide cyclotransferase-like | 484437 |
| QSER1 | glutamine and serine rich 1 | 483436 |
| RAB11B | RAB11B, member RAS oncogene family | 611619 |
| RAB13 | RAB13, member RAS oncogene family | 612294 |
| RAB2 | RAB2A, member RAS oncogene family | 404009 |
| RAB22A | RAB22A, member RAS oncogene family | 403864 |
| RAB30 | RAB30, member RAS oncogene family | 476787 |
| RAB34 | RAB34, member RAS oncogene family | 612952 |
| RAB5A | RAB5A, member RAS oncogene family | 404008 |
| RAB7L1 | RAB7, member RAS oncogene family-like 1 | 478945 |
| RABEP1 | rabaptin, RAB GTPase binding effector protein 1 | 489451 |
| RABGEF1 | RAB guanine nucleotide exchange factor (GEF) 1 | 479706 |
| RABL4 | RAB, member of RAS oncogene family-like 4 | 474517 |
| RAD51 | RAD51 recombinase | 403568 |
| RAMP1 | receptor (G protein-coupled) activity modifying protein 1 | 607163 |
| RAP2B | RAP2B, member of RAS oncogene family | 611828 |
| RAPGEF3 | Rap guanine nucleotide exchange factor (GEF) 3 | 486591 |
| RAPGEF5 | Rap guanine nucleotide exchange factor 5 | 100855786 |
| RARRES2 | retinoic acid receptor responder (tazarotene induced) 2 | 475532 |
| RASA2 | RAS p21 protein activator 2 | 485692 |
| RASL10B | RAS-like, family 10, member B | 491138 |
| RB1 | retinoblastoma 1 | 476915 |
| RBBP9 | retinoblastoma binding protein 9 | 607819 |
| RBL1 | retinoblastoma-like 1 (p107) | 477221 |
| RBL2 | retinoblastoma-like 2 (p130) | 478127 |
| RBP1 | retinol binding protein 1, cellular | 477090 |
| RBPMS2 | RNA binding protein with multiple splicing 2 | 610746 |
| RCBTB2 | RCC1 and BTB domain containing protein 2 | 485446 |
| RCN2 | reticulocalbin 2, EF-hand calcium binding domain | 487666 |
| RDH10 | retinol dehydrogenase 10 | 486998 |
| RDH14 | retinol dehydrogenase 14 | 100856223 |
| RDH5 | retinol dehydrogenase 5 | 481098 |
| RDM1 | RAD52 motif 1 | 480794 |
| RECK | reversion-inducing-cysteine-rich protein with kazal motifs | 403477 |
| REPIN1 | replication initiator 1 | 482792 |
| RETSAT | retinol saturase | 483083 |
| RFX4 | regulatory factor X4 | 474529 |
| RFXANK | regulatory factor X-associated ankyrin-containing protein | 476662 |
| RGL3 | ral guanine nucleotide dissociation stimulator-like 3 | 484942 |
| RGMA | RGM domain family, member A | 488719 |
| RGS1 | regulator of G-protein signaling 1 | 488585 |
| RGS12 | regulator of G-protein signaling 12 | 608720 |
| RGS20 | regulator of G-protein signaling 20 | 486954 |
| RGS6 | regulator of G-protein signaling 6 | 480380 |
| RGS7 | regulator of G-protein signaling 7 | 100687600 |
| RHBDD1 | rhomboid domain containing 1 | 486149 |
| RHBDF1 | rhomboid 5 homolog 1 | 490101 |
| RHCG | Rh family, C glycoprotein | 479040 |
| RHOBTB2 | Rho-related BTB domain containing 2 | 486120 |
| RHOBTB3 | Rho related BTB domain containing 3 | 100687706 |
| RHOC | ras homolog gene family, member C | 483218 |
| RHOJ | ras homolog gene family, member J | 612985 |
| RHOQ | ras homolog gene family, member Q | 474579 |
| RHPN1 | rhophilin, Rho GTPase binding protein 1 | 482076 |
| RIC3 | RIC3 acetylcholine receptor chaperone | 611343 |
| RILP | Rab interacting lysosomal protein | 480649 |
| RIN2 | Ras and Rab interactor 2 | 477139 |
| RIPK1 | receptor (TNFRSF)-interacting serine-threonine kinase 1 | 488195 |
| RLBP1 | retinaldehyde binding protein 1 | 479039 |
| RNASE10 | ribonuclease A family member 10 (inactive) | 482560 |
| RNASET2 | ribonuclease T2 | 612451 |
| RNF103 | ring finger protein 103 | 475761 |
| RNF121 | ring finger protein 121 | 476819 |
| RNF125 | ring finger protein 125 | 490498 |
| RNF139 | ring finger protein 139 | 609422 |
| RNF180 | ring finger protein 180 | 487231 |
| RNF182 | ring finger protein 182 | 488225 |
| RNF19B | ring finger protein 19B | 478146 |
| RNF6 | ring finger protein (C3H2C3 type) 6 | 477332 |
| RNH1 | ribonuclease/angiogenin inhibitor 1 | 483402 |
| ROM1 | retinal outer segment membrane protein 1 | 483786 |
| RORA | RAR-related orphan receptor A | 478328 |
| RORB | RAR-related orphan receptor B | 484164 |
| RPA1 | replication protein A1, 70kDa | 491196 |
| RPA2 | replication protein A2, 32kDa | 487339 |
| RPE65 | RPE65, retinoid isomerohydrolase | 403803 |
| RPGRIP1 | retinitis pigmentosa GTPase regulator interacting protein 1 | 475400 |
| RPL27A | ribosomal protein L27a | 611398 |
| RPP14 | ribonuclease P/MRP 14kDa subunit | 612771 |
| RRAGD | Ras-related GTP binding D | 474994 |
| RREB1 | ras responsive element binding protein 1 | 488206 |
| RRM2 | ribonucleotide reductase M2 polypeptide | 482963 |
| RRS1 | ribosome biogenesis regulator homolog | 486978 |
| RSAD2 | radical S-adenosyl methionine domain containing 2 | 609005 |
| RSBN1 | round spermatid basic protein 1 | 475802 |
| RSPO1 | R-spondin 1 | 608179 |
| RSPO3 | R-spondin 3 | 476287 |
| RSU1 | Ras suppressor protein 1 | 477993 |
| RUFY3 | RUN and FYVE domain containing 3 | 475168 |
| RUNDC1 | RUN domain containing 1 | 490955 |
| RXRA | retinoid X receptor, alpha | 491278 |
| RYK | RYK receptor-like tyrosine kinase | 477074 |
| RYR3 | ryanodine receptor 3 | 100686627 |
| S100A1 | S100 calcium binding protein A1 | 480141 |
| S100A10 | S100 calcium binding protein A10 | 475851 |
| S100A11 | S100 calcium binding protein A11 | 475852 |
| S100A4 | S100 calcium binding protein A4 | 403787 |
| S100A6 | S100 calcium binding protein A6 | 480143 |
| S100A8 | S100 calcium binding protein A8 | 490461 |
| S100A9 | S100 calcium binding protein A9 | 490463 |
| S100B | S100 protein, beta polypeptide | 491615 |
| S1PR3 | sphingosine-1-phosphate receptor 3 | 484199 |
| SACM1L | SAC1 like phosphatidylinositide phosphatase | 484795 |
| SALL1 | spalt like transcription factor 1 | 487284 |
| SALL3 | spalt like transcription factor 3 | 607126 |
| SAP30 | Sin3A-associated protein | 607359 |
| SAP30L | SAP30-like | 612111 |
| SAPS1 | SAPS domain family, member 1 | 484298 |
| SARDH | sarcosine dehydrogenase | 491277 |
| SASH1 | SAM and SH3 domain containing 1 | 608649 |
| SBNO2 | strawberry notch homolog 2 | 485089 |
| SC4MOL | sterol-C4-methyl oxidase-like | 475491 |
| SCARA3 | scavenger receptor class A, member 3 | 486099 |
| SCG3 | secretogranin III | 478307 |
| SCG5 | secretogranin V | 478249 |
| SCN7A | sodium voltage-gated channel alpha subunit 7 | 488382 |
| SCP2 | sterol carrier protein 2 | 479564 |
| SCPEP1 | serine carboxypeptidase 1 | 480566 |
| SCRG1 | stimulator of chondrogenesis 1 | 100686672 |
| SCRN2 | secernin 2 | 491046 |
| SDC2 | syndecan 2 | 477945 |
| SDC4 | syndecan 4 | 485893 |
| SDCBP | syndecan binding protein (syntenin) | 482977 |
| SDF2 | stromal cell-derived factor 2 | 480626 |
| SDHA | succinate dehydrogenase complex, subunit A, flavoprotein (Fp) | 478634 |
| SDHC | succinate dehydrogenase complex subunit C | 478983 |
| SDSL | serine dehydratase-like | 486282 |
| SEC14L1 | SEC14 like lipid binding 1 | 483338 |
| SEC14L2 | SEC14 like lipid binding 2 | 477539 |
| SEC63 | SEC63 homolog, protein translocation regulator | 475016 |
| SELENBP1 | selenium binding protein 1 | 475847 |
| SEMA4A | semaphorin 4A | 490416 |
| SEMA4B | semaphorin 4B | 488741 |
| SEMA4C | semaphorin 4C | 481343 |
| SEMA4D | semaphorin 4D | 476350 |
| SEMA6D | semaphorin 6D | 478291 |
| SEPP1 | selenoprotein P, plasma, 1 | 479346 |
| SEPT11 | septin 11 | 478439 |
| SEPT6 | septin 6 | 612892 |
| SEPT8 | septin 8 | 474677 |
| SEPT9 | septin 9 | 483339 |
| SERPINB9 | serpin peptidase inhibitor, clade B (ovalbumin), member 9 | 488192 |
| SERPINE1 | serpin family E member 1 | 403476 |
| SERPINE2 | serpin family E member 2 | 608930 |
| SERPINF1 | serpin family F member 1 | 611276 |
| SERPINF2 | serpin family F member 2 | 611266 |
| SERPING1 | serpin family G member 1 | 475966 |
| SERPINH1 | serpin family H member 1 | 485187 |
| SESN1 | sestrin 1 | 481956 |
| SESN3 | sestrin 3 | 485118 |
| SETD5 | SET domain containing 5 | 476542 |
| SEZ6 | seizure related 6 homolog | 491175 |
| SFMBT2 | Scm-like with four mbt domains 2 | 608271 |
| SFXN1 | sideroflexin 1 | 489109 |
| SFXN2 | sideroflexin 2 | 486864 |
| SFXN5 | sideroflexin 5 | 612350 |
| SGIP1 | SH3 domain GRB2 like endophilin interacting protein 1 | 100856415 |
| SGO2 | shugoshin 2 | 478863 |
| SGPL1 | sphingosine-1-phosphate lyase 1 | 489032 |
| SH3BGRL | SH3 domain binding glutamic acid-rich protein like | 491986 |
| SH3BP5 | SH3 domain binding protein 5 | 485657 |
| SH3PXD2B | SH3 and PX domains 2B | 489119 |
| SHH | sonic hedgehog | 608860 |
| SHISA6 | shisa family member 6 | 489506 |
| SIAE | sialic acid acetylesterase | 489311 |
| SIL1 | SIL1 nucleotide exchange factor | 474699 |
| SIRPA | signal-regulatory protein alpha | 609452 |
| SIRT3 | sirtuin 3 | 475933 |
| SKAP2 | src kinase associated phosphoprotein 2 | 482366 |
| SKI | SKI proto-oncogene | 489610 |
| SKIV2L2 | Mtr4 exosome RNA helicase | 607950 |
| SKP2 | S-phase kinase-associated protein 2 (p45) | 489228 |
| SLA | Src-like-adaptor | 608259 |
| SLAMF9 | SLAM family member 9 | 100684400 |
| SLC10A6 | solute carrier family 10 member 6 | 609030 |
| SLC11A1 | solute carrier family 11 member 1 | 478909 |
| SLC12A4 | solute carrier family 12 member 4 | 479679 |
| SLC12A7 | solute carrier family 12 member 7 | 488069 |
| SLC12A9 | solute carrier family 12 member 9 | 489829 |
| SLC13A3 | solute carrier family 13 member 3 | 485910 |
| SLC13A5 | solute carrier family 13 member 5 | 606984 |
| SLC14A1 | solute carrier family 14 member 1 | 490470 |
| SLC14A2 | solute carrier family 14 member 2 | 490471 |
| SLC15A2 | solute carrier family 15 member 2 | 488006 |
| SLC15A3 | solute carrier family 15 member 3 | 612351 |
| SLC16A3 | solute carrier family 16 member 3 | 483379 |
| SLC19A2 | solute carrier family 19 member 2 | 490353 |
| SLC1A2 | solute carrier family 1 member 2 | 403750 |
| SLC1A3 | solute carrier family 1 member 3 | 403748 |
| SLC1A4 | solute carrier family 1 member 4 | 481391 |
| SLC1A5 | solute carrier family 1 member 5 | 484425 |
| SLC20A1 | solute carrier family 20 member 1 | 483066 |
| SLC20A2 | solute carrier family 20 member 2 | 482838 |
| SLC22A4 | solute carrier family 22 member 4 | 474673 |
| SLC23A2 | solute carrier family 23 member 2 | 403490 |
| SLC25A16 | solute carrier family 25 member 16 | 489016 |
| SLC25A18 | solute carrier family 25 member 18 | 100856697 |
| SLC25A23 | solute carrier family 25 member 23 | 485020 |
| SLC25A29 | solute carrier family 25 member 29 | 490856 |
| SLC25A33 | solute carrier family 25 member 33 | 479599 |
| SLC25A34 | solute carrier family 25 member 34 | 608629 |
| SLC25A42 | solute carrier family 25 member 42 | 609751 |
| SLC25A5 | solute carrier family 25 member 5 | 492093 |
| SLC26A6 | solute carrier family 26, member 6 | 608998 |
| SLC27A1 | solute carrier family 27 member 1 | 484835 |
| SLC29A2 | solute carrier family 29 member 2 | 611913 |
| SLC2A10 | solute carrier family 2 member 10 | 485911 |
| SLC2A12 | solute carrier family 2 member 12 | 483994 |
| SLC2A8 | solute carrier family 2 member 8 | 480717 |
| SLC30A10 | solute carrier family 30 member 10 | 488599 |
| SLC30A5 | solute carrier family 30 member 5 | 487237 |
| SLC30A6 | solute carrier family 30 member 6 | 475716 |
| SLC30A7 | solute carrier family 30 member 7 | 479924 |
| SLC31A1 | solute carrier family 31 member 1 | 481678 |
| SLC33A1 | solute carrier family 33 member 1 | 485724 |
| SLC35B4 | solute carrier family 35 member B4 | 482249 |
| SLC35F3 | solute carrier family 35 member F3 | 488963 |
| SLC35F5 | solute carrier family 35 member F5 | 476123 |
| SLC36A2 | solute carrier family 36 member 2 | 489174 |
| SLC37A2 | solute carrier family 37 member 2 | 489303 |
| SLC38A3 | solute carrier family 38 member 3 | 476617 |
| SLC39A1 | solute carrier family 39 member 1 | 100855713 |
| SLC39A11 | solute carrier family 39 member 11 | 610065 |
| SLC39A12 | solute carrier family 39 member 12 | 477989 |
| SLC39A13 | solute carrier family 39 member 13 | 475981 |
| SLC39A14 | solute carrier family 39 member 14 | 486124 |
| SLC3A2 | solute carrier family 3 member 2 | 483777 |
| SLC41A1 | solute carrier family 41 member 1 | 478947 |
| SLC43A3 | solute carrier family 43 member 3 | 483494 |
| SLC44A2 | solute carrier family 44 member 2 | 484951 |
| SLC44A3 | solute carrier family 44 member 3 | 490152 |
| SLC4A4 | solute carrier family 4 member 4 | 475171 |
| SLC5A3 | solute carrier family 5 member 3 | 445542 |
| SLC6A1 | solute carrier family 6 member 1 | 484655 |
| SLC6A11 | solute carrier family 6 member 11 | 476534 |
| SLC7A1 | solute carrier family 7 member 1 | 486022 |
| SLC7A10 | solute carrier family 7 member 10 | 484599 |
| SLC7A11 | solute carrier family 7 member 11 | 483821 |
| SLC7A2 | solute carrier family 7 member 2 | 475614 |
| SLC9A3R1 | solute carrier family 9 member 3 regulator 1 | 483299 |
| SLCO1C1 | solute carrier organic anion transporter family, member 1C1 | 609603 |
| SLITRK4 | SLIT and NTRK like family member 4 | 492183 |
| SLMO1 | PRELI domain containing 3A | 490561 |
| SLU7 | SLU7 homolog, splicing factor | 479308 |
| SMAD4 | SMAD family member 4 | 476196 |
| SMAD5 | SMAD family member 5 | 481520 |
| SMARCD2 | SWI/SNF related, matrix associated, actin dependent regulator of chromatin, subfamily d, member 2 | 480477 |
| SMC4 | structural maintenance of chromosomes 4 | 478679 |
| SMC5 | structural maintenance of chromosomes 5 | 476327 |
| SMEK1 | protein phosphatase 4 regulatory subunit 3A | 612974 |
| SMEK2 | protein phosphatase 4 regulatory subunit 3B | 474603 |
| SMO | smoothened, frizzled class receptor | 482262 |
| SMOX | spermine oxidase | 485787 |
| SMPD1 | sphingomyelin phosphodiesterase 1, acid lysosomal | 485334 |
| SMPD2 | sphingomyelin phosphodiesterase 2 | 100856377 |
| SMPD4 | sphingomyelin phosphodiesterase 4 | 486434 |
| SMPDL3B | sphingomyelin phosphodiesterase acid like 3B | 100855784 |
| SNAPC2 | small nuclear RNA activating complex, polypeptide 2 | 611568 |
| SNCAIP | synuclein, alpha interacting protein | 481479 |
| SNED1 | sushi, nidogen and EGF like domains 1 | 609463 |
| SNPH | syntaphilin | 485812 |
| SNRK | SNF related kinase | 477011 |
| SNTA1 | syntrophin alpha 1 | 485837 |
| SNX5 | sorting nexin 5 | 485754 |
| SOAT1 | sterol O-acyltransferase 1 | 490325 |
| SOCS3 | suppressor of cytokine signaling 3 | 442949 |
| SOD1 | superoxide dismutase 1 | 403559 |
| SOD2 | superoxide dismutase 2 | 476258 |
| SOD3 | superoxide dismutase 3, extracellular | 488855 |
| SORBS1 | sorbin and SH3 domain containing 1 | 100686806 |
| SORCS2 | sortilin-related VPS10 domain containing receptor 2 | 488785 |
| SORL1 | sortilin-related receptor, L(DLR class) A repeats-containing | 479408 |
| SOSTDC1 | sclerostin domain containing 1 | 100856691 |
| SOX2 | SRY-box 2 | 488092 |
| SOX21 | SRY-box 21 | 100686255 |
| SOX5 | SRY-box 5 | 486635 |
| SOX7 | SRY-box 7 | 486084 |
| SOX9 | SRY-box 9 | 403464 |
| SP1 | Sp1 transcription factor | 486507 |
| SP5 | Sp5 transcription factor | 609211 |
| SPAG1 | sperm associated antigen 1 | 607063 |
| SPAG16 | sperm associated antigen 16 | 478899 |
| SPAG5 | sperm associated antigen 5 | 480623 |
| SPAG9 | sperm associated antigen 9 | 480557 |
| SPARCL1 | SPARC-like 1 | 478470 |
| SPATA13 | spermatogenesis associated 13 | 486040 |
| SPATA19 | spermatogenesis associated 19 | 479387 |
| SPEG | SPEG complex locus | 478924 |
| SPHK1 | sphingosine kinase 1 | 483329 |
| SPHKAP | SPHK1 interactor, AKAP domain containing | 609866 |
| SPIN1 | spindlin 1 | 476353 |
| SPIRE1 | spire type actin nucleation factor 1 | 490562 |
| SPOCK2 | SPARC (osteonectin), cwcv and kazal like domains proteoglycan 2 | 489037 |
| SPON1 | spondin 1, extracellular matrix protein | 476864 |
| SPP1 | secreted phosphoprotein 1 | 478471 |
| SPR | sepiapterin reductase (7,8-dihydrobiopterin:NADP+ oxidoreductase) | 483118 |
| SPRED1 | sprouty-related, EVH1 domain containing 1 | 487485 |
| SPRED2 | sprouty-related, EVH1 domain containing 2 | 481397 |
| SPRY2 | sprouty RTK signaling antagonist 2 | 485504 |
| SPRY4 | sprouty RTK signaling antagonist 4 | 487192 |
| SPSB4 | splA/ryanodine receptor domain and SOCS box containing 4 | 485691 |
| SQRDL | sulfide quinone oxidoreductase | 478289 |
| SRC | SRC proto-oncogene, non-receptor tyrosine kinase | 485864 |
| SREBF1 | sterol regulatory element binding transcription factor 1 | 403475 |
| SREBF2 | sterol regulatory element binding transcription factor 2 | 481228 |
| SRGAP3 | SLIT-ROBO Rho GTPase activating protein 3 | 610229 |
| SRGN | serglycin | 609421 |
| SRR | serine racemase | 491200 |
| SRXN1 | sulfiredoxin 1 | 100686728 |
| SSFA2 | sperm specific antigen 2 | 478824 |
| SSR1 | signal sequence receptor, alpha | 403951 |
| SSR3 | signal sequence receptor subunit 3 | 477124 |
| SSX2IP | SSX family member 2 interacting protein | 479970 |
| ST3GAL2 | ST3 beta-galactoside alpha-2,3-sialyltransferase 2 | 489714 |
| ST3GAL4 | ST3 beta-galactoside alpha-2,3-sialyltransferase 4 | 607094 |
| ST5 | suppression of tumorigenicity 5 | 485380 |
| ST6GAL1 | ST6 beta-galactosamide alpha-2,6-sialyltranferase 1 | 478668 |
| ST6GALNAC4 | ST6 N-acetylgalactosaminide alpha-2,6-sialyltransferase 4 | 609133 |
| ST6GALNAC5 | ST6 N-acetylgalactosaminide alpha-2,6-sialyltransferase 5 | 613015 |
| ST8SIA2 | ST8 alpha-N-acetyl-neuraminide alpha-2,8-sialyltransferase 2 | 488722 |
| STAG1 | stromal antigen 1 | 485674 |
| STARD6 | StAR-related lipid transfer (START) domain containing 6 | 476193 |
| STARD8 | StAR-related lipid transfer (START) domain containing 8 | 491932 |
| STAT3 | signal transducer and activator of transcription 3 | 490967 |
| STAT5A | signal transducer and activator of transcription 5A | 490968 |
| STAT5B | signal transducer and activator of transcription 5B | 490969 |
| STCH | stress 70 protein chaperone, microsome-associated | 608091 |
| STEAP1 | STEAP family member 1 | 475222 |
| STEAP4 | STEAP4 metalloreductase | 482287 |
| STK17B | serine/threonine kinase 17b | 488453 |
| STK38 | serine/threonine kinase 38 | 481766 |
| STK38L | serine/threonine kinase 38 like | 477663 |
| STK40 | serine/threonine kinase 40 | 482475 |
| STOX1 | storkhead box 1 | 489017 |
| STT3B | STT3B, catalytic subunit of the oligosaccharyltransferase complex | 485628 |
| STX11 | syntaxin 11 | 484019 |
| STXBP4 | syntaxin binding protein 4 | 491094 |
| STXBP6 | syntaxin binding protein 6 | 608615 |
| SUCLG1 | succinate-CoA ligase, alpha subunit | 475775 |
| SUCLG2 | succinate-CoA ligase, GDP-forming, beta subunit | 476562 |
| SUFU | SUFU negative regulator of hedgehog signaling | 608531 |
| SULF1 | sulfatase 1 | 486986 |
| SULF2 | sulfatase 2 | 477254 |
| SULT1A1 | sulfotransferase family, cytosolic, 1A, phenol-preferring, member 1 | 403892 |
| SUMF1 | sulfatase modifying factor 1 | 484681 |
| SUMF2 | sulfatase modifying factor 2 | 479701 |
| SUOX | sulfite oxidase | 481103 |
| SUPV3L1 | Suv3 like RNA helicase | 489018 |
| SUZ12 | SUZ12, polycomb repressive complex 2 subunit | 491158 |
| SWAP70 | SWAP switching B-cell complex 70kDa subunit | 485385 |
| SYDE1 | synapse defective Rho GTPase homolog 1 | 100856120 |
| SYN2 | synapsin II | 484652 |
| SYNE1 | spectrin repeat containing, nuclear envelope 1 | 484037 |
| SYNM | synemin | 479024 |
| SYNPO | synaptopodin | 479327 |
| SYNPO2 | synaptopodin 2 | 611023 |
| SYPL2 | synaptophysin like 2 | 611827 |
| SYT12 | synaptotagmin XII | 483703 |
| SYT13 | synaptotagmin 13 | 483643 |
| SYT4 | synaptotagmin 4 | 490474 |
| TACC1 | transforming acidic coiled-coil containing protein 1 | 475581 |
| TACC3 | transforming, acidic coiled-coil containing protein 3 | 479079 |
| TAF13 | TATA-box binding protein associated factor 13 | 100688743 |
| TAGLN | transgelin | 479424 |
| TAGLN2 | transgelin 2 | 610210 |
| TAGLN3 | transgelin 3 | 478562 |
| TANK | TRAF family member-associated NFKB activator | 608092 |
| TAPBP | TAP binding protein (tapasin) | 481740 |
| TAPBPL | TAP binding protein like | 486727 |
| TATDN3 | TatD DNase domain containing 3 | 490284 |
| TAX1BP1 | Tax1 (human T-cell leukemia virus type I) binding protein 1 | 475264 |
| TBC1D10A | TBC1 domain family, member 10A | 486350 |
| TBC1D12 | TBC1 domain family, member 12 | 612776 |
| TBC1D2B | TBC1 domain family, member 2B | 488776 |
| TBCEL | tubulin folding cofactor E-like | 489358 |
| TBL1X | transducin (beta)-like 1X-linked | 611017 |
| TBXAS1 | thromboxane A synthase 1 | 482771 |
| TCEAL1 | transcription elongation factor A like 1 | 481000 |
| TCF19 | transcription factor 19(TCF19) | 474835 |
| TCF25 | transcription factor 25 | 608053 |
| TCF3 | transcription factor 3 | 485079 |
| TCF4 | transcription factor 4 | 403949 |
| TCN2 | transcobalamin 2 | 486355 |
| TDRD3 | similar to tudor domain containing 3 | 491346 |
| TEAD2 | TEA domain family member 2 | 484381 |
| TECTB | tectorin beta | 486891 |
| TEF | thyrotrophic embryonic factor | 607883 |
| TEKT4 | tektin 4 | 490081 |
| TEX2 | testis expressed 2 | 480474 |
| TEX9 | testis expressed 9 | 487572 |
| TGDS | TDP-glucose 4,6-dehydratase | 485521 |
| TGFB2 | transforming growth factor, beta 2 | 488596 |
| TGFB3 | transforming growth factor, beta 3 | 490796 |
| TGFBI | transforming growth factor beta induced | 481519 |
| TGIF1 | TGFB induced factor homeobox 1 | 490537 |
| TGIF2 | TGFB-induced factor homeobox 2 | 485857 |
| TGM1 | transglutaminase 1 | 403630 |
| TGM2 | transglutaminase 2 | 485867 |
| THAP12 | THAP domain containing 12 | 485182 |
| THBD | thrombomodulin | 474355 |
| THBS1 | thrombospondin 1 | 487486 |
| THBS2 | thrombospondin 2 | 484087 |
| THBS4 | thrombospondin 4 | 488930 |
| THEM5 | thioesterase superfamily member 5 | 608399 |
| THRSP | thyroid hormone responsive | 485169 |
| THUMPD2 | THUMP domain containing 2 | 483044 |
| TICAM1 | toll-like receptor adaptor molecule 1 | 611852 |
| TIMP1 | TIMP metallopeptidase inhibitor 1 | 403816 |
| TIMP3 | TIMP metallopeptidase inhibitor 3 | 481289 |
| TIMP4 | TIMP metallopeptidase inhibitor 4 | 100688494 |
| TIPARP | TCDD-inducible poly(ADP-ribose) polymerase | 485728 |
| TJP1 | tight junction protein 1 | 403752 |
| TJP2 | tight junction protein 2 | 403854 |
| TK1 | thymidine kinase 1, soluble | 483343 |
| TLE1 | transducin like enhancer of split 1 | 476314 |
| TLR2 | toll like receptor 2 | 448807 |
| TLR3 | toll like receptor 3 | 482905 |
| TLR4 | toll like receptor 4 | 403417 |
| TM2D3 | TM2 domain containing 3 | 479020 |
| TM7SF2 | transmembrane 7 superfamily member 2 | 476027 |
| TM9SF1 | transmembrane 9 superfamily member 1 | 480261 |
| TM9SF2 | transmembrane 9 superfamily member 2 | 476972 |
| TMC7 | transmembrane channel-like 7 | 489990 |
| TMCC1 | transmembrane and coiled-coil domain family 1 | 484644 |
| TMCO1 | transmembrane and coiled-coil domains 1 | 478992 |
| TMED10 | transmembrane p24 trafficking protein 10 | 610559 |
| TMED5 | transmembrane p24 trafficking protein 5 | 479947 |
| TMEM100 | transmembrane protein 100 | 609661 |
| TMEM106B | transmembrane protein 106B | 482322 |
| TMEM106C | transmembrane protein 106C | 477630 |
| TMEM109 | transmembrane protein 109 | 612355 |
| TMEM115 | transmembrane protein 115 | 484758 |
| TMEM129 | transmembrane protein 129 | 608955 |
| TMEM135 | transmembrane protein 135 | 607444 |
| TMEM144 | transmembrane protein 144 | 482668 |
| TMEM161B | transmembrane protein 161B | 488916 |
| TMEM164 | transmembrane protein 164 | 609227 |
| TMEM166 | transmembrane protein 166 | 483095 |
| TMEM168 | transmembrane protein 168 | 611975 |
| TMEM176A | transmembrane protein 176A | 475535 |
| TMEM176B | transmembrane protein 176B | 610914 |
| TMEM30A | transmembrane protein 30A | 474973 |
| TMEM33 | transmembrane protein 33 | 612937 |
| TMEM41B | transmembrane protein 41B | 476845 |
| TMEM43 | transmembrane protein 43 | 484637 |
| TMEM47 | transmembrane protein 47 | 403572 |
| TMEM51 | transmembrane protein 51 | 607267 |
| TMEM55A | transmembrane protein 55A | 477939 |
| TMEM56 | transmembrane protein 56 | 490151 |
| TMEM74 | transmembrane protein 74 | 100682800 |
| TMIE | transmembrane inner ear | 609350 |
| TMOD3 | tropomodulin 3 | 487554 |
| TMPO | thymopoietin | 482618 |
| TMTC3 | transmembrane and tetratricopeptide repeat containing 3 | 475421 |
| TMTC4 | transmembrane and tetratricopeptide repeat containing 4 | 100855496 |
| TNC | Tenascin C | 481689 |
| TNF | tumor necrosis factor | 403922 |
| TNFAIP1 | tumor necrosis factor, alpha-induced protein 1 | 491161 |
| TNFAIP2 | tumor necrosis factor, alpha-induced protein 2 | 480440 |
| TNFAIP8 | tumor necrosis factor, alpha-induced protein 8 | 481428 |
| TNFRSF12A | TNF receptor superfamily member 12A | 610734 |
| TNFRSF19 | tumor necrosis factor receptor superfamily, member 19 | 486042 |
| TNFRSF1A | tumor necrosis factor receptor superfamily, member 1A | 403634 |
| TNFSF10 | tumor necrosis factor superfamily member 10 | 100174780 |
| TNIK | TRAF2 and NCK interacting kinase | 488166 |
| TNPO1 | transportin 1 | 478093 |
| TNS1 | tensin 1 | 488519 |
| TOB2 | transducer of ERBB2, 2 | 481233 |
| TOM1 | target of myb1 membrane trafficking protein | 608746 |
| TOM1L1 | target of myb1 like 1 membrane trafficking protein | 491093 |
| TOM1L2 | target of myb1 like 2 membrane trafficking protein | 489540 |
| TOP1 | topoisomerase (DNA) I | 477229 |
| TOP2A | topoisomerase (DNA) II alpha | 480525 |
| TOR1AIP1 | torsin A interacting protein 1 | 610317 |
| TOR1AIP2 | torsin A interacting protein 2 | 610310 |
| TOR2A | torsin family 2, member A | 609191 |
| TOR3A | torsin family 3, member A | 490326 |
| TOX | thymocyte selection-associated high mobility group box | 486964 |
| TPBG | trophoblast glycoprotein | 481899 |
| TPCN1 | two pore segment channel 1 | 477492 |
| TPH2 | tryptophan hydroxylase 2 | 481165 |
| TPMT | thiopurine S-methyltransferase | 403536 |
| TPP1 | tripeptidyl peptidase I | 485337 |
| TPRKB | TP53RK binding protein | 475794 |
| TPST2 | tyrosylprotein sulfotransferase 2 | 486332 |
| TPX2 | TPX2, microtubule nucleation factor | 477186 |
| TRA2A | transformer 2 alpha homolog | 482358 |
| TRAF3 | TNF receptor-associated factor 3 | 490867 |
| TRAF3IP1 | TNF receptor-associated factor 3 interacting protein 1 | 607339 |
| TRAF7 | TNF receptor-associated factor 7 | 609824 |
| TRAK1 | trafficking protein, kinesin binding 1 | 485610 |
| TRAM1 | translocation associated membrane protein 1 | 403948 |
| TRDMT1 | tRNA aspartic acid methyltransferase 1 | 487116 |
| TRIB2 | tribbles pseudokinase 2 | 403884 |
| TRIM37 | tripartite motif-containing 37 | 480575 |
| TRIM47 | tripartite motif-containing 47 | 609519 |
| TRIM9 | tripartite motif-containing 9 | 490687 |
| TRIOBP | TRIO and F-actin binding protein | 481263 |
| TRIP6 | thyroid hormone receptor interactor 6 | 479732 |
| TRMT11 | tRNA methyltransferase 11 homolog | 476285 |
| TROVE2 | TROVE domain family, member 2 | 478957 |
| TRPM3 | transient receptor potential cation channel, subfamily M, member 3 | 476326 |
| TRPM7 | transient receptor potential cation channel, subfamily M, member 7 | 478300 |
| TRPS1 | trichorhinophalangeal syndrome I | 482018 |
| TRPT1 | tRNA phosphotransferase 1 | 483765 |
| TSC22D4 | TSC22 domain family, member 4 | 479734 |
| TSHR | thyroid stimulating hormone receptor | 403968 |
| TSPAN12 | tetraspanin 12 | 612262 |
| TSPAN33 | tetraspanin 33 | 475198 |
| TSPAN4 | tetraspanin 4 | 611422 |
| TSPAN7 | tetraspanin 7 | 480879 |
| TSPO | translocator protein | 474475 |
| TSSK4 | testis specific serine kinase 4 | 480262 |
| TST | thiosulfate sulfurtransferase | 481275 |
| TTC12 | tetratricopeptide repeat domain 12 | 479434 |
| TTC14 | tetratricopeptide repeat domain 14 | 478641 |
| TTC19 | tetratricopeptide repeat domain 19 | 479516 |
| TTC23 | tetratricopeptide repeat domain 23 | 488710 |
| TTC28 | tetratricopeptide repeat domain 28 | 486337 |
| TTC32 | tetratricopeptide repeat domain 32 | 610581 |
| TTC8 | tetratricopeptide repeat domain 8 | 480413 |
| TTL | tubulin tyrosine ligase | 483065 |
| TTLL11 | tubulin tyrosine ligase like 11 | 491355 |
| TTPA | tocopherol (alpha) transfer protein | 403627 |
| TTYH1 | tweety family member 1 | 476386 |
| TTYH3 | tweety family member 3 | 489890 |
| TUBB2B | tubulin beta-2B chain | 478702 |
| TUBB6 | tubulin, beta 6 | 480213 |
| TULP3 | tubby like protein 3 | 611832 |
| TWF1 | twinfilin actin binding protein 1 | 486600 |
| TXN2 | thioredoxin 2 | 474519 |
| TXNDC9 | thioredoxin domain containing 9 | 474557 |
| TXNIP | thioredoxin interacting protein | 475829 |
| TXNRD1 | thioredoxin reductase 1 | 474536 |
| TYROBP | TYRO protein tyrosine kinase binding protein | 476477 |
| UBB | ubiquitin B | 479513 |
| UBC | ubiquitin C | 610457 |
| UBE2F | ubiquitin-conjugating enzyme E2F | 477421 |
| UBE2L3 | ubiquitin conjugating enzyme E2 L3 | 477572 |
| UBIAD1 | UbiA prenyltransferase domain containing 1 | 487446 |
| UBR2 | ubiquitin protein ligase E3 component n-recognin 2 | 474903 |
| UCK2 | uridine-cytidine kinase 2 | 608979 |
| UGDH | UDP-glucose dehydrogenase | 479107 |
| UGP2 | UDP-glucose pyrophosphorylase 2 | 474615 |
| UHRF1 | ubiquitin like with PHD and ring finger domains 1 | 611463 |
| UNC13A | unc-13 homolog A | 484833 |
| UNC13C | unc-13 homolog C | 487564 |
| UPP1 | uridine phosphorylase 1 | 480772 |
| USP1 | ubiquitin specific peptidase 1 | 479549 |
| USP18 | ubiquitin specific peptidase 18 | 486763 |
| USP19 | ubiquitin specific peptidase 19 | 476627 |
| USP2 | ubiquitin specific peptidase 2 | 608188 |
| USP24 | ubiquitin specific peptidase 24 | 479558 |
| USP40 | ubiquitin specific peptidase 40 | 486168 |
| USP53 | ubiquitin specific peptidase 53 | 487923 |
| USP54 | ubiquitin specific peptidase 54 | 479249 |
| UTP6 | UTP6, small subunit processome component | 480616 |
| UTRN | utrophin | 442965 |
| VAMP4 | vesicle-associated membrane protein 4 | 480074 |
| VAMP5 | vesicle-associated membrane protein 5 | 482823 |
| VAMP8 | vesicle-associated membrane protein 8 | 609784 |
| VAV3 | vav 3 guanine nucleotide exchange factor | 479921 |
| VCAM1 | vascular cell adhesion molecule 1 | 403982 |
| VCAN | versican | 488922 |
| VDR | vitamin D (1,25- dihydroxyvitamin D3) receptor | 486588 |
| VEGFA | vascular endothelial growth factor A | 403802 |
| VEGFC | vascular endothelial growth factor C | 482932 |
| VGF | VGF nerve growth factor inducible | 100687924 |
| VGLL4 | vestigial like family member 4 | 607588 |
| VIM | vimentin | 477991 |
| VIT | vitrin | 483032 |
| VNN1 | vanin 1 | 442973 |
| VPS53 | VPS53, GARP complex subunit | 491188 |
| VPS54 | VPS54, GARP complex subunit | 474616 |
| WARS | tryptophanyl-tRNA synthetase | 480435 |
| WDR26 | WD repeat domain 26 | 480115 |
| WDR61 | WD repeat domain 61 | 479068 |
| WDR78 | WD repeat domain 78 | 479538 |
| WEE1 | WEE1 G2 checkpoint kinase | 476847 |
| WFDC2 | WAP four-disulfide core domain 2 | 403919 |
| WIPI1 | WD repeat domain, phosphoinositide interacting 1 | 490899 |
| WNT3 | Wnt family member 3 | 609107 |
| WNT7A | wingless-type MMTV integration site family, member 7A | 607180 |
| WNT7B | wingless-type MMTV integration site family, member 7B | 481206 |
| WSB1 | WD repeat and SOCS box-containing 1 | 480619 |
| WWC1 | WW and C2 domain containing 1 | 479297 |
| WWC2 | WW and C2 domain containing 2 | 482920 |
| WWTR1 | WW domain containing transcription regulator 1 | 609743 |
| XAF1 | XIAP associated factor 1 | 606957 |
| XPOT | exportin for tRNA | 474427 |
| XPR1 | xenotropic and polytropic retrovirus receptor | 490301 |
| XRCC5 | X-ray repair cross complementing 5 | 478902 |
| XRN1 | 5'-3' exoribonuclease 1 | 477100 |
| YAP1 | Yes-associated protein 1 | 479465 |
| YWHAQ | tyrosine 3-monooxygenase/tryptophan 5-monooxygenase activation protein theta | 607060 |
| ZAP70 | zeta-chain (TCR) associated protein kinase | 100271860 |
| ZBTB20 | zinc finger and BTB domain containing 20 | 487987 |
| ZBTB37 | similar to Zinc finger and BTB domain containing protein 37 | 490336 |
| ZBTB41 | zinc finger and BTB domain containing 41 | 612864 |
| ZBTB45 | zinc finger and BTB domain containing 45 | 484222 |
| ZBTB7C | zinc finger and BTB domain containing 7C | 490573 |
| ZC3H6 | zinc finger CCCH-type containing 6 | 475751 |
| ZEB1 | zinc finger E-box binding homeobox 1 | 477966 |
| ZEB2 | zinc finger E-box binding homeobox 2 | 483909 |
| ZFAND1 | zinc finger, AN1-type domain 1 | 487018 |
| ZFAND3 | zinc finger, AN1-type domain 3 | 481776 |
| ZFP106 | zinc finger protein 106 | 478267 |
| ZFP28 | zinc finger protein 28 | 611144 |
| ZFP36L1 | zinc finger protein 36, C3H type-like 1 | 490748 |
| ZFP36L2 | zinc finger protein 36, C3H type-like 2 | 609810 |
| ZFP91 | zinc finger protein 91 | 475962 |
| ZFYVE21 | zinc finger, FYVE domain containing 21 | 480447 |
| ZHX1 | zinc fingers and homeoboxes 1 | 475089 |
| ZHX3 | zinc fingers and homeoboxes 3 | 485875 |
| ZIC2 | Zic family member 2 | 485533 |
| ZIC3 | Zic family member 3 | 492171 |
| ZMPSTE24 | zinc metallopeptidase STE24 | 482460 |
| ZNRF3 | zinc and ring finger 3 | 486339 |
| ZSWIM6 | zinc finger, SWIM-type containing 6 | 478068 |
| ZWINT | ZW10 interacting kinetochore protein | 477579 |
|  |  |  |

# Supplemental table S4: Astrocyte-related differentially expressed genes

| **Gene symbol** | **Entrez gene ID** | **Cluster** | **Group 2 vs. 1** | | **Group 3 vs. 1** | | **Group 4 vs. 1** | |
| --- | --- | --- | --- | --- | --- | --- | --- | --- |
|  |  |  | **Fold change** | ***q*-value** | **Fold change** | ***q*-value** | **Fold change** | ***q*-value** |
| ACSBG1 | 479067 | A | -2.70 | 0.0012 | -3.69 | 0.0000 | -1.70 | 0.1024 |
| ACSL5 | 477820 | B | 5.03 | 0.0000 | 6.97 | 0.0000 | 6.74 | 0.0000 |
| ANXA1 | 476322 | B | 1.93 | 0.0219 | 3.86 | 0.0000 | 5.13 | 0.0001 |
| ANXA2 | 403435 | B | 2.11 | 0.0255 | 5.27 | 0.0000 | 6.99 | 0.0001 |
| ATP1A2 | 488636 | A | -1.25 | 0.3625 | -2.13 | 0.0013 | -3.38 | 0.0005 |
| ATP1B2 | 489479 | A | -1.46 | 0.0258 | -1.66 | 0.0010 | -2.06 | 0.0013 |
| B2M | 100855741 | B | 5.35 | 0.0000 | 6.33 | 0.0000 | 4.93 | 0.0000 |
| BACE1 | 489390 | A | -1.54 | 0.0190 | -2.07 | 0.0001 | -1.62 | 0.0277 |
| BCAN | 612102 | A | -1.53 | 0.0333 | -2.72 | 0.0000 | -2.64 | 0.0004 |
| C1QA | 478194 | B | 8.57 | 0.0004 | 9.06 | 0.0001 | 2.59 | 0.1220 |
| C1S | 486714 | B | 8.26 | 0.0000 | 13.31 | 0.0000 | 9.20 | 0.0000 |
| C3 | 476728 | B | 4.52 | 0.0006 | 3.19 | 0.0001 | 4.93 | 0.0001 |
| CAV1 | 403980 | B | 1.59 | 0.0939 | 2.29 | 0.0015 | 3.51 | 0.0010 |
| CCL2 | 403981 | C | 11.98 | 0.0000 | 14.61 | 0.0000 | 8.28 | 0.0016 |
| CCL5 | 403522 | D | 67.15 | 0.0000 | 151.09 | 0.0000 | 70.52 | 0.0000 |
| CD44 | 403939 | B | 1.73 | 0.1811 | 5.07 | 0.0001 | 7.29 | 0.0006 |
| CDKN1A | 474890 | B | 2.11 | 0.0058 | 4.64 | 0.0000 | 3.13 | 0.0008 |
| CFLAR | 488471 | B | 2.11 | 0.0872 | 6.05 | 0.0001 | 2.08 | 0.0084 |
| CH25H | 100856263 | B | 4.12 | 0.0014 | 4.68 | 0.0001 | 2.48 | 0.0564 |
| CHI3L1 | 490222 | C | 34.48 | 0.0000 | 113.98 | 0.0000 | 45.33 | 0.0000 |
| CTSB | 486077 | B | 2.13 | 0.0222 | 3.74 | 0.0001 | 2.10 | 0.0079 |

| **Gene symbol** | **Entrez gene ID** | **Cluster** | **Group 2 vs. 1** | | **Group 3 vs. 1** | | **Group 4 vs. 1** | |
| --- | --- | --- | --- | --- | --- | --- | --- | --- |
|  |  |  | **Fold change** | ***q*-value** | **Fold change** | ***q*-value** | **Fold change** | ***q*-value** |
| CTSZ | 611983 | B | 1.14 | 0.4789 | 1.91 | 0.0016 | 2.44 | 0.0006 |
| CXCL10 | 478432 | D | 285.42 | 0.0000 | 385.62 | 0.0000 | 226.67 | 0.0000 |
| DDO | 475026 | A | -1.30 | 0.0897 | -2.12 | 0.0000 | -1.19 | 0.3529 |
| EPAS1 | 474578 | A | -1.82 | 0.0100 | -2.29 | 0.0002 | -1.72 | 0.0425 |
| FBXO2 | 478231 | A | -1.83 | 0.0072 | -2.04 | 0.0004 | -1.33 | 0.2521 |
| FLNC | 482266 | B | 1.32 | 0.4545 | 5.94 | 0.0000 | 8.16 | 0.0001 |
| GABRG1 | 100856619 | A | -2.05 | 0.0543 | -3.75 | 0.0003 | -3.56 | 0.0080 |
| GADD45B | 485069 | B | 6.19 | 0.0001 | 11.42 | 0.0000 | 5.95 | 0.0006 |
| GCH1 | 609393 | B | 1.59 | 0.2268 | 4.19 | 0.0002 | 3.01 | 0.0246 |
| GFAP | 480495 | B | 2.07 | 0.0033 | 1.89 | 0.0021 | 2.32 | 0.0043 |
| GNS | 474429 | B | 2.53 | 0.0014 | 2.35 | 0.0005 | 1.82 | 0.0276 |
| GPC4 | 492144 | B | -1.08 | 0.7347 | 1.59 | 0.0141 | 2.55 | 0.0012 |
| GRN | 480501 | B | 1.59 | 0.0582 | 2.54 | 0.0002 | 1.73 | 0.0653 |
| ICAM1 | 403975 | C | 11.32 | 0.0000 | 19.72 | 0.0000 | 11.31 | 0.0001 |
| IDO1 | 475574 | C | 22.74 | 0.0000 | 64.12 | 0.0000 | 30.64 | 0.0000 |
| IER3 | 481708 | B | 2.48 | 0.0010 | 3.36 | 0.0000 | 3.07 | 0.0007 |
| IFI44 | 490198 | C | 69.67 | 0.0000 | 78.05 | 0.0000 | 23.72 | 0.0000 |
| LGALS3 | 404021 | B | 1.47 | 0.3267 | 4.72 | 0.0001 | 11.39 | 0.0000 |
| LTBP1 | 475720 | B | 1.38 | 0.4070 | 2.59 | 0.0074 | 7.67 | 0.0003 |
| LTBR | 486728 | B | 1.71 | 0.0110 | 2.10 | 0.0002 | 1.97 | 0.0080 |
| MSN | 491924 | B | 2.30 | 0.0083 | 2.73 | 0.0004 | 4.31 | 0.0004 |

| **Gene symbol** | **Entrez gene ID** | **Cluster** | **Group 2 vs. 1** | | **Group 3 vs. 1** | | **Group 4 vs. 1** | |
| --- | --- | --- | --- | --- | --- | --- | --- | --- |
|  |  |  | **Fold change** | ***q-*value** | **Fold change** | ***q-*value** | **Fold change** | ***q*-value** |
| MT1H | 403768 | B | 1.54 | 0.0203 | 2.06 | 0.0001 | 1.72 | 0.0160 |
| NR4A1 | 403897 | A | -6.98 | 0.0021 | -11.04 | 0.0001 | -6.20 | 0.0109 |
| PARP9 | 488010 | C | 18.14 | 0.0000 | 29.63 | 0.0000 | 9.32 | 0.0000 |
| PDPN | 403886 | B | 3.58 | 0.0047 | 5.31 | 0.0001 | 5.03 | 0.0022 |
| PLA2G7 | 403848 | B | 1.65 | 0.0301 | 2.90 | 0.0000 | 1.71 | 0.0526 |
| PLEKHB1 | 608340 | A | -1.61 | 0.1460 | -3.59 | 0.0001 | -1.08 | 0.8543 |
| PPM1M | 484739 | B | 1.44 | 0.0227 | 1.68 | 0.0005 | 2.28 | 0.0002 |
| PSAP | 479240 | B | 1.74 | 0.0030 | 1.88 | 0.0002 | 2.06 | 0.0015 |
| PSMB8 | 474865 | C | 17.91 | 0.0000 | 45.76 | 0.0000 | 21.20 | 0.0000 |
| PSMB9 | 474867 | C | 5.72 | 0.0000 | 11.61 | 0.0000 | 6.20 | 0.0000 |
| PTGS2 | 442942 | B | 6.37 | 0.0017 | 4.95 | 0.0010 | 3.80 | 0.0371 |
| PYCARD | 100856347 | B | 4.84 | 0.0042 | 6.10 | 0.0003 | 2.67 | 0.1040 |
| RAB13 | 612294 | B | 1.89 | 0.0145 | 2.11 | 0.0012 | 2.51 | 0.0044 |
| RGS1 | 488585 | B | 4.44 | 0.0073 | 9.55 | 0.0000 | 11.46 | 0.0006 |
| RSAD2 | 609005 | C | 10.80 | 0.0000 | 25.01 | 0.0000 | 3.17 | 0.0155 |
| SDC4 | 485893 | B | 2.14 | 0.0070 | 2.65 | 0.0002 | 2.23 | 0.0156 |
| SERPING1 | 475966 | B | 7.74 | 0.0001 | 11.82 | 0.0000 | 5.88 | 0.0018 |
| SLC11A1 | 478909 | B | 6.30 | 0.0000 | 7.78 | 0.0000 | 4.91 | 0.0013 |
| SLC1A2 | 403750 | A | -1.20 | 0.4791 | -2.79 | 0.0001 | -2.09 | 0.0232 |
| SLC7A10 | 484599 | A | -1.82 | 0.0566 | -4.75 | 0.0000 | -2.96 | 0.0072 |
| STAT3 | 490967 | B | 1.85 | 0.0007 | 2.10 | 0.0001 | 1.50 | 0.0365 |

| **Gene symbol** | **Entrez gene ID** | **Cluster** | **Group 2 vs. 1** | | **Group 3 vs. 1** | | **Group 4 vs. 1** | |
| --- | --- | --- | --- | --- | --- | --- | --- | --- |
|  |  |  | **Fold change** | ***q*-value** | **Fold change** | ***q*-value** | **Fold change** | ***q*-value** |
| STX11 | 484019 | B | 2.94 | 0.0025 | 4.00 | 0.0000 | 2.26 | 0.0387 |
| TAPBP | 481740 | B | 3.96 | 0.0000 | 8.33 | 0.0000 | 2.22 | 0.0038 |
| TAPBPL | 486727 | B | 1.64 | 0.0774 | 2.94 | 0.0001 | 2.90 | 0.0039 |
| TGIF1 | 490537 | B | 1.54 | 0.1399 | 2.67 | 0.0006 | 2.90 | 0.0059 |
| THBS1 | 487486 | B | 2.09 | 0.0105 | 4.73 | 0.0000 | 3.56 | 0.0007 |
| TIMP1 | 403816 | C | 5.27 | 0.0005 | 15.98 | 0.0000 | 19.45 | 0.0000 |
| TLR2 | 448807 | B | 2.10 | 0.0377 | 2.84 | 0.0014 | 4.52 | 0.0015 |
| TLR3 | 482905 | B | 4.76 | 0.0013 | 5.87 | 0.0001 | 8.00 | 0.0005 |
| TNFRSF1A | 403634 | B | 2.11 | 0.0178 | 3.23 | 0.0001 | 2.35 | 0.0232 |
| TNFSF10 | 100174780 | B | 6.23 | 0.0001 | 7.47 | 0.0000 | 6.39 | 0.0001 |
| TOP2A | 480525 | C | 6.28 | 0.0001 | 14.03 | 0.0000 | 18.38 | 0.0000 |
| TRPM3 | 476326 | A | -2.28 | 0.0015 | -2.84 | 0.0000 | -2.81 | 0.0009 |
| LOC100856638 / UPP1 | 100856638 / 480772 | C | 11.70 | 0.0000 | 19.16 | 0.0000 | 8.07 | 0.0000 |
| USP18 | 486763 | C | 34.73 | 0.0000 | 39.68 | 0.0000 | 15.92 | 0.0000 |
| VCAM1 | 403982 | B | 3.27 | 0.0011 | 3.15 | 0.0002 | 3.61 | 0.0023 |
| VIM | 477991 | B | 1.62 | 0.0690 | 2.88 | 0.0001 | 4.96 | 0.0000 |
| WARS | 480435 | B | 1.79 | 0.0028 | 2.08 | 0.0001 | 1.95 | 0.0028 |
| XAF1 | 606957 | C | 15.65 | 0.0000 | 30.82 | 0.0000 | 16.69 | 0.0000 |

# Supplemental table S5: A1/A2-associated genes

| **Gene symbol** | **Phenotype** | **Group 2 vs. 1** | | **Group 3 vs. 1** | | **Group 4 vs. 1** | |
| --- | --- | --- | --- | --- | --- | --- | --- |
|  |  | **Fold change** | ***q*-value** | **Fold change** | ***q*-value** | **Fold change** | ***q*-value** |
| C1S | A1 | 5.59 | 0.0005 | 9.27 | 0.0000 | 7.46 | 0.1136 |
| TSPO | A1 | -1.39 | 0.5957 | -1.01 | 0.9768 | 1.38 | 0.5626 |
| PSMB8 | A1 | 17.28 | 0.0010 | 44.58 | 0.0000 | 21.20 | 0.0518 |
| FKBP5 | A1 | 1.93 | 0.2044 | 2.83 | 0.0853 | 3.06 | 0.0009 |
| CRISPLD | A1 | 1.72 | 0.1368 | 1.52 | 0.1736 | 1.39 | 0.5626 |
| GPX3 | A1 | -1.01 | 0.6821 | -1.01 | 0.5387 | -1.01 | 0.5626 |
| PSMB9 | A1 | 5.72 | 0.0123 | 11.61 | 0.0000 | 6.20 | 0.0213 |
| TGM2 | A1 | 1.07 | 0.9089 | 2.05 | 0.2981 | 2.29 | 0.2911 |
| TLR2 | A1 | 1.90 | 0.1337 | 2.32 | 0.0881 | 3.47 | 0.1831 |
| SULF2 | A1 | 1.14 | 0.5910 | -1.19 | 0.6673 | 1.24 | 0.6197 |
| IFI44 | A1 | 24.66 | 0.0000 | 31.01 | 0.0000 | 15.28 | 0.0000 |
| OLFM1 | A1 | -1.02 | 0.9131 | -1.01 | 0.9746 | -1.16 | 0.5626 |
| SRGN | A1 | 2.29 | 0.2460 | 3.27 | 0.0976 | 3.01 | 0.0125 |
| TAPBP | A1 | 3.96 | 0.0888 | 8.33 | 0.0018 | 1.74 | 0.0041 |
| ACSL5 | A1 | 5.03 | 0.0031 | 6.97 | 0.0005 | 6.74 | 0.1777 |
| ANGPT1 | A1 | -1.61 | 0.4638 | -2.19 | 0.1022 | -1.82 | 0.6436 |
| B2M | A1 | 3.14 | 0.0000 | 3.45 | 0.0000 | 2.94 | 0.0016 |
| LY6E | A1 | -1.15 | 0.5933 | 1.00 | 0.9905 | 1.02 | 0.9268 |
| HMGA1 | A1 | -1.19 | 0.3293 | 1.10 | 0.6881 | 1.29 | 0.5176 |
| DCN | A1 | -1.15 | 0.7442 | -2.10 | 0.1941 | 2.42 | 0.5626 |
| XAF1 | A1 | 15.65 | 0.0173 | 30.82 | 0.0001 | 16.69 | 0.0662 |
| GSR | A1 | 1.49 | 0.0567 | 1.69 | 0.0042 | 1.57 | 0.3465 |
| SLC43A3 | A1 | 2.42 | 0.0692 | 2.26 | 0.1980 | 2.30 | 0.5626 |
| SORBS1 | A1 | -1.09 | 0.8729 | 1.02 | 0.9612 | -1.32 | 0.6934 |
| ENDOU | A1 | -1.13 | 0.8372 | 1.83 | 0.5270 | -1.24 | 0.4837 |
| AMIGO2 | A1 | -1.51 | 0.5910 | -1.79 | 0.3223 | 1.06 | 0.9584 |
| NFASC | A1 | -1.84 | 0.0733 | -2.18 | 0.0119 | -1.84 | 0.2126 |
| MAP3K6 | A1 | -1.00 | 0.9183 | -1.00 | 0.5387 | -1.00 | 0.5626 |
| SLC22A4 | A1 | 1.27 | 0.6046 | 1.77 | 0.0926 | 1.73 | 0.0250 |
| TAPBPL | A1 | 1.64 | 0.4139 | 2.94 | 0.1133 | 2.90 | 0.1054 |
| PLIN4 | A1 | 1.04 | 0.5933 | 1.02 | 0.5387 | 1.00 | 0.6164 |
| KCTD1 | A1 | 1.04 | 0.8220 | 1.03 | 0.8859 | -1.12 | 0.5393 |
| TNFAIP2 | A1 | 1.16 | 0.5898 | 1.04 | 0.5387 | 1.00 | 1.0000 |
| IL1R1 | A1 | 1.21 | 0.5910 | 1.20 | 0.5741 | 1.18 | 0.5626 |
| SEMA4C | A1 | 1.03 | 0.6507 | 1.01 | 0.7323 | -1.01 | 0.5626 |
| SLC1A5 | A1 | 1.59 | 0.3413 | 1.94 | 0.1150 | 1.79 | 0.5626 |
| FGL2 | A2 | 1.72 | 0.5608 | 2.85 | 0.0232 | 2.96 | 0.3402 |
| COL12A1 | A2 | -1.49 | 0.2992 | -2.02 | 0.1274 | 1.18 | 0.8358 |
| MRPS6 | A2 | -1.27 | 0.0662 | -1.39 | 0.0527 | 1.01 | 0.9795 |
| S100A11 | A2 | 1.62 | 0.2785 | 1.72 | 0.1580 | 2.11 | 0.5626 |
| ESD | A2 | 1.01 | 0.9680 | 1.06 | 0.6893 | 1.11 | 0.5532 |
| DPYSL3 | A2 | -1.29 | 0.5642 | -2.22 | 0.0105 | 1.07 | 0.9005 |
| TLR4 | A2 | 1.72 | 0.0860 | 2.38 | 0.0529 | 1.56 | 0.5626 |
| CAV1 | A2 | 1.43 | 0.2036 | 2.22 | 0.1268 | 3.26 | 0.3561 |
| ASNS | A2 | -1.00 | 0.9893 | 1.10 | 0.5824 | 1.37 | 0.5626 |
| GADD45A | A2 | 1.67 | 0.0774 | 2.03 | 0.0406 | 1.77 | 0.3990 |
| CTGF | A2 | -1.03 | 0.9492 | 1.63 | 0.1135 | 1.47 | 0.5921 |
| COL6A1 | A2 | 1.06 | 0.6253 | 1.06 | 0.5826 | -1.01 | 0.5626 |
| SRXN1 | A2 | 1.14 | 0.5196 | 1.31 | 0.1326 | 1.23 | 0.4314 |
| PVR | A2 | -1.07 | 0.7581 | 1.10 | 0.6970 | 1.07 | 0.8273 |
| CLCF1 | A2 | -1.05 | 0.5910 | 1.31 | 0.5387 | 1.25 | 0.6628 |
| ANXA2 | A2 | 1.91 | 0.1273 | 4.38 | 0.0040 | 5.99 | 0.3216 |
| CYP1B1 | A2 | -1.08 | 0.9055 | 2.12 | 0.0943 | 3.38 | 0.5292 |
| MET | A2 | 1.06 | 0.7818 | -1.02 | 0.7390 | 1.02 | 0.8638 |
| FLNA | A2 | -1.11 | 0.6105 | 1.12 | 0.3786 | 1.22 | 0.6280 |
| TXNRD1 | A2 | -1.13 | 0.5910 | -1.08 | 0.5492 | 1.06 | 0.8823 |
| MSN | A2 | 1.82 | 0.0147 | 2.37 | 0.0040 | 2.55 | 0.3615 |
| FLNC | A2 | 1.32 | 0.5094 | 5.94 | 0.0401 | 8.16 | 0.4332 |
| CH25H | A2 | 4.12 | 0.0214 | 4.68 | 0.0262 | 2.48 | 0.5626 |
| BDNF | A2 | -1.97 | 0.2651 | -1.78 | 0.0385 | -2.02 | 0.4728 |
| NHP2 | A2 | -1.01 | 0.9373 | -1.00 | 0.9875 | 1.15 | 0.5219 |
| COL6A2 | A2 | -1.01 | 0.5910 | -1.01 | 0.5387 | -1.01 | 0.5626 |
| CAMK2D | A2 | 1.32 | 0.2233 | 1.33 | 0.3358 | 1.08 | 0.6932 |
| ACTN1 | A2 | 1.03 | 0.7221 | 1.07 | 0.5752 | 1.16 | 0.6613 |
| AHR | A2 | 1.16 | 0.4628 | 1.45 | 0.1565 | 1.42 | 0.5515 |
| SULF1 | A2 | -1.62 | 0.4412 | -1.16 | 0.7441 | 1.53 | 0.6164 |
| SYT4 | A2 | 1.05 | 0.6172 | 1.15 | 0.2052 | -1.02 | 0.9133 |
| B3GNT5 | A2 | 1.19 | 0.5172 | 1.12 | 0.7238 | 1.28 | 0.7562 |
| AHNAK | A2 | -1.08 | 0.7646 | 1.07 | 0.8251 | 1.42 | 0.5438 |
| LRRC59 | A2 | -1.01 | 0.9514 | 1.05 | 0.7707 | -1.11 | 0.5263 |
| THBS1 | A2 | 2.09 | 0.0211 | 4.73 | 0.0000 | 3.56 | 0.5211 |
| AKAP12 | A2 | -1.07 | 0.7635 | -1.03 | 0.8390 | 1.09 | 0.7964 |
| AJUBA | A2 | -1.10 | 0.5764 | -1.03 | 0.8102 | 1.01 | 0.9543 |
| LGALS1 | A2 | -1.29 | 0.5910 | 1.20 | 0.6491 | 2.36 | 0.3873 |
| SPATA13 | A2 | 1.13 | 0.7712 | 1.39 | 0.4519 | 1.57 | 0.5236 |
| PDE3B | A2 | -1.07 | 0.7483 | 1.15 | 0.4214 | -1.05 | 0.6614 |
| PLA2G4A | A2 | 2.02 | 0.3875 | 1.75 | 0.4827 | 2.49 | 0.2189 |
| STX11 | A2 | 2.94 | 0.0004 | 4.00 | 0.0250 | 2.26 | 0.5626 |
| KLF6 | A2 | -1.06 | 0.8235 | 1.19 | 0.5084 | 1.22 | 0.6493 |
| LMNA | A2 | -1.06 | 0.6441 | 1.24 | 0.4478 | 1.20 | 0.6164 |
| NUPR1 | A2 | -1.18 | 0.7711 | 2.31 | 0.0529 | 1.67 | 0.5626 |
| MCL1 | A2 | 1.45 | 0.0674 | 1.94 | 0.0055 | 1.66 | 0.4117 |
| ANXA7 | A2 | 1.13 | 0.5517 | 1.62 | 0.0002 | 1.24 | 0.2711 |
| PTGS2 | A2 | 6.37 | 0.1581 | 4.95 | 0.0543 | 3.80 | 0.2691 |
| BDKRB2 | A2 | 1.02 | 0.5933 | 1.00 | 1.0000 | 1.00 | 1.0000 |
| IL6 | A2 | 2.42 | 0.5820 | 1.32 | 0.3547 | 1.06 | 0.6372 |
| HSPB1 | A2 | -1.04 | 0.9176 | -1.02 | 0.9445 | -1.08 | 0.8026 |
| CCL2 | A2 | 11.98 | 0.0664 | 14.61 | 0.0003 | 8.28 | 0.4485 |
| SOCS3 | A2 | 1.11 | 0.5461 | 1.01 | 0.4117 | -1.00 | 0.9855 |
| FOSL2 | A2 | 1.17 | 0.8331 | 1.59 | 0.2861 | 1.80 | 0.5471 |
| S100A6 | A2 | -1.02 | 0.9682 | 1.95 | 0.3147 | 3.73 | 0.0374 |
| PRSS23 | A2 | -1.26 | 0.6161 | -1.04 | 0.9216 | 1.55 | 0.4555 |
| SERPINE | A2 | 1.19 | 0.4160 | 1.08 | 0.6235 | 1.28 | 0.5626 |
| HMOX1 | A2 | -1.02 | 0.5910 | -1.02 | 0.5387 | -1.02 | 0.5626 |
| PLP2 | A2 | 1.16 | 0.6274 | 2.20 | 0.1692 | 3.04 | 0.4270 |
| NES | A2 | -1.16 | 0.2916 | -1.05 | 0.7713 | 1.45 | 0.0326 |
| LITAF | A2 | 1.26 | 0.5910 | 2.18 | 0.1315 | 2.88 | 0.3800 |
| LRRFIP1 | A2 | -1.15 | 0.6172 | -1.08 | 0.7212 | -1.11 | 0.6919 |
| FBLN5 | A2 | 1.02 | 0.9557 | 1.07 | 0.8325 | 1.53 | 0.4080 |
| ZWINT | A2 | -1.24 | 0.5910 | -1.45 | 0.3179 | -1.05 | 0.9263 |
| ANXA1 | A2 | 1.65 | 0.1281 | 3.38 | 0.0106 | 4.62 | 0.2987 |
| CACNG5 | A2 | -1.02 | 0.8801 | 1.07 | 0.6454 | -1.10 | 0.2528 |
| LGALS3 | A2 | 1.42 | 0.4326 | 4.47 | 0.0853 | 9.08 | 0.1957 |
| BCAT1 | A2 | -1.14 | 0.5910 | -1.51 | 0.0969 | -1.02 | 0.9657 |
| SLC7A1 | A2 | 1.01 | 0.9310 | -1.03 | 0.5387 | -1.02 | 0.7744 |
| LONRF1 | A2 | 1.04 | 0.8235 | 1.14 | 0.5387 | -1.20 | 0.6295 |
| ADAMTS5 | A2 | -1.07 | 0.5910 | 1.02 | 0.9284 | 1.17 | 0.7164 |
| VCAN | A2 | 1.06 | 0.8279 | -1.09 | 0.7569 | 1.17 | 0.6690 |
| MTHFD2 | A2 | -1.06 | 0.8451 | -1.26 | 0.3325 | -1.26 | 0.5594 |
| ANXA3 | A2 | -1.40 | 0.6103 | -1.47 | 0.5264 | 1.67 | 0.3245 |
| OLFML3 | A2 | -1.22 | 0.4076 | -1.16 | 0.4776 | -1.09 | 0.7352 |
| CHAC1 | A2 | 1.05 | 0.7796 | -1.01 | 0.8892 | 1.13 | 0.6625 |
| NAV2 | A2 | -1.41 | 0.5498 | -1.72 | 0.1660 | -1.69 | 0.0029 |
| KLHDC8A | A2 | -1.29 | 0.5933 | -1.43 | 0.2900 | -1.09 | 0.9287 |
| NETO2 | A2 | -1.17 | 0.7269 | -2.30 | 0.0923 | -1.72 | 0.4424 |
| RNF19B | A2 | -1.07 | 0.6103 | -1.14 | 0.4382 | -1.15 | 0.4638 |
| CCND1 | A2 | 1.28 | 0.5910 | 1.10 | 0.8430 | -1.10 | 0.8990 |
| GPX1 | A2 | 1.28 | 0.0079 | 1.21 | 0.0775 | 1.18 | 0.5626 |
| TGIF1 | A2 | 1.54 | 0.5620 | 2.67 | 0.0264 | 2.90 | 0.2625 |
| PCBP3 | A2 | -1.32 | 0.5910 | -1.40 | 0.0773 | -1.69 | 0.3556 |
| ECM1 | A2 | -1.40 | 0.4490 | -1.25 | 0.5219 | -1.32 | 0.7487 |
| IGFBP3 | A2 | 1.05 | 0.5910 | -1.00 | 0.5172 | -1.00 | 0.5626 |
| NOP58 | A2 | -1.12 | 0.0584 | 1.08 | 0.5387 | -1.04 | 0.6209 |
| LIF | A2 | -1.00 | 0.5409 | -1.00 | 0.4517 | -1.00 | 0.5313 |
| ADAMTS4 | A2 | 1.47 | 0.5906 | -1.06 | 0.4596 | 1.64 | 0.6164 |
| TMEM74 | A2 | 1.00 | 0.5933 | 1.00 | 1.0000 | 1.00 | 1.0000 |
| ADAM12 | A2 | -1.04 | 0.5910 | -1.04 | 0.5387 | -1.03 | 0.6636 |
| FOSL1 | A2 | 1.00 | 1.0000 | 1.00 | 1.0000 | 1.00 | 1.0000 |
| EMP1 | A2 | 1.01 | 0.5910 | 1.26 | 0.4425 | -1.00 | 0.5626 |
| UCK2 | A2 | 1.30 | 0.5910 | 1.03 | 0.9437 | 1.23 | 0.5626 |
| VGF | A2 | -1.02 | 0.5910 | -1.02 | 0.6149 | 1.05 | 0.7246 |
| GDF15 | A2 | -1.00 | 0.5910 | -1.00 | 0.5387 | -1.00 | 0.5626 |
| GCH1 | A2 | 1.59 | 0.4629 | 4.19 | 0.1232 | 3.01 | 0.5325 |
| FSCN1 | A2 | -1.29 | 0.6450 | -3.13 | 0.1127 | -1.67 | 0.5626 |
| TGFBI | A2 | -1.02 | 0.6459 | 1.10 | 0.6073 | -1.03 | 0.5626 |
| SHISA6 | A2 | 1.07 | 0.5933 | 1.02 | 0.8049 | 1.02 | 0.8254 |
| RNF125 | A2 | -1.02 | 0.5395 | -1.02 | 0.4895 | -1.02 | 0.5324 |
| AHNAK2 | A2 | 1.00 | 1.0000 | 1.00 | 0.5387 | 1.04 | 0.6164 |
| GADD45B | A2 | 6.19 | 0.0005 | 11.42 | 0.0000 | 5.95 | 0.0139 |
| SBNO2 | A2 | 1.00 | 1.0000 | 1.00 | 0.5387 | 1.00 | 1.0000 |
| CDT1 | A2 | -1.04 | 0.8619 | -1.01 | 0.9869 | -1.07 | 0.7465 |
| SLC44A3 | A2 | 1.07 | 0.8552 | 1.20 | 0.5387 | 1.08 | 0.7958 |
| FAM129B | A2 | -1.00 | 0.5910 | 1.00 | 0.7811 | 1.05 | 0.6225 |
| STEAP1 | A2 | -1.75 | 0.1602 | -1.47 | 0.4201 | 1.01 | 0.9904 |
| OCIAD2 | A2 | -1.25 | 0.5258 | -1.00 | 0.9928 | 1.52 | 0.2965 |
| CDK6 | A2 | 1.01 | 0.9549 | 1.15 | 0.5499 | -1.04 | 0.8793 |
| MEST | A2 | 1.01 | 0.9805 | -1.70 | 0.0719 | 1.14 | 0.8597 |
| ODC1 | A2 | 1.21 | 0.5701 | 1.25 | 0.4876 | 1.23 | 0.5626 |
| GRB10 | A2 | 1.11 | 0.6068 | 1.17 | 0.5275 | 1.09 | 0.6200 |
| PAPPA | A2 | -1.00 | 0.5910 | -1.00 | 0.5387 | -1.00 | 0.5626 |
| KLF5 | A2 | -1.00 | 0.9954 | -1.06 | 0.8162 | -1.23 | 0.5335 |
| SPHK1 | A2 | -1.04 | 0.8818 | 1.04 | 0.8013 | 1.22 | 0.5505 |
| THBD | A2 | 1.01 | 0.8800 | -1.02 | 0.5129 | -1.02 | 0.5626 |
